# Supplementary material for: Integrating genetic regulation and single-cell expression with GWAS prioritizes causal genes and cell types for glaucoma
Source: Nat Commun. 2024 Jan 9;15:396. doi: 10.1038/s41467-023-44380-y (PMC10776627; doi:10.1038/s41467-023-44380-y)
Supplement: Supplementary file 1 — Supplementary Information [file 41467_2023_44380_MOESM1_ESM.pdf]

Supplementary Material for manuscript:

Title: Integrating genetic regulation and single-cell expression with GWAS prioritizes causal genes and cell types for glaucoma

Author list:

Andrew R. Hamel[1,2,3], Wenjun Yan[4,17], John M. Rouhana[1,2,3,17], Aboozar Monovarfeshani[4,17], Xinyi Jiang[5,6], Puja A. Mehta[1,2,3], Jayshree Advani[7], Yuyang Luo[1,2,3], Qingnan Liang[8], Skanda Rajasundaram[2,9,10], Arushi Shrivastava[1,2,3], Katherine Duchinski[1,2,3,11], Sreekar Mantena[1,12], Jiali Wang[1,2,3], Tavé van Zyl[4,13], Louis R. Pasquale[14], Anand Swaroop[7], Puya Gharahkhani[15], Anthony P. Khawaja[16], Stuart MacGregor[15], International Glaucoma Genetics Consortium (IGGC)[*], Rui Chen[8], Veronique Vitart[5], Joshua R. Sanes[4], Janey L. Wiggs[1,2,3], Ayellet V. Segrè[1,2,3,#]

Affiliations:

[1]Ocular Genomics Institute, Department of Ophthalmology, Massachusetts Eye and Ear, Boston, MA, USA

[2]Department of Ophthalmology, Harvard Medical School, Boston, MA, USA

[3]Broad Institute of Harvard and MIT, Cambridge, MA, USA

[4]Department of Molecular and Cellular Biology and Center for Brain Science, Harvard University, Cambridge, MA, USA

[5]MRC Human Genetics Unit, Institute of Genetics and Cancer, The University of Edinburgh, Edinburgh, UK

[6]Centre for Genomic and Experimental Medicine, Institute of Genetics and Molecular Medicine, The University of Edinburgh, Edinburgh, UK

[7]Neurobiology, Neurodegeneration and Repair Laboratory, National Eye Institute, National Institutes of Health, Bethesda, Maryland, USA

[8]Department of Molecular and Human Genetics, Baylor College of Medicine, TX, USA

[9]Centre for Evidence-Based Medicine, University of Oxford, Oxford, UK

[10]Faculty of Medicine, Imperial College London, London, UK

[11]Bioinformatics and Integrative Genomics (BIG) PhD Program, Harvard Medical School, Boston, MA, USA

[12]Harvard/MIT MD-PhD Program, Harvard Medical School, Boston, MA, USA

[13]Department of Ophthalmology and Visual Sciences, Yale School of Medicine, New Haven, CT, USA

[14]Department of Ophthalmology, Icahn School of Medicine at Mount Sinai, New York, NY, USA

[15]QIMR Berghofer Medical Research Institute, Brisbane, Queensland, 4029, Australia

[16]NIHR Biomedical Research Centre, Moorfields Eye Hospital NHS Foundation Trust and UCL Institute of Ophthalmology, London, UK

[17]These authors contributed equally

*A list of authors and their affiliations appears at the end of the paper

[#] Correspondence to be addressed to Ayellet V. Segrè: ayellet_segre@meei.harvard.edu

Supplementary Figure 1. e/sQTLs enriched for hundreds of new POAG and IOP associations

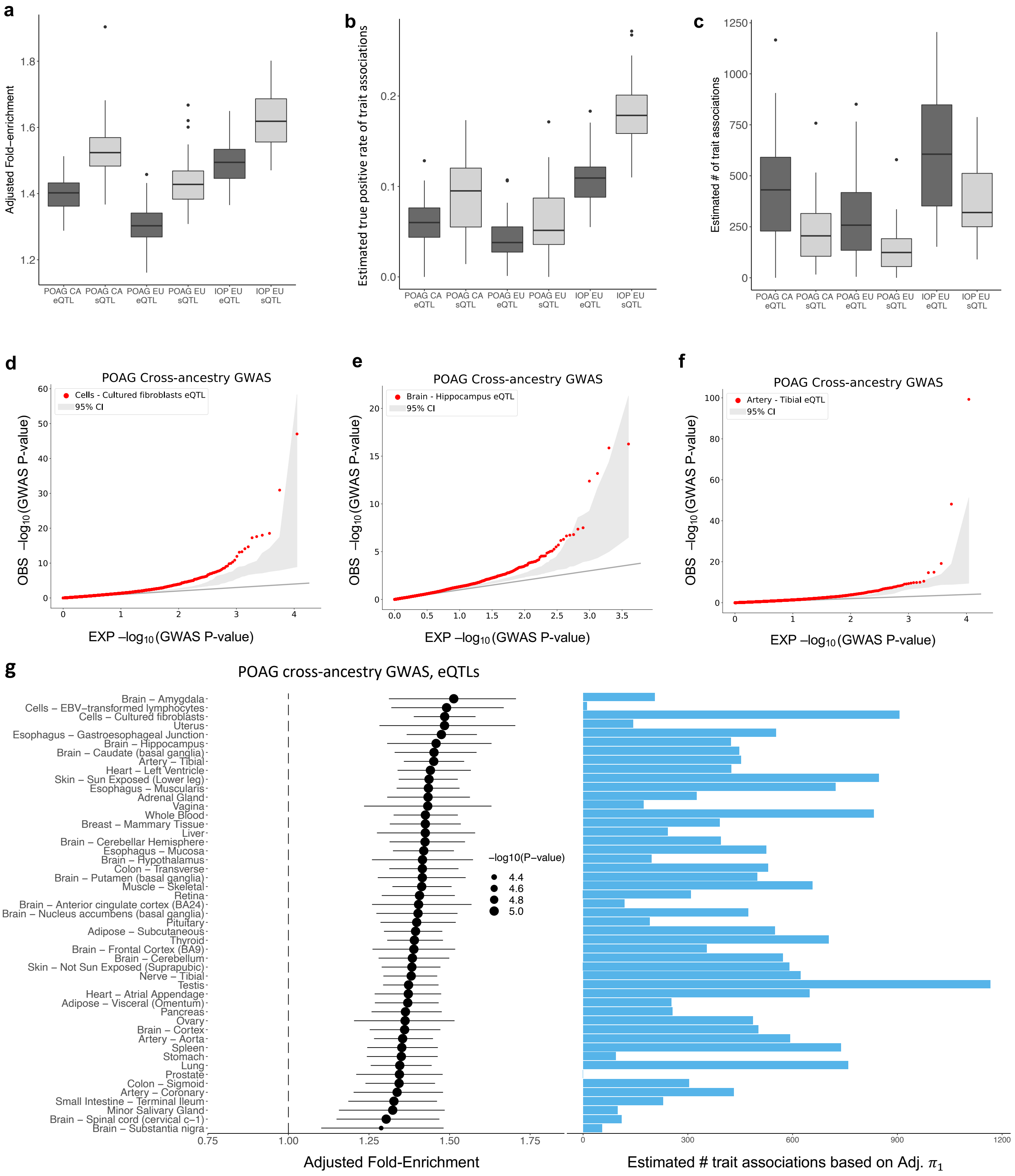

Supplementary Figure 1. e/sQTLs enriched for hundreds of new POAG and IOP associations. a-c, Boxplots showing the distributions of three *QTLEnrich* summary statistics for POAG cross-ancestry GWAS, POAG European (EUR) ancestry GWAS, and IOP GWAS for the significant tissue-trait pairs that passed Bonferroni correction for testing enrichment of trait associations amongst eQTLs (dark grey) and sQTLs (light grey) from 49 GTEx tissues and retina eQTLs: adjusted fold-enrichment (**a**), estimated true positive rate of trait associations among e/sQTLs (Adjusted π_1) (**b**), and estimated number of trait associations based on adjusted π_1 **(c)**. The center lines in the box plots depict the median value and the box edges the interquartile range. **d-f** Q-Q plots of -$\log_{10}$(P-value) of POAG cross-ancestry GWAS for the significant best eQTL per eGene set (FDR < 0.05) in top enriched tissues (red points): Cells - Cultured fibroblasts **(d),** Brain - Hippocampus **(e),** and Artery Tibial **(f)** compared to GWAS p-values of 1,000 confounder-matched null variant sets (light grey) generated by *QTLEnrich*. **g**, Forest plots (left panel) and barplots (right panel) of all tissues whose eQTLs were significantly enriched (Bonferroni correction) for POAG cross-ancestry GWAS associations based on *QTLEnrich*. Points indicate the adjusted fold-enrichment scaled by -log10(Enrichment P-value) and the lines represent 95% confidence intervals. Barplots show estimated number of e/sQTLs that are likely true trait associations per enriched tissue using an adjusted true positive rate, π_1 approach. The lack of estimated number of POAG associations among prostate eQTLs is likely due to deflation of the GWAS p-values of the prostate eQTL set (Methods).

Supplementary Figure 2. POAG and IOP trait associations amongst GTEx tissue and retina e/sQTLs

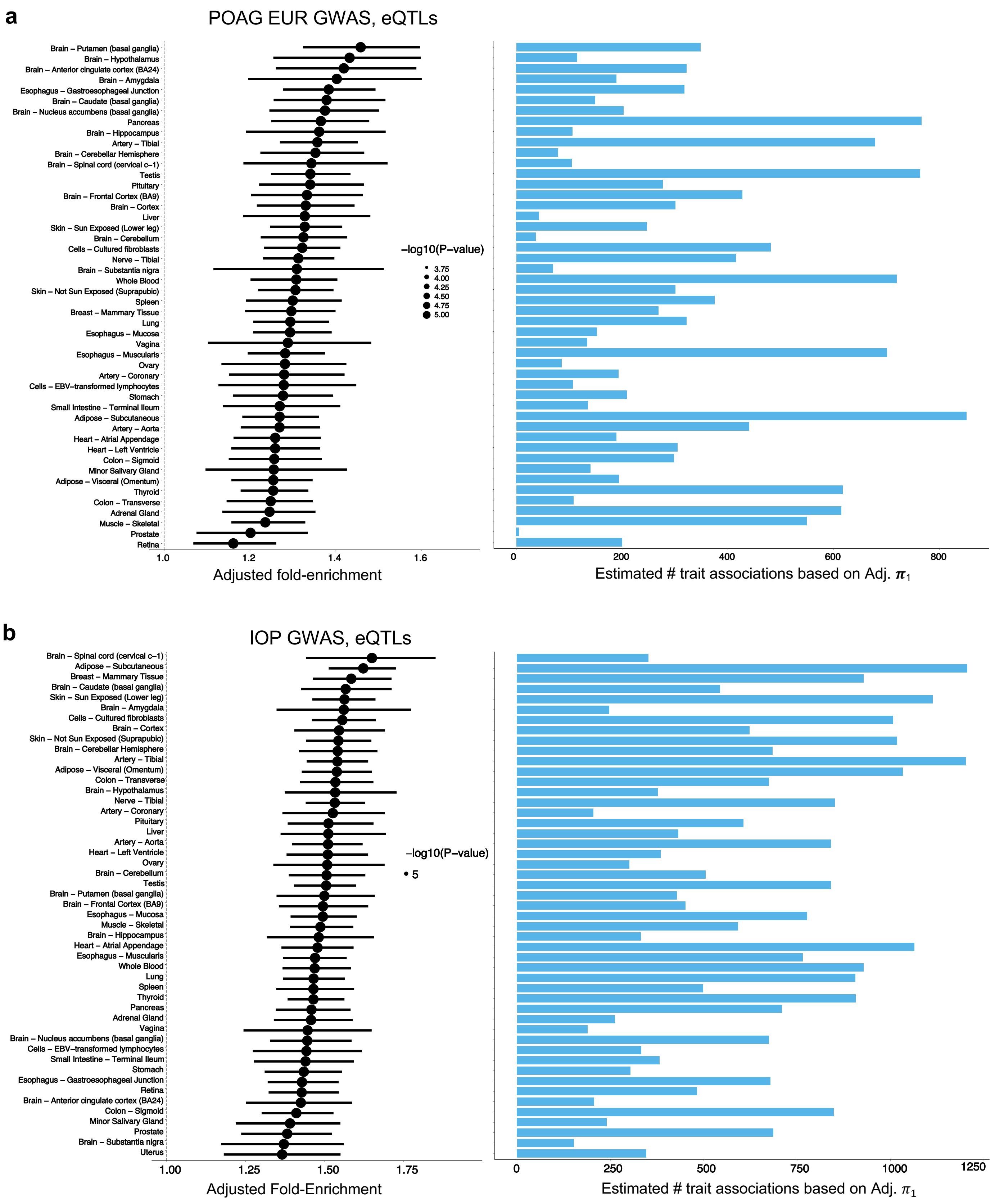

Supplementary Figure 2. POAG and IOP trait associations amongst GTEx tissue and retina e/sQTLs

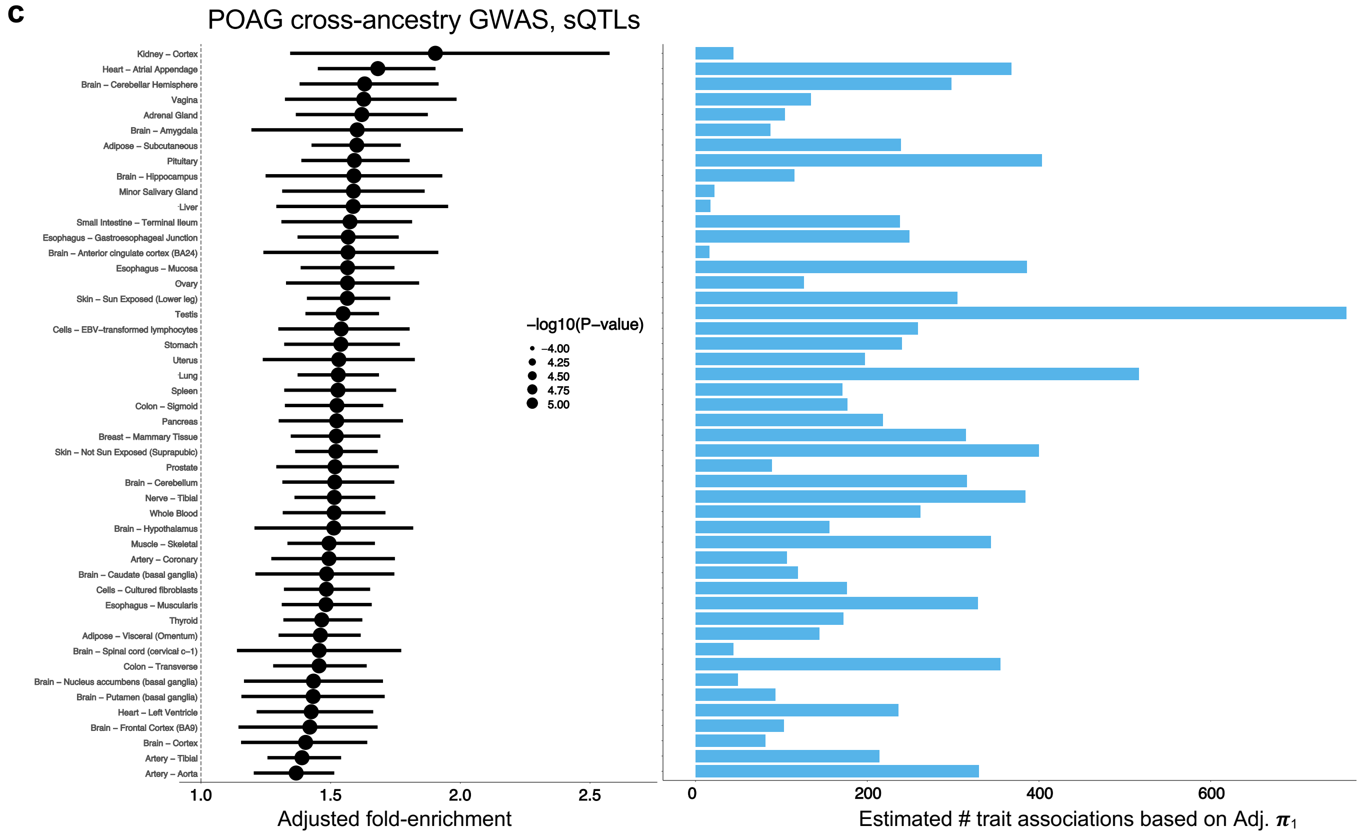

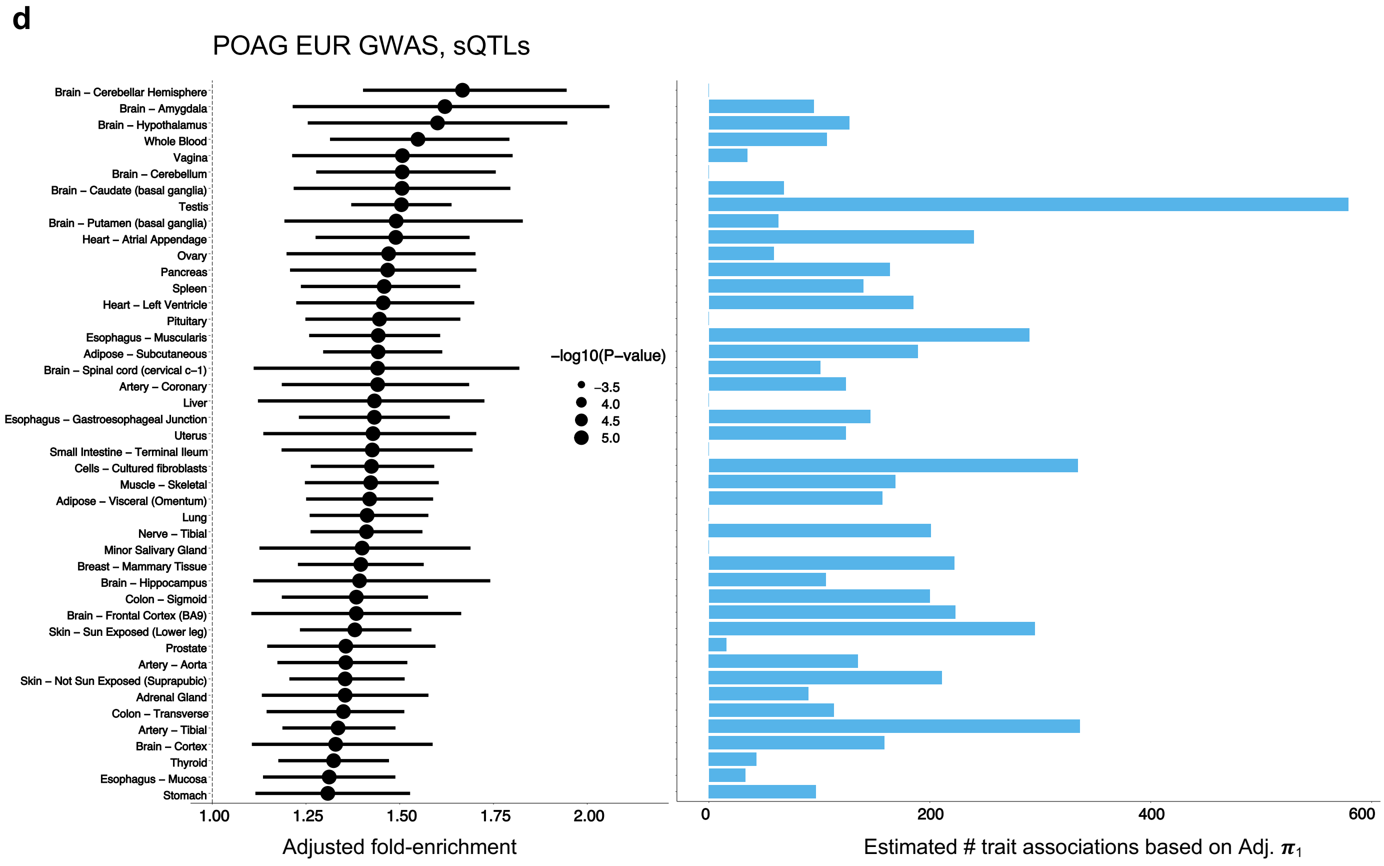

Supplementary Figure 2. POAG and IOP trait associations amongst GTEx tissue and retina e/sQTLs

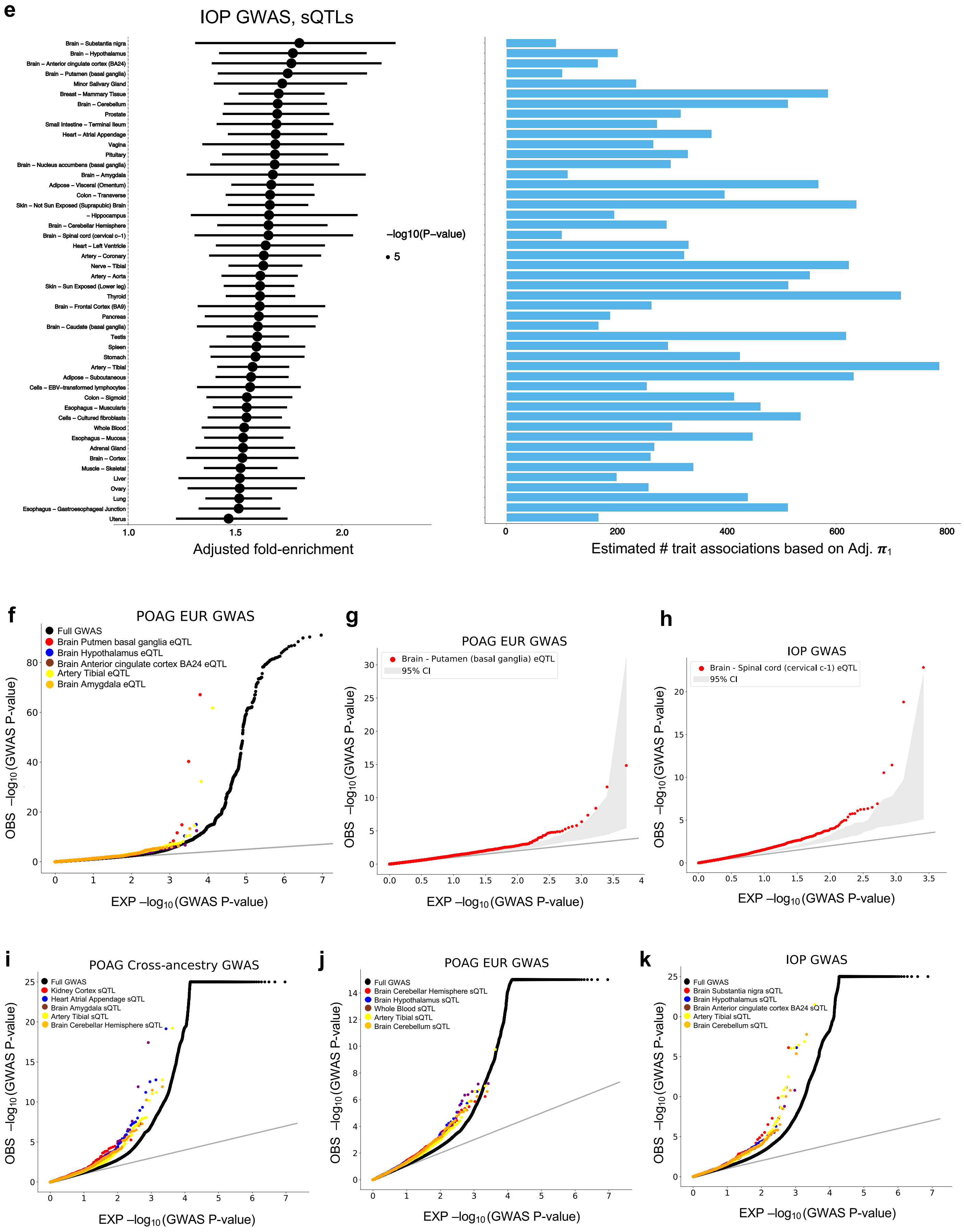

Supplementary Figure 2. POAG and IOP trait associations amongst GTEx tissues and retina e/sQTLs. a-e, Forest plots (left panel) and barplots (right panel) of all tissues whose eQTLs were significantly enriched (Bonferroni correction) for POAG European (**a**) or IOP (**b**) GWAS associations, or whose sQTLs were enriched for POAG cross-ancestry (**c**), POAG European (**d**), or IOP (**e**) associations based on *QTLEnrich*. Points indicate the adjusted fold-enrichment scaled by -log10(Enrichment P-value) and the lines represent 95% confidence intervals. Barplots show estimated number of e/sQTLs that are likely true trait associations per enriched tissue using an adjusted true positive rate, $\boldsymbol{\pi}_1$ approach. For several tissues with significant enrichment in (**d)**, the estimated number of POAG EUR associations among sQTLs was zero (e.g., brain cerebellum or pituitary). This is likely due to deflation of the GWAS p-values of the sQTL set at the higher end of p-values, that are used to estimate the true negative rate (see Methods). When considering only the top ranked sQTLs with GWAS P<0.05 in these tissue, a lower bound of 25-88 of the sQTLs were estimated to be true POAG associations (Supplementary Data 3). **f,** Quantile-quantile (Q-Q) plot of POAG European GWAS -$\log_{10}$(P-value) compared to expectation, assuming a null uniform distribution, for the eQTLs (best eQTL per eGene set with FDR < 0.05) in the most significantly enriched tissues based on adjusted fold-enrichment (colored points), compared to all variants in the GWAS (black points). Grey line represents the diagonal. **g-h,** Q-Q plot of POAG European (**g**) or IOP (**h**) GWAS -$\log_{10}$(P-value) of the best eQTL per eGene set in the top ranked tissue per trait based on adjusted fold-enrichment (red points), compared to GWAS p-values of 1,000 confounder-matched null variant sets (light grey) generated by *QTLEnrich*. **i-k**, Q-Q plot of POAG Cross-ancestry (**i**), POAG European (**j**), and IOP (**k**) GWAS -$\log_{10}$(P-value) compared to expectation for the sQTLs (best sQTL per sGene at FDR<0.05) in the top enriched tissues per trait based on adjusted fold-enrichment compared to all variants tested in the GWAS (black).

Supplementary Figure 3. Examples of potential false positive colocalization results that were filtered out

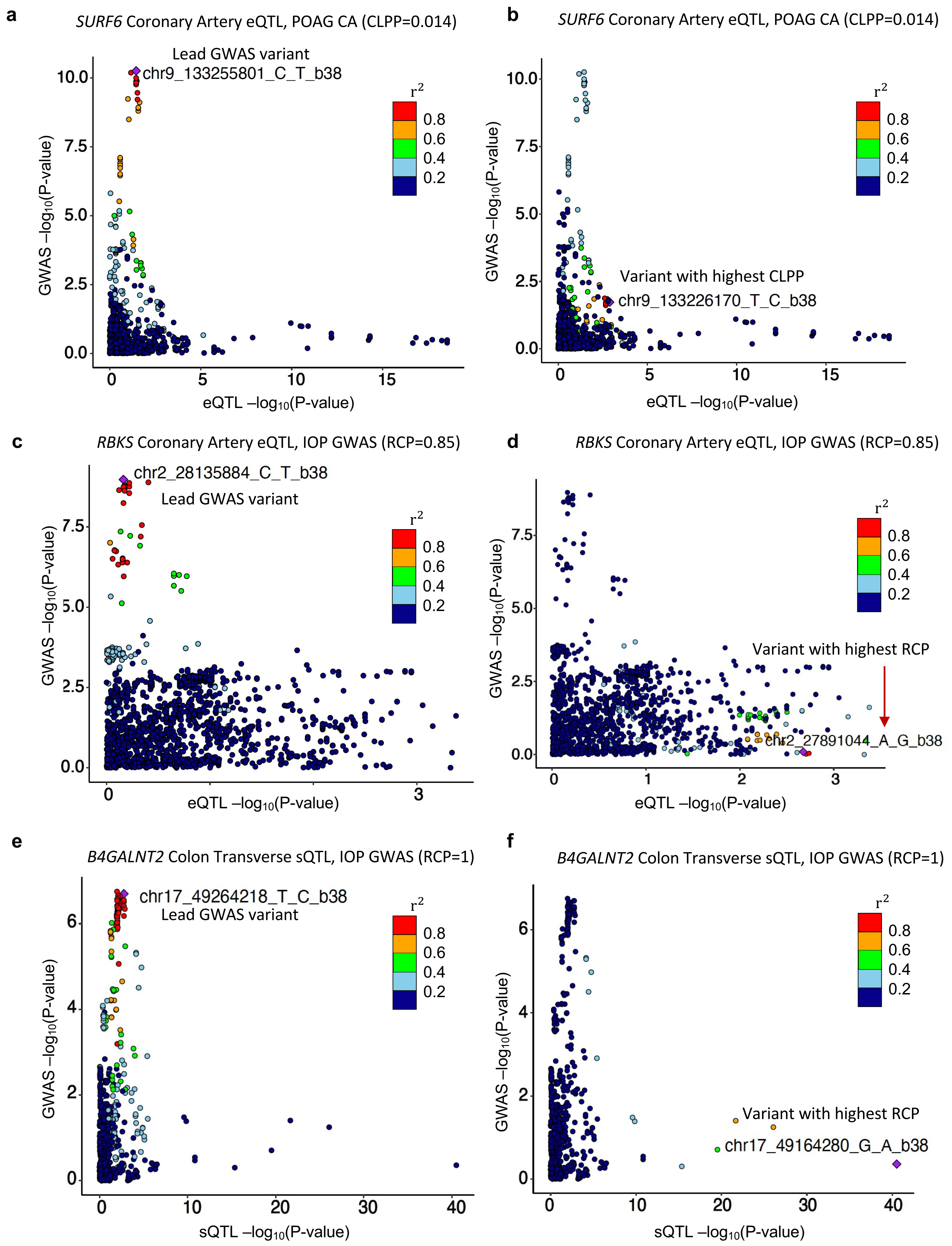

Supplementary Figure 3. Examples of potential false positive colocalization results that were filtered out. a,b LocusCompare plots of -$\log_{10}$ P-value of the POAG cross-ancestry (CA) GWAS as a function of the -$\log_{10}$ P-value of the *SURF6* coronary artery eQTL that displayed significant colocalization for the locus. The points representing different variants in the GWAS locus are color coded based on their LD (r^2) relative to the lead GWAS variant (chr9_133255801_C_T_b38) (**a**) or the eVariant (chr9_133226170_T_C_b38) with the most significant eCAVIAR colocalization posterior probability (CLPP=0.014) for the locus (**b**). It can be observed that the eQTL p-value of the lead GWAS variant is not significant, and the eQTL and GWAS p-values of the eVariant with the highest CLPP are only nominally significant. **c-f** LocusCompare plots of -$\log_{10}$ P-value of the IOP GWAS as a function of -$\log_{10}$ P-value of an *RBK4* coronary artery eQTL (**c,d**) or *B4GALNT2* colon transverse sQTL (**e,f**) which displayed significant colocalization with the corresponding locus. Points are color coded based on their LD (r^2) relative to the lead GWAS variant (**c,e**) or the eVariant with the highest *enloc* regional colocalization probability (RCP) (**d,f**) represented by a purple diamond. These two examples demonstrate an eQTL and sQTL that colocalize with an IOP GWAS locus with high confidence (RCP>0.85), though the variant with the highest RCP has a non-significant GWAS p-value.

Supplementary Figure 4. Correlation of number of target genes of significantly colocalizing e/sQTLs with POAG and IOP GWAS loci with tissue sample size

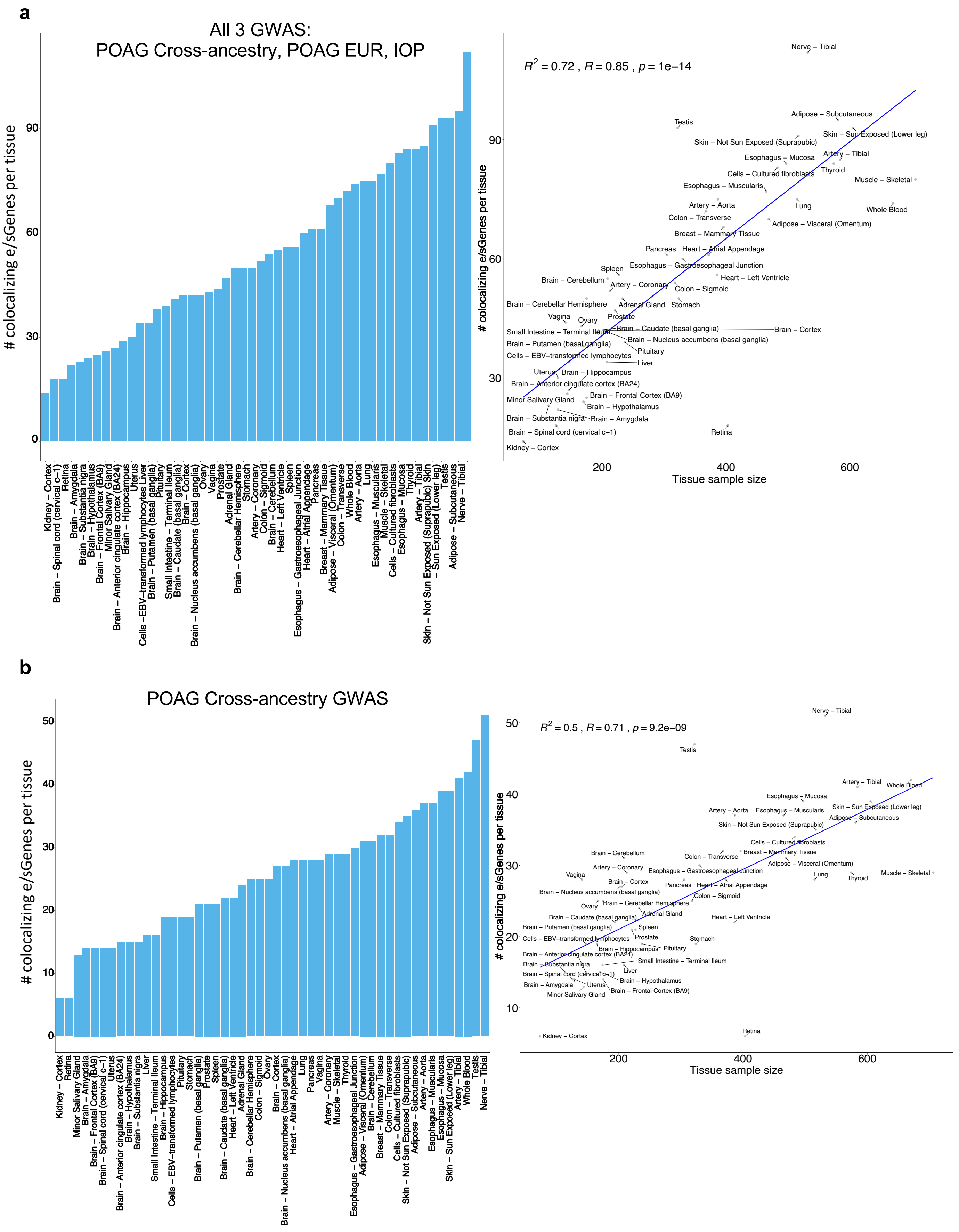

c

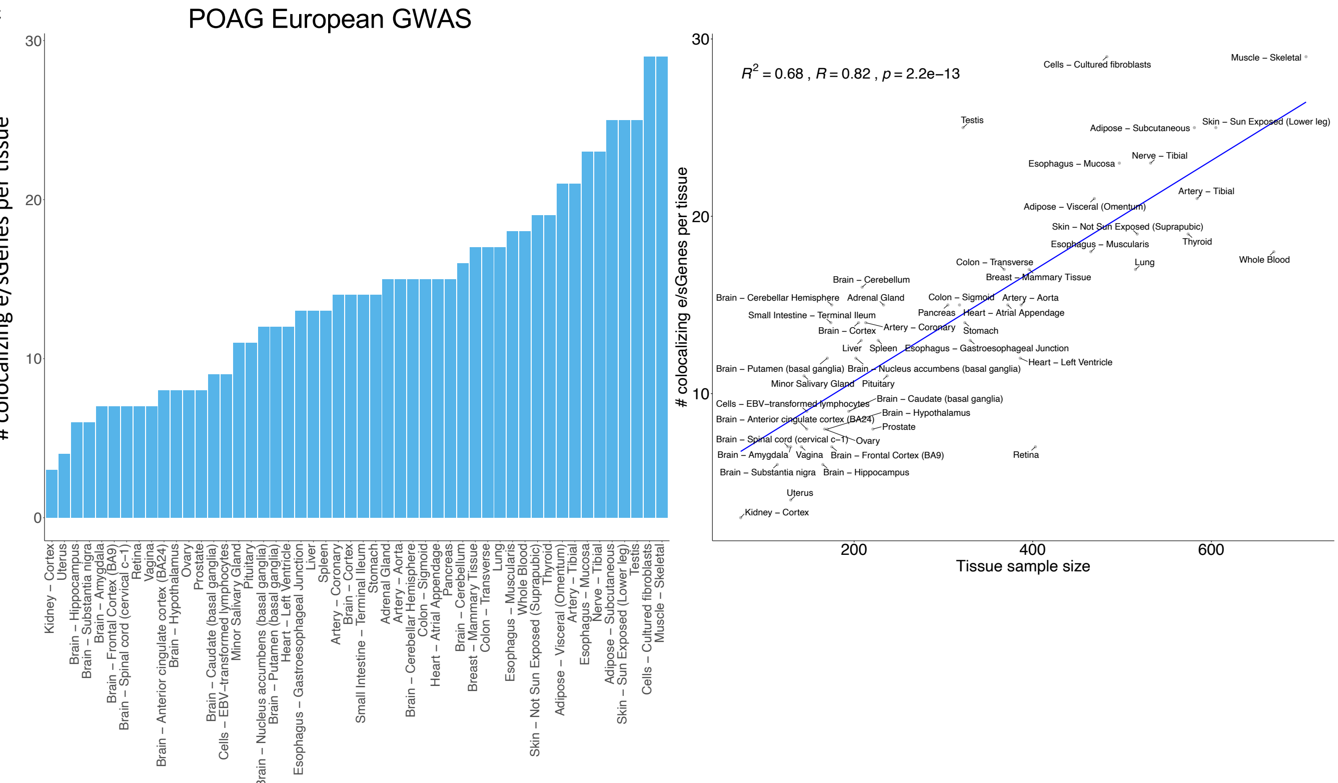

d

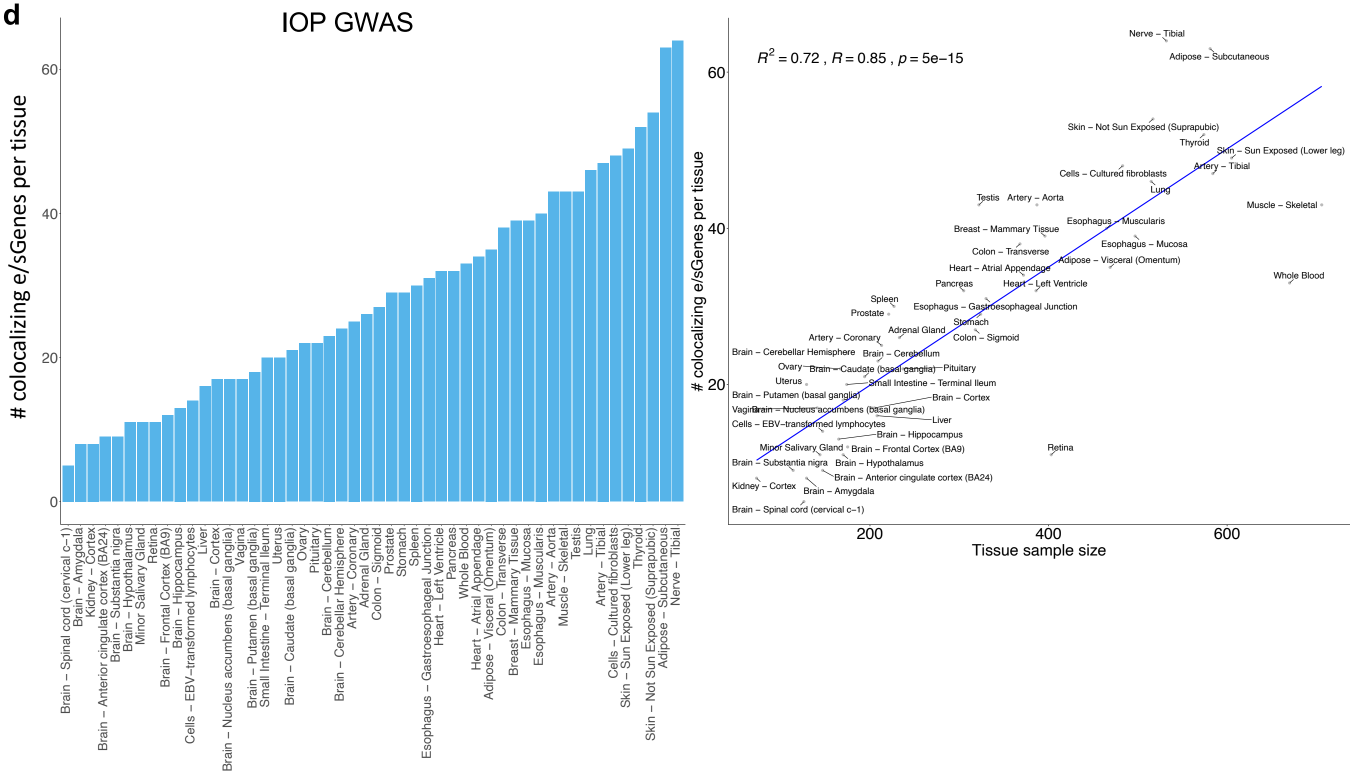

Supplementary Figure 4: Correlation of number of target genes of significantly colocalizing e/sQTLs with POAG and IOP GWAS loci with tissue sample size. Left panel: histogram of unique number of colocalizing e/sGenes per GTEx and retina tissue based on eCAVIAR (CLPP>0.01) and/or *enloc* (RCP>0.1). Right panel: scatter plot of number of colocalizing e/sGenes per tissue versus tissue sample size. Determination of coefficient (R^2), Pearson correlation coefficient (R), and p-value are shown. Blue line is a least squares fitted line. These were generated for the unique union of colocalizing e/sGenes with the POAG cross-ancestry, POAG European ancestry subset, and IOP GWAS loci (**a**), or only the POAG cross-ancestry **(b)**, POAG European subset (**c**), and IOP (**d**) GWAS loci.

Supplementary Figure 5. Colocalizing e/sQTLs with top POAG European and IOP GWAS loci

a

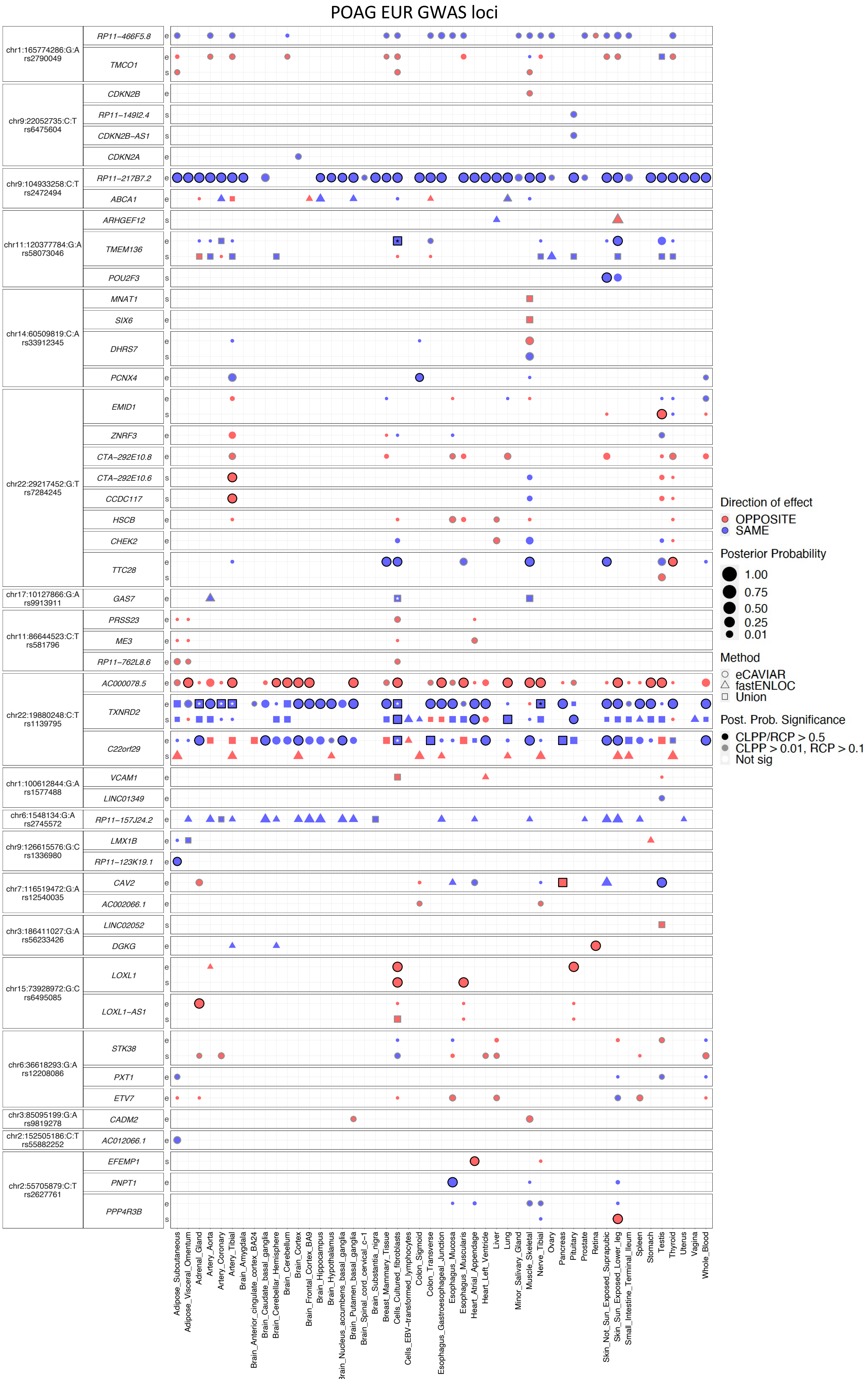

b

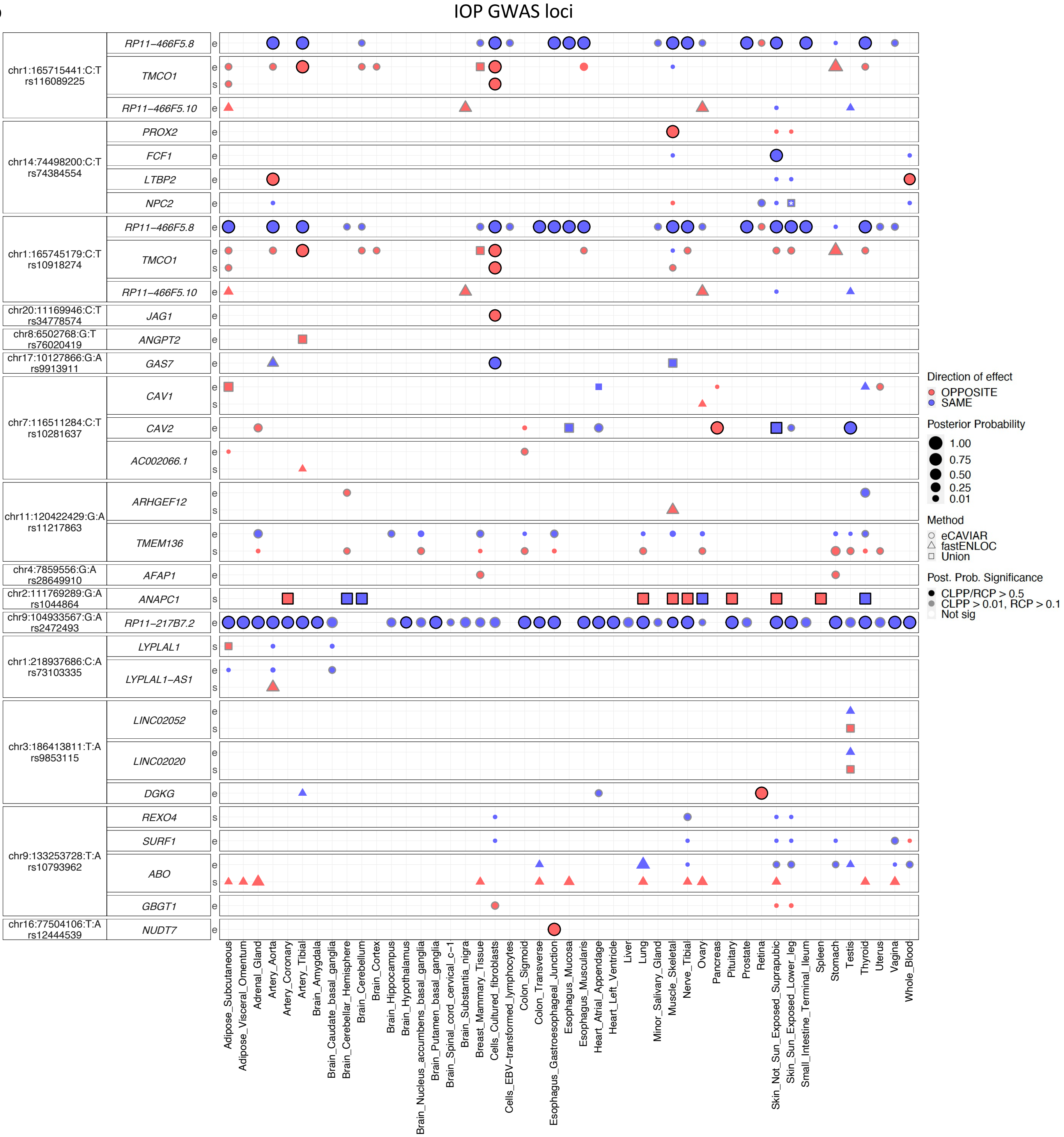

Supplementary Figure 5. Colocalizing e/sQTLs with top POAG European GWAS and IOP GWAS loci. a-b, Colocalization results for the top POAG EUR GWAS (**a**) and IOP EUR GWAS loci (**b**). Only genes with at least one significant colocalization result are shown for e/sQTLs tested across 49 GTEx tissues and peripheral retina per locus. GWAS loci were ordered by absolute value of their effect size in descending order, and within each locus, genes were ordered based on their chromosome position. Bubble size is proportional to the maximum colocalization posterior probability of all e/sVariants tested for the given gene, QTL type and tissue combination. Points are color-coded by direction of effect (blue if increased expression or splicing increases POAG risk or IOP levels or vice versa; red if increased expression or splicing decreases POAG risk or IOP levels or vice versa). Shape of points indicates colocalization method used: circle (eCAVIAR), triangle (*enloc*), and square (tested in both methods; results shown for method with maximum posterior probability). Grey or black border denotes variant-gene-tissue-QTL combination that passed QC filtering (Methods) and a colocalization posterior probability cutoff above 0.01/0.1 (CLPP/RCP) or 0.5 (higher confidence), respectively. White or black asterisk in the square indicates whether the second method tested passed a posterior probability cutoff 0.01/0.1 (CLPP/RCP) or 0.5, respectively.

Supplementary Figure 6. *TMCO1* e/sQTLs colocalizing with POAG and IOP GWAS loci.

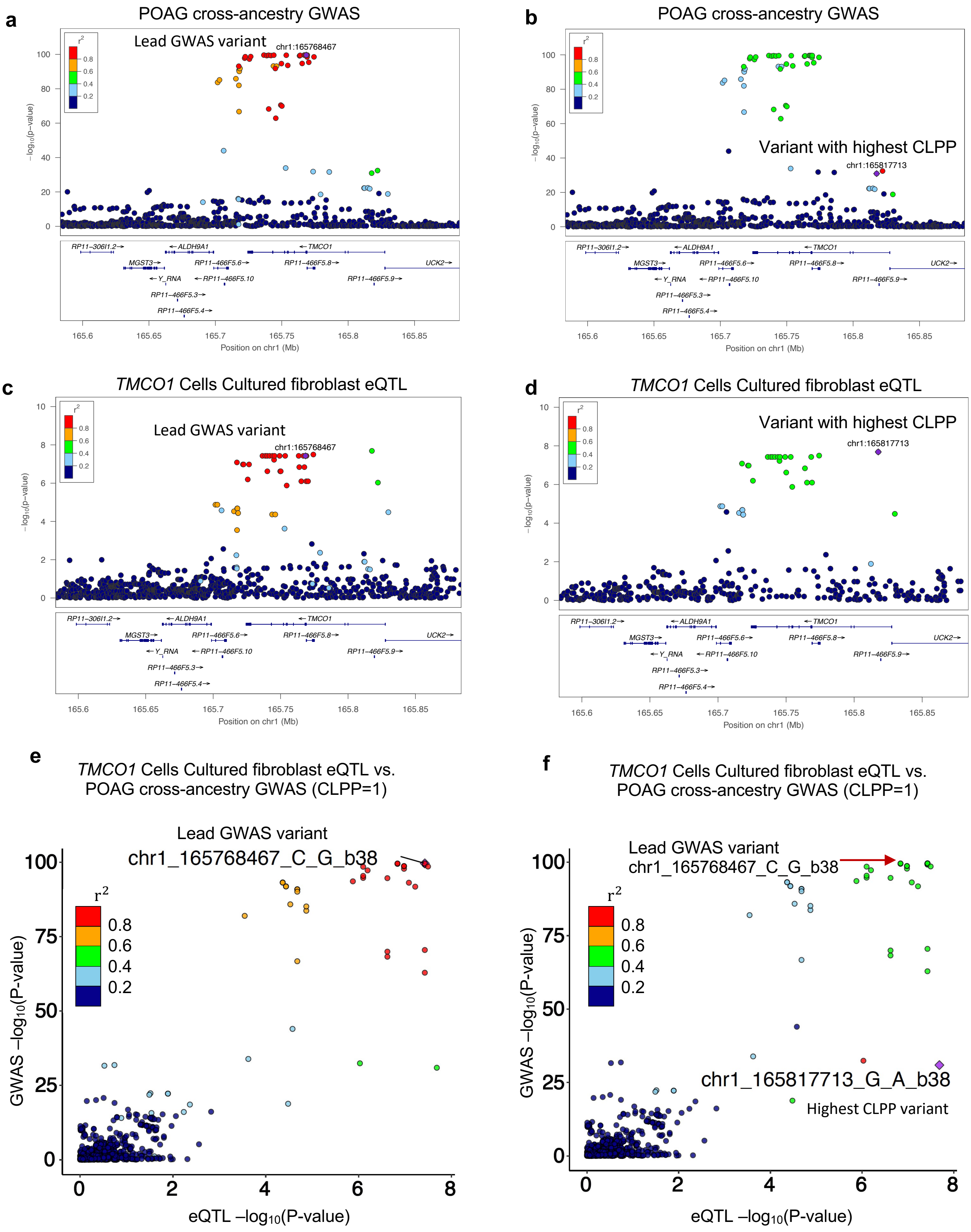

Supplementary Figure 6. ***TMCO1*** **e/sQTLs colocalizing with POAG and IOP GWAS loci.**

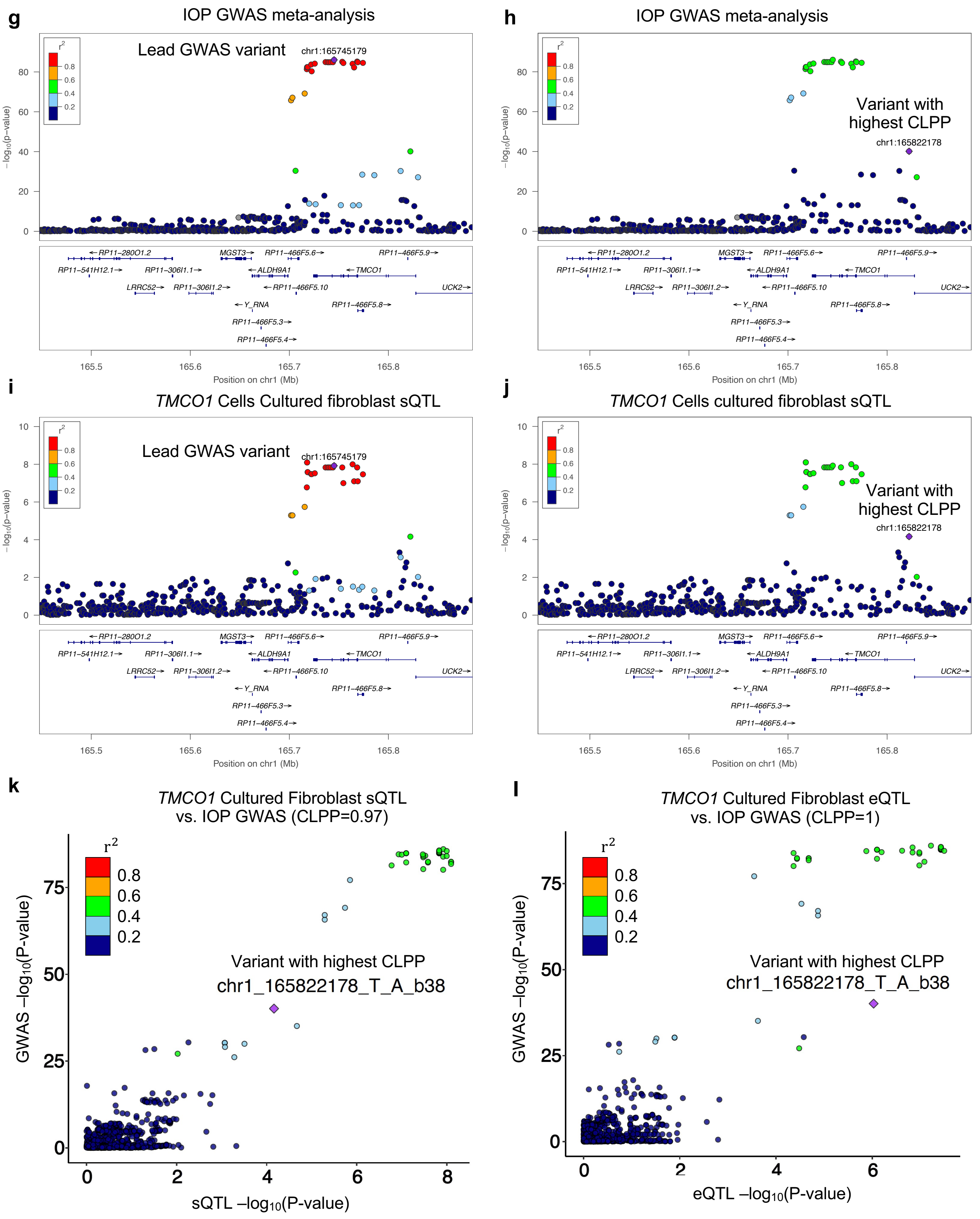

Supplementary Figure 6. *TMCO1* e/sQTLs colocalizing with POAG and IOP GWAS loci. a-d, LocusZoom plots for the POAG cross-ancestry GWAS p-values (**a,b**) and *TMCO1* Cell Cultured fibroblast eQTL p-values (**c,d**) on $-\log_{10}$ scale for the POAG locus rs2790053 (chr1_165768467_C_G_b38). Variants are color-coded by LD (r^2) relative to the lead GWAS variant (**a,c**) or the eVariant with the highest colocalization posterior probability (**b,d**). **e-f**, LocusCompare plots for *TMCO1* Cells Cultured fibroblast eQTL p-values relative to POAG cross ancestry GWAS p-values on $-\log_{10}$ scale for all variants in the GWAS locus LD interval. Variants are color-coded by LD relative to the lead GWAS variant chr1_165768467_C_G_b38 (**e**) or the variant with highest colocalization posterior probability (chr1_165817713_G_A_b38; CLPP=1) (**f**). **g-j**, LocusZoom plots for the IOP GWAS p-values (**g,h**) and *TMCO1* Cells Cultured fibroblast sQTL (intron excision cluster: chr1:165768269-165768482) p-values (**i,j**) on $-\log_{10}$ scale for the IOP locus rs10918274 (chr1_165745179_T_C_b38). Variants are color-coded by LD (r^2) relative to the lead GWAS variant chr1_165745179_T_C_b38 (**g,i**) or the variant with the highest CLPP (chr1_165822178_T_A_b38; CLPP=0.97) (**h,j**). **k-l,** LocusCompare plots for *TMCO1* Cells Cultured fibroblast sQTL (**k**) or eQTL (**l**) p-values relative to IOP GWAS p-values on $-\log_{10}$ scale for all variants in the locus LD interval. Variants are color-coded by LD (r^2) relative to the variant with the highest colocalization posterior probability for the *TMCO1* (**k**) sQTL (CLPP=0.97) or (**l**) eQTL (CLPP=1) (chr1_165822178_T_A_b38).

Supplementary Figure 7. *TMCO1* and *TMCO1-AS1* single-nucleus expression in anterior and posterior eye tissues.

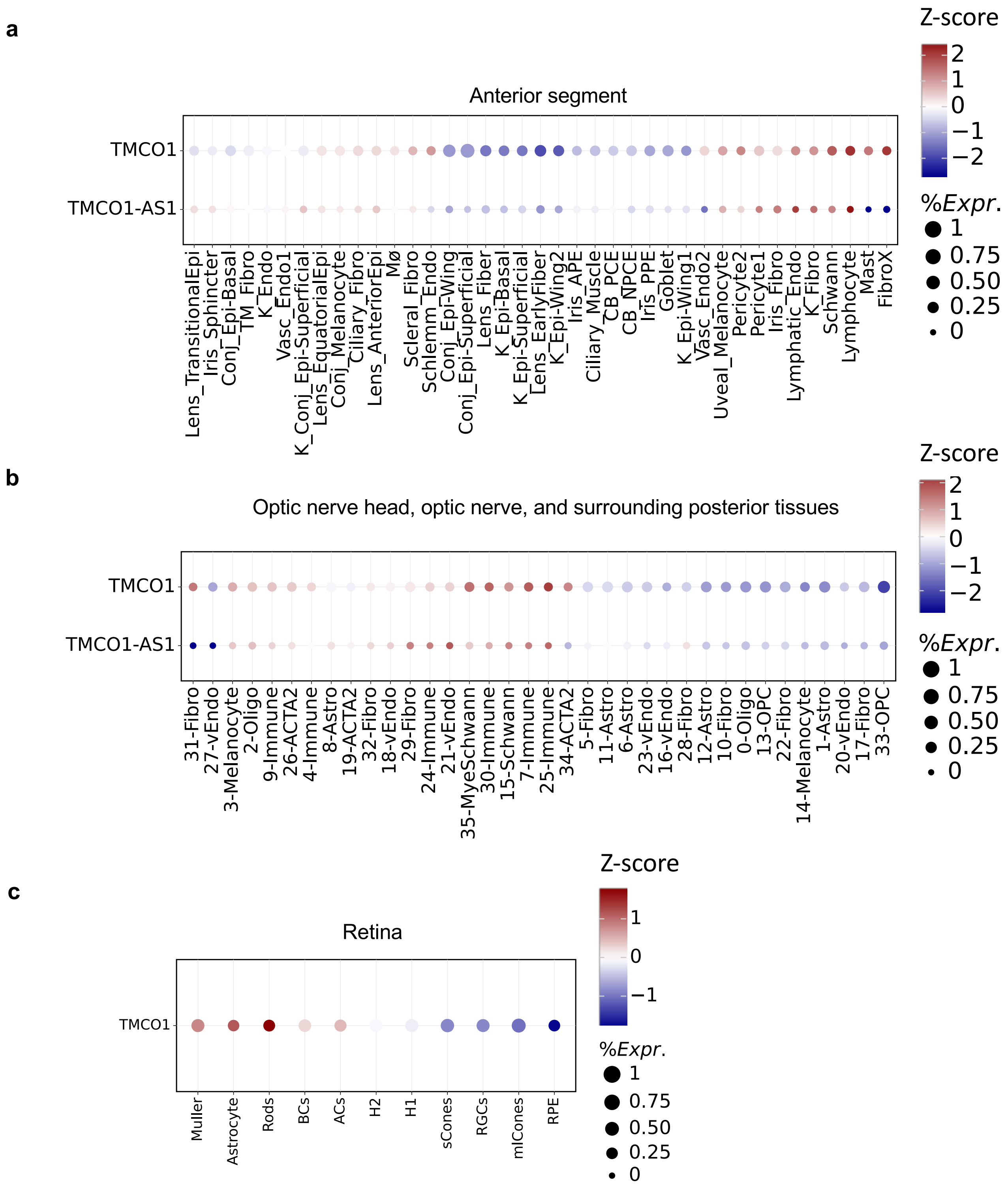

Supplementary Figure 7. *TMCO1* and *TMCO1-AS1* single-nucleus expression in anterior and posterior eye tissues. a-d, Bubble maps displaying the average expression of *TMCO1* and its anti-sense, *TMCO1-AS1* (*RP11-466F5.8*) across all cell types in six tissues in the anterior segment (**a**), optic nerve head and surrounding posterior tissues (**b**), and retina (**c**). *TMCO1-AS1* was not expressed in retina. The colorbar represents z-scores computed by comparing each gene's average expression in a given cell type to its per cell type average expression across all types divided by the standard deviation of all cell type expression averages. Bubble size is proportional to the percentage of cells expressing the given gene (log(TPK+1)). Cell type abbreviations are described in Supplementary Data 35.

Supplementary Figure 8. Example of colocalizing e/sQTLs with IOP GWAS loci.

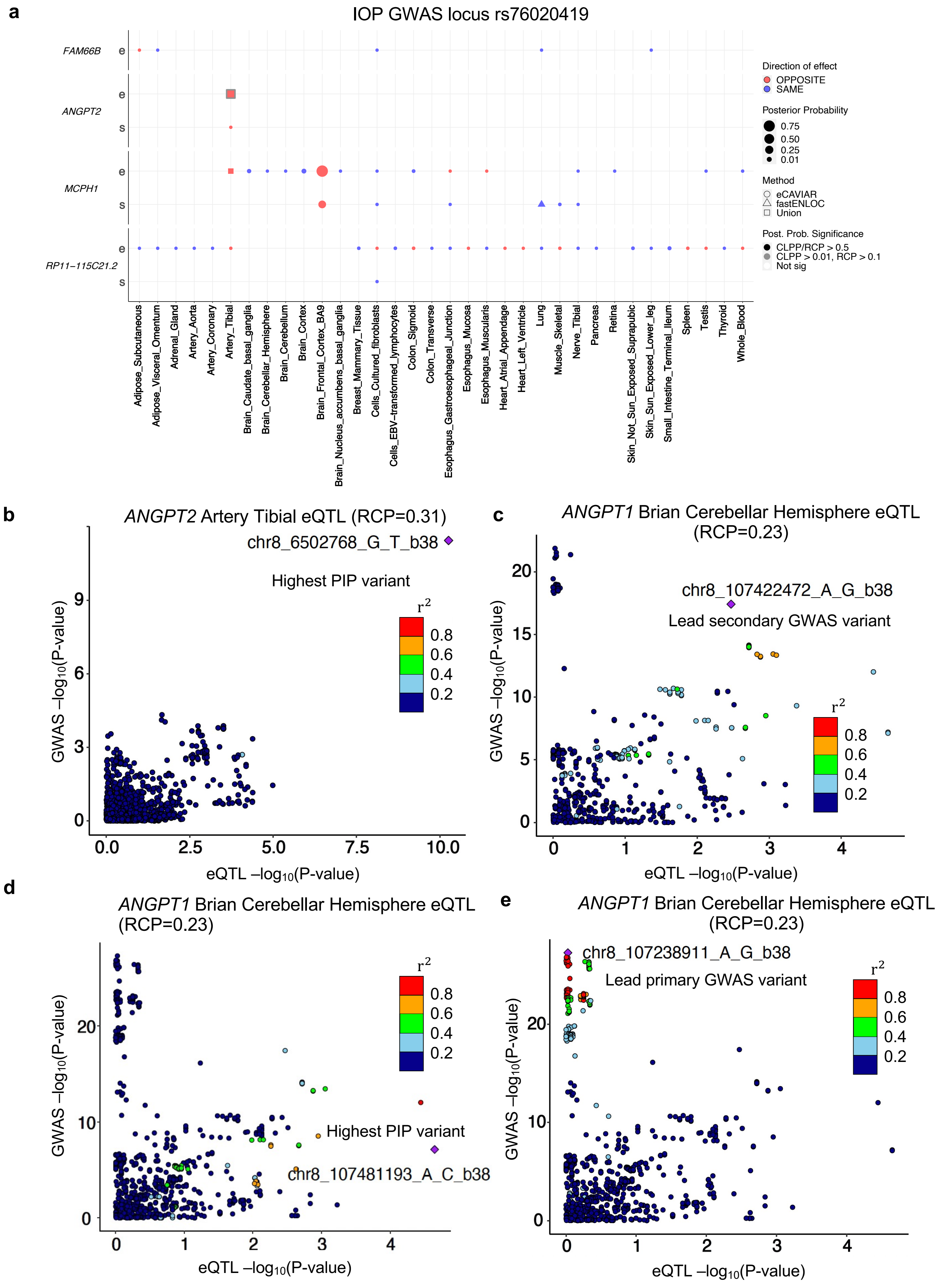

Supplementary Figure 8. Example of colocalizing e/sQTLs with IOP GWAS loci. a, Colocalization results for all e/sGenes tested in the IOP GWAS locus rs76020419 locus LD interval that had at least one significant eQTL or sQTL result with eCAVIAR or *enloc* across 49 GTEx tissues and peripheral retina. Genes were ordered by chromosome position. Size of points is proportional to the maximum colocalization posterior probability of all e/sVariants tested for the given gene, QTL type and tissue combination. Points are color-coded by direction of effect (blue if increased expression or splicing increases IOP levels or vice versa; red if increased expression or splicing decreases IOP levels or vice versa). Shape of points indicates colocalization method used: circle (eCAVIAR), triangle (*enloc*), and square (tested in both methods; results shown for method with maximum posterior probability). Grey or black border denote variant-gene-tissue-QTL combination that passed QC filtering (Methods) and a colocalization posterior probability cutoff above 0.01/0.1 (CLPP/RCP) or 0.5, respectively. White or black asterisk in the square indicates whether the second method tested passed a posterior probability cutoff of 0.01/0.1 (CLPP/RCP) or 0.5, respectively. **b-e,** LocusCompare plot of -$\log_{10}$(P-value) of the IOP GWAS relative to the -$\log_{10}$(P-value) of artery tibial eQTL acting on *ANGPT2* (**b**) or brian cerebellar hemisphere eQTL acting on *ANGPT1* (**c-e**). Points are color-coded based on LD (r^2) relative to the lead GWAS variant (**c,e**) or the variant with highest *enloc* posterior inclusion probability (PIP) (**b,d**). Interestingly, the eQTL acting on *ANGPT1 in* brain cerebellar hemisphere significantly colocalized (RCP=0.23) with the secondary independent IOP GWAS variant rs4496939 (chr8:107422472:A:G, beta = -0.118, P=2.7x10^{-18}) (**c,d**), and not with the stronger independent IOP signal rs2022945 (chr8:107238911:G:A, beta = -0.213, P=1.1x10^{-28}) (**e**) of the three independent signals in the locus.

Supplementary Figure 9. *GAS7* eQTL colocalizing with POAG and IOP GWAS loci.

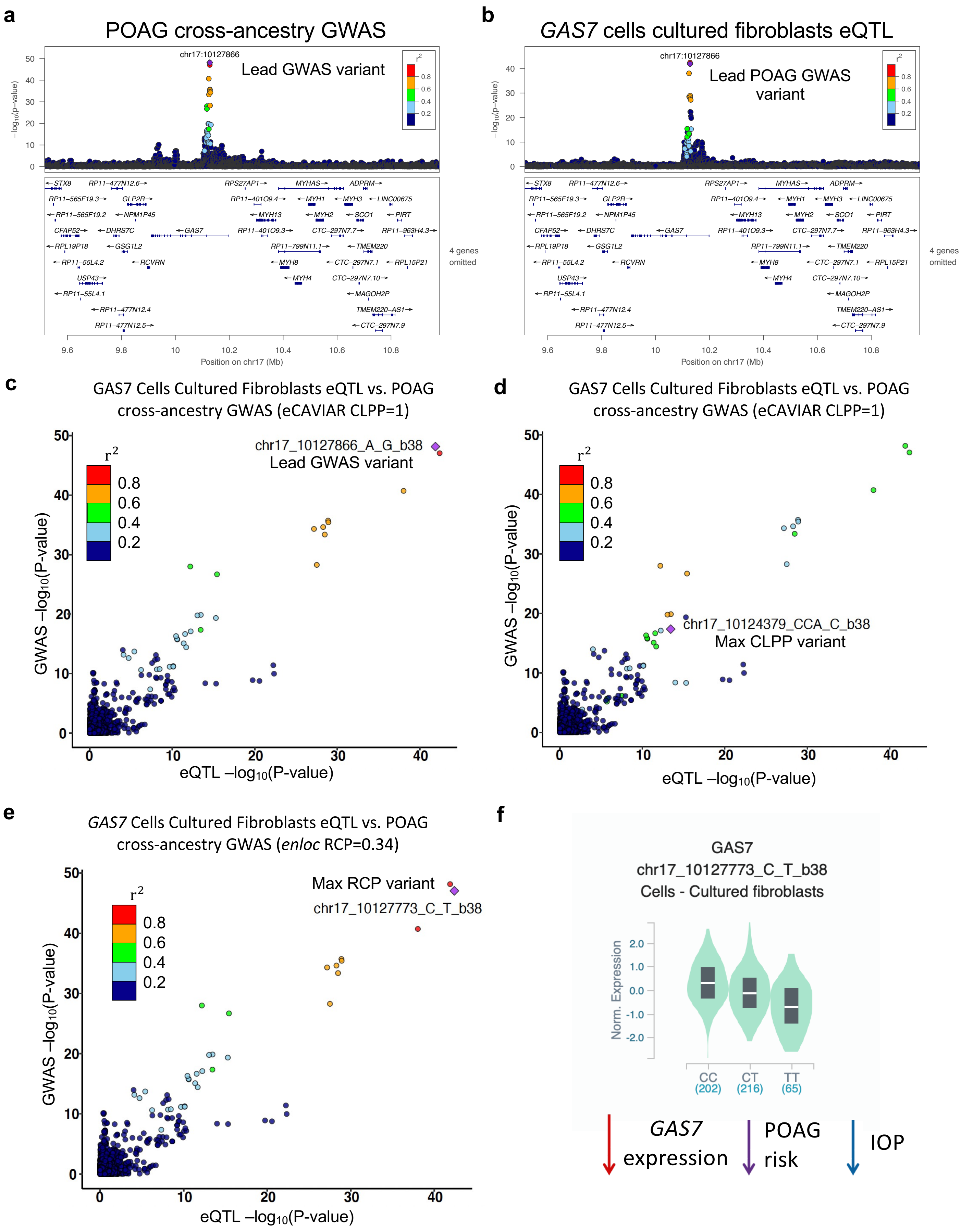

g

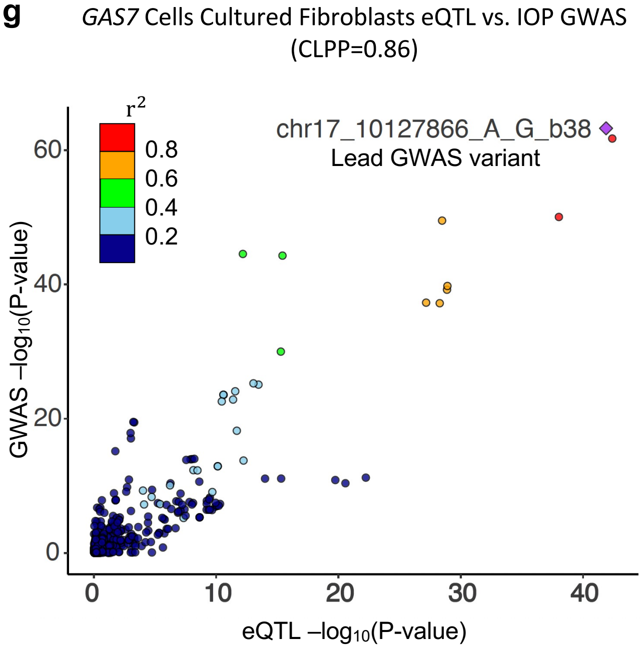

h

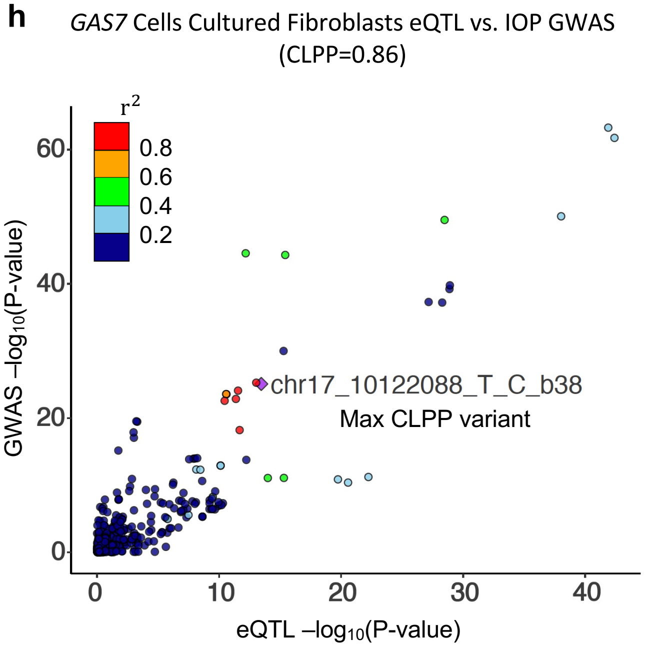

Supplementary Figure 9. *GAS7* eQTL colocalizing with POAG and IOP GWAS loci. a-d, LocusZoom plots for the POAG cross-ancestry GWAS p-values (**a**) and *GAS7* Cells Cultured fibroblast eQTL (**b**) -log$_{10}$(P-values) for the POAG cross-ancestry locus with lead variant rs9913911 (chr17_10127866_A_G_b38). Variants are color-coded by LD (r^2) relative to the lead GWAS variant. **c-e,g**, LocusCompare plots for -log$_{10}$(P-value) of POAG cross ancestry GWAS (**c-e**) or IOP GWAS (**g**) compared to *GAS7* Cells Cultured fibroblast eQTL -log$_{10}$(P-value) for all variants in the GWAS locus LD interval including eVariants that significantly colocalized with POAG and IOP. Variants are color-coded by LD (r^2) relative to the lead GWAS variant (**c,g**) or the variant with highest colocalization posterior probability based on eCAVIAR (**d,h**) or enloc (**e**). **f,** Violin plot of normalized *GAS7* expression values in Cells Cultured fibroblasts as a function of the genotype of the eVariant chr17_10127773_C_T_b38 (rs12150284) that had the highest RCP value, taken from the GTEx portal (https://gtexportal.org). The effect size of this eVariant relative to the alternative allele (eQTL *beta* = -0.44) is in the same direction relative to POAG risk (POAG cross-ancestry GWAS beta = -0.14), suggesting that decreased *GAS7* expression is protective for POAG.

Supplementary Figure 10. *ABO* eQTL colocalizing with POAG and IOP GWAS loci

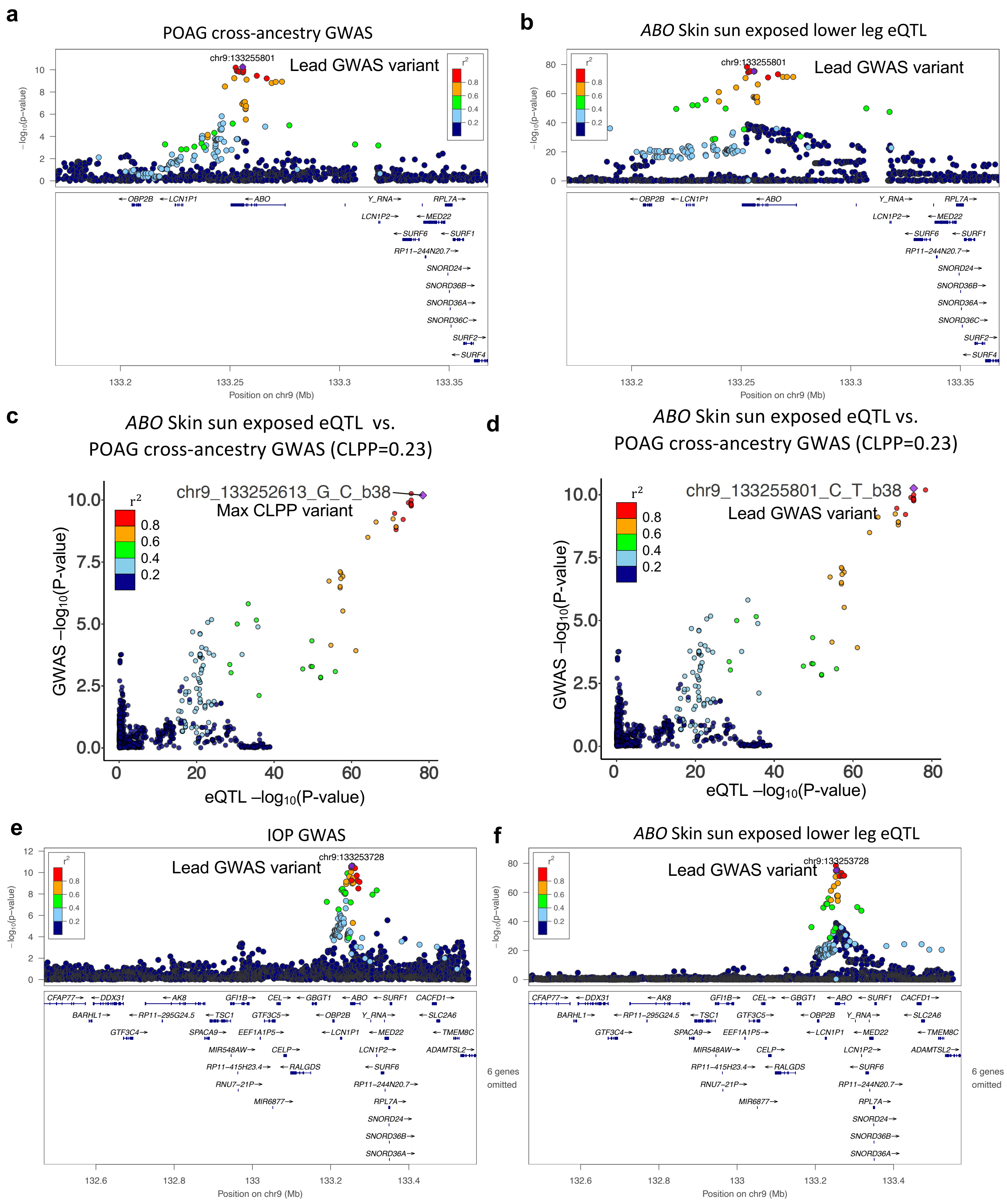

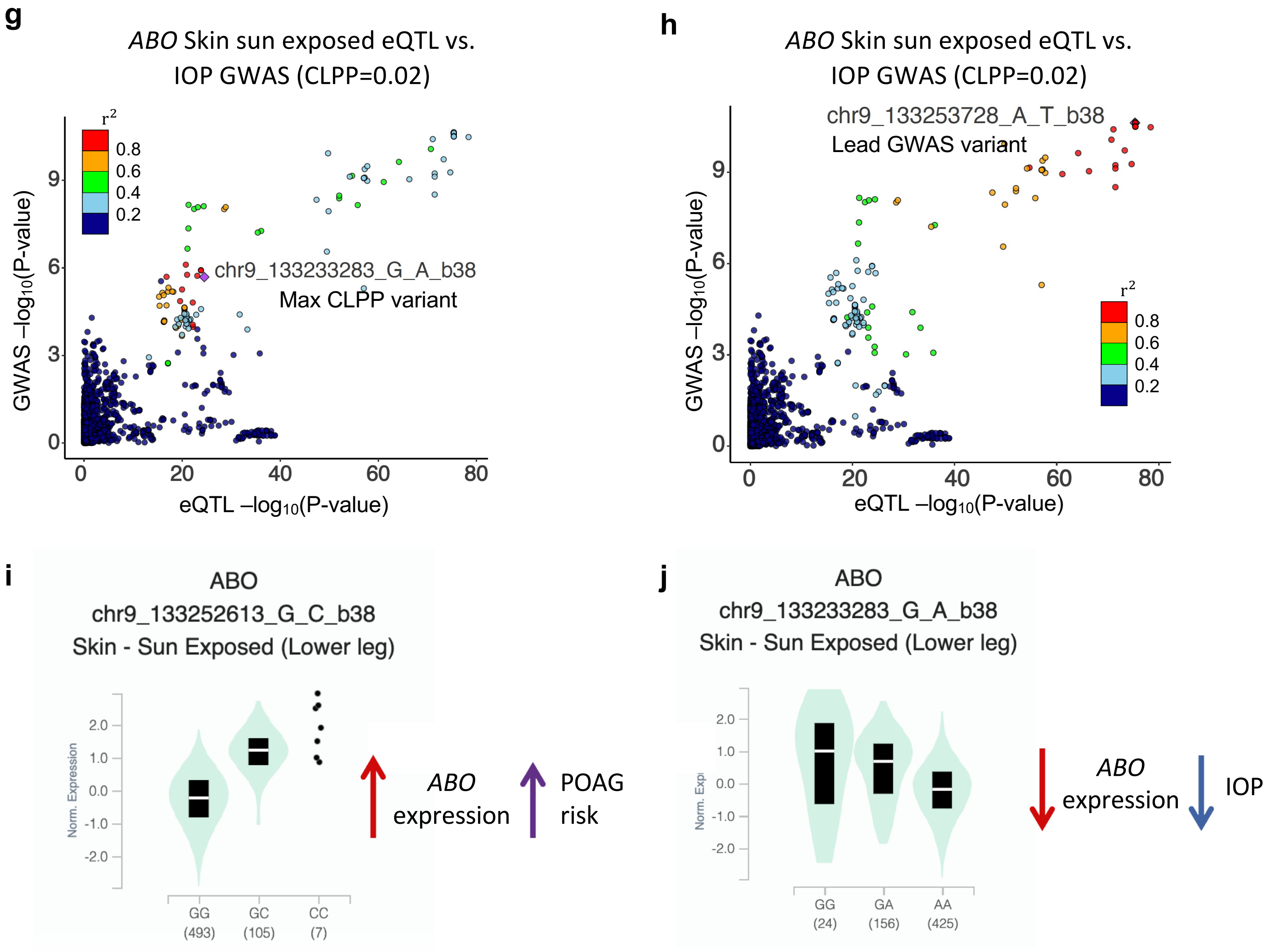

Supplementary Figure 10. *ABO* eQTL colocalizing with POAG and IOP GWAS loci. a-b, LocusZoom plots of -log$_{10}$(P-value) of the POAG cross-ancestry GWAS meta-analysis (**a**) and *ABO* Skin sun exposed (lower leg) eQTL (**b**) for POAG locus rs8176749 (chr9:133255801_C_T_b38). Variants are color-coded by LD (r^2) relative to the lead GWAS variant (chr9_133255801_C_T_b38). **c-d,** LocusCompare plots of -log$_{10}$(P-value) of the POAG cross-ancestry GWAS meta-analysis compared to the *ABO* Skin sun exposed lower leg eQTL that significantly colocalized with POAG GWAS association in locus rs8176749. Points are color-coded based on their LD (r^2) relative to the variant with the highest colocalization posterior probability (chr9_133252613_G_C_b38; CLPP=0.23) (**c**) or the lead GWAS variant (chr9_133255801_C_T) (**d**). **e-f**, LocusZoom plots of -log$_{10}$(P-value) of IOP GWAS meta-analysis (**e**) and *ABO* Skin sun exposed lower leg eQTL (**f**) for the IOP locus rs10793962 (chr9_133253728_T_A_b38). Variants are color-coded by LD (r^2) relative to the lead GWAS variant (chr9_133253728_T_A_b38). **g-h,** LocusCompare plots of -log$_{10}$(P-value) of IOP GWAS meta-analysis compared to *ABO* Skin sun exposed lower leg eQTL that significantly colocalized with the IOP GWAS locus rs10793962. Points are color-coded based on their LD (r^2) relative to the variant with the highest colocalization posterior probability (chr9_133233283_G_A_b38; CLPP=0.02) (**f**) or the lead GWAS variant (chr9_133253728_T_A_b38) (**h**). **i-j,** Violin plot of normalized *ABO* expression values in Skin sun exposed (lower leg) as a function of the genotype of the eVariant chr9_133252613_G_C_b38 or chr9_133233283_G_A_b38 that displayed the highest colocalization posterior probability for POAG cross-ancestry (**i**) and IOP (**j**) GWAS, respectively, taken from the GTEx portal (https://gtexportal.org). The effect size of this *ABO* eVariant relative to the alternative allele, *beta* = 1.35 and -0.67 for POAG (**i**) and IOP (**j**), respectively is in the same direction relative to POAG risk (POAG cross-ancestry GWAS beta = 0.096) or IOP level (GWAS beta = -0.094), respectively, suggesting that decreased *ABO* expression may be protective for POAG.

Supplementary Figure 11. e/sQTL colocalization results for *CDKN2A/B* POAG cross-ancestry GWAS locus

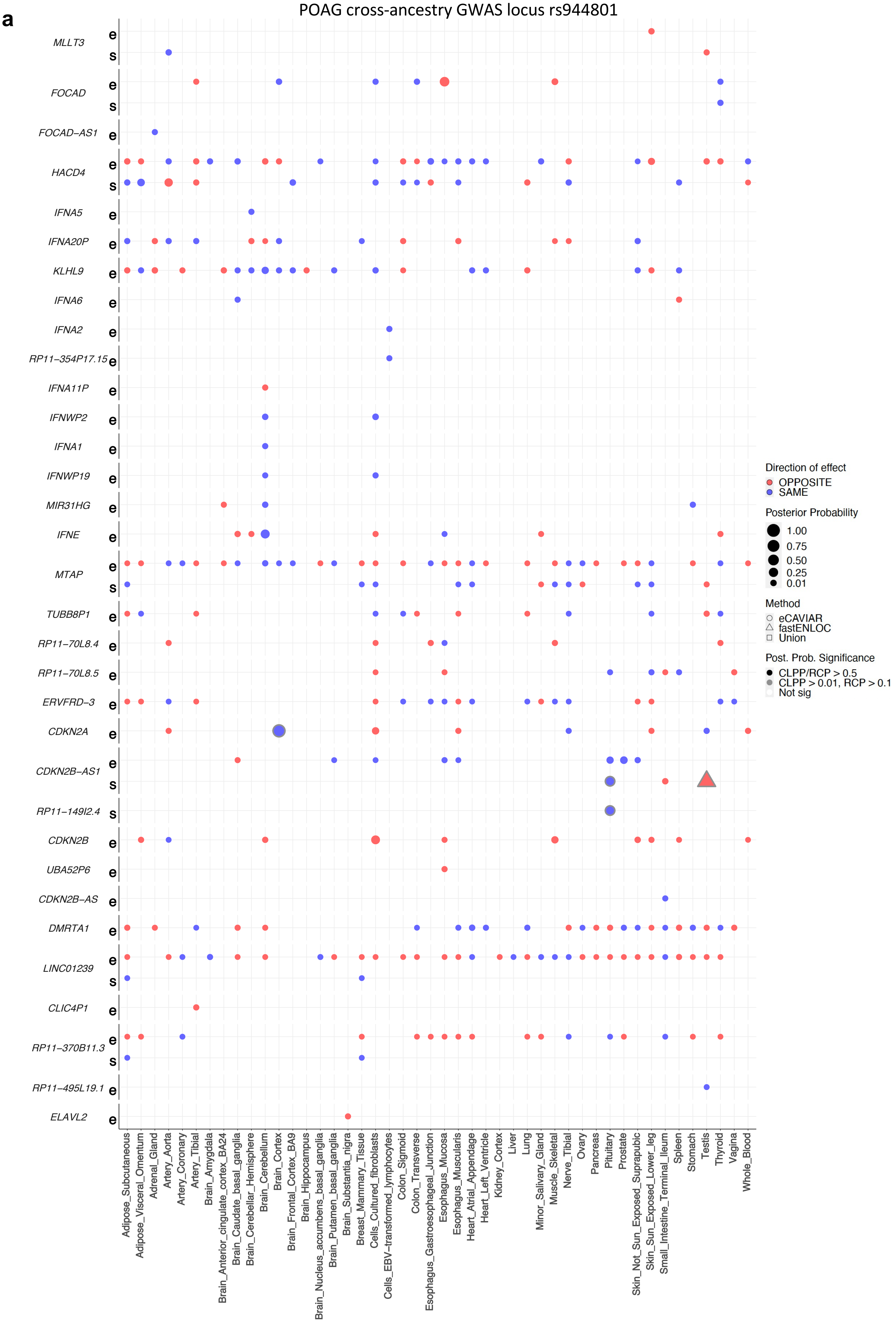

Supplementary Figure 11. e/sQTL colocalization results for *CDKN2A/B* POAG cross-ancestry GWAS locus

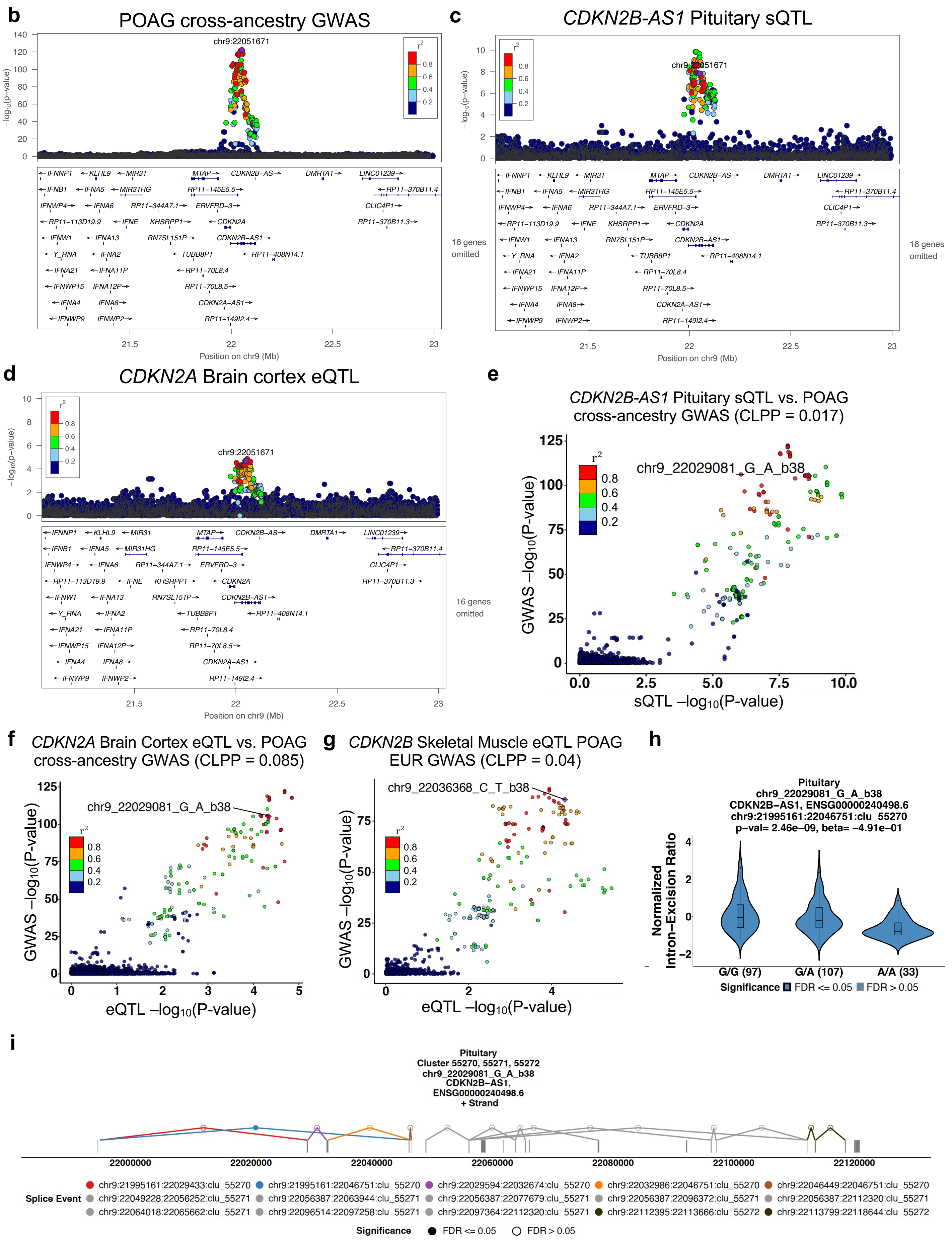

Supplementary Figure 11. e/sQTL colocalization results for *CDKN2A/B* POAG cross-ancestry locus. a, Colocalization results for all genes tested in the POAG cross-ancestry GWAS locus rs944801 (chr9_22051671_G_C_b38) LD interval that had at least one significant eQTL or sQTL result across 49 GTEx tissues and peripheral retina. Genes were ordered by chromosome position. Size of points is proportional to the maximum colocalization posterior probability of all e/sVariants tested for the given gene, QTL type and tissue combination. Points are color-coded by direction of effect (blue if increased expression or splicing increases POAG risk or vice versa; red if increased expression or splicing decreases POAG risk or vice versa). Shape of points indicates colocalization method used: circle (eCAVIAR), triangle (*enloc*), and square (tested in both methods; results shown for method with maximum posterior probability). Grey or black border denote variant-gene-tissue-QTL combination that passed QC filtering (see Methods) and a colocalization posterior probability cutoff above 0.01/0.1 (CLPP/RCP) or 0.5, respectively. White or black asterisk in the square indicates whether the second method tested passed a posterior probability cutoff of 0.01/0.1 (CLPP/RCP) or 0.5, respectively. **b-d**, LocusZoom plots for the POAG GWAS locus rs944801 showing the $-\log_{10}$(P-value) of the POAG cross-ancestry GWAS (b), *CDKN2B-AS1* pituitary sQTL (c), and *CDKN2A* Brain cortex eQTL (**d**) in the GWAS locus LD interval. Points are color-coded by LD relative to the lead GWAS variant (chr9_22051671_G_C_b38). **e-g,** LocusCompare plots of $-\log_{10}$(P-value) of the POAG cross-ancestry GWAS meta-analysis relative to the $-\log_{10}$(P-value) of the *CDKN2B-AS1* pituitary sQTL (**e**) or *CDKN2A* Brain cortex eQTL (**f**), and of the POAG European subset GWAS versus the *CDKN2B* Skeletal muscle eQTL (**g**), all of which showed significant colocalization results. Points are color-coded based on LD (r^2) relative to the e/sVariant with the highest eCAVIAR colocalization posterior probability (CLPP). **h,** Violin plot of normalized intron-excision ratio for chr9:21995161-22046751 computed with Leafcutter for *CDKN2B-AS1* in Pituitary as a function of the genotype of the sVariant rs679038 (chr9_22029081_G_A_b38) that displayed the highest CLPP (0.017) for this sQTL and POAG cross-ancestry GWAS signal in locus rs944801 (chr9_22051671_G_C_b38). This sVariant also significantly colocalized with the POAG European subset GWAS locus rs6475604 (chr9_22052735_T_C_b38) (CLPP=0.11). The effect size of the sQTL relative to the alternative allele (*beta* = -0.49) is in same direction relative to POAG risk (POAG cross-ancestry GWAS beta = -0.22; POAG European GWAS beta = -0.24), suggesting that decrease in splicing between chr9:21995161-22046751 may decrease POAG risk. **i,** Gene model for *CDKN2B-AS1* in GTEx Pituitary showing all splicing events detected with Leafcutter for *CDKN2B-AS1* in this tissue (positive strand). Intron excision cluster 55270 is colored because it has a significant splicing event, chr9:21995161-22046751 (color Blue), which skips exon 2 and 3 that is associated with the sVariant rs679038 (chr9_22029081_G_A_b38) that colocalized with the POAG cross-ancestry GWAS locus rs944801 (chr9_22051671_G_C_b38), as well as with the POAG European GWAS locus rs6475604 (chr9_22052735_T_C_b38). Retention of exon 2 and 3 in *CDKN2B-AS1* is protective for POAG (**h**).

Supplementary Figure 12. *EFEMP1* sQTL colocalizing with POAG GWAS locus.

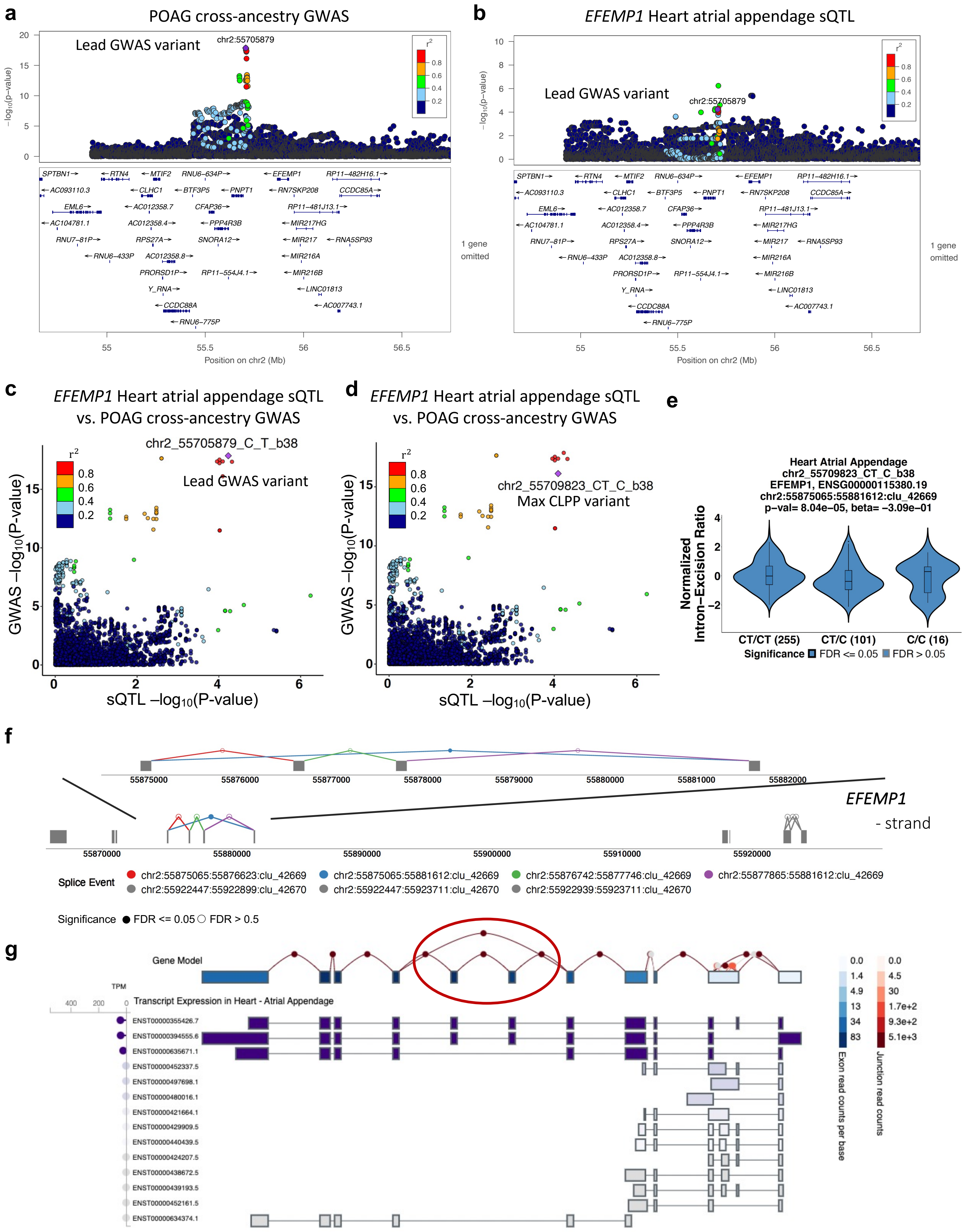

Supplementary Figure 12. *EFEMP1* sQTL colocalizing with POAG GWAS locus. a-b, LocusZoom plots of $-\log_{10}$(P-value) of POAG cross-ancestry GWAS meta-analysis (**a**) and *EFEMP1* Heart atrial appendage sQTL (**b**) that colocalized with the POAG cross-ancestry association in locus rs2627761 (chr2_55705879_C_T_b38). Variants are color-coded by LD (r^2) relative to the lead GWAS variant (chr2_55705879_C_T_b38). **c-d,** LocusCompare plots of $-\log_{10}$(P-value) of the POAG cross-ancestry GWAS meta-analysis compared to the *EFEMP1* Heart atrial appendage sQTL $-\log_{10}$(P-value). Points are color-coded based on their LD (r^2) relative to the lead GWAS variant (chr2_55705879_C_T_b38) (**c**) or the sVariant with the highest colocalization posterior probability (chr2_55709823_CT_C_b38**;** CLPP=0.51) (**d**). **e**, Violin plot of normalized intron-excision ratio for chr2:55875065-55881612 computed with Leafcutter for *EFEMP1* in Heart Atrial Appendage as a function of the genotype of the sVariant with the highest CLPP for this sQTL (rs35017406, chr2_55709823_CT_C_b38) and POAG GWAS locus rs2627761 (CLPP=0.51). The effect size of the sQTL relative to the alternative allele (*beta* = -0.31) is in opposite direction relative to the POAG cross-ancestry association (*beta* = 0.10), suggesting that increase in splicing between chr2:55875065-55881612 decreases risk of POAG. **f,** Gene model for *EFEMP1* in GTEx Heart Atrial Appendage on the negative strand, showing all splicing events in the gene (open circles), and zooming in on the splice event chr2:55875065-55881612 (cluster:42669) from exon 5 to exon 8 (blue line and closed circle) that has an sQTL (chr2_55709823_CT_C_b38) that significantly colocalized with POAG cross-ancestry association in the locus. From panel **e** and Supplementary Data 2, it can be understood that skipping of exons 6 and 7 of *EFEMP1* decreases risk of POAG. **g,** *EFEMP1* gene model and transcripts expressed in GTEx Heart Atrial Appendage taken from the GTEx portal (URLs). In the gene model, exon boxes are color-coded by exon read counts per base (blue) and lines connecting exons by exon-exon junction read counts (red). All splicing events observed in the tissue are shown, including the alternative splicing between exon 5 and exon 8 in *EFEMP1* whose genetic regulation colocalized with POAG (**d**). Below the gene model, transcripts expressed in Heart Atrial Appendage in Transcripts per Million (TPM), computed with RSEM, are shown in descending order.

Supplementary Figure 13. Top GWAS POAG locus with *MYOC* mutation colocalizes with *PIGC* sQTL.

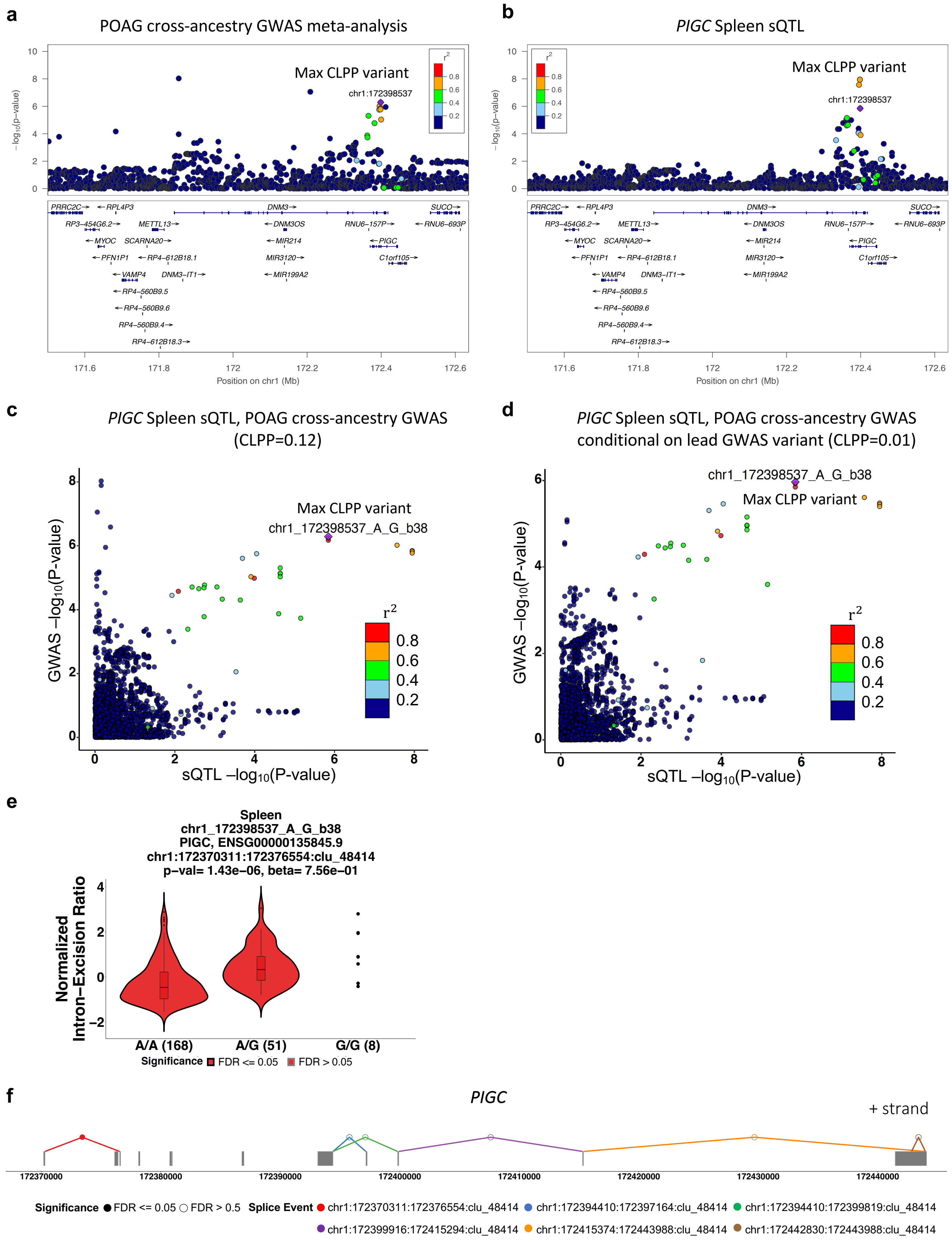

Supplementary Figure 13. Top GWAS POAG locus with *MYOC* mutation colocalizes with *PIGC* sQTL. a-b, LocusZoom plots of -$\log_{10}$(P-value) of POAG cross-ancestry GWAS meta-analysis (**a**) and *PIGC* Spleen sQTL (**b**) that colocalized with the POAG cross-ancestry association in locus rs74315329 (chr1_171636338_G_A_b38). Variants are color-coded by LD (r^2) relative to the sVariant with the highest colocalization posterior probability (chr1_172398537_A_G_b38; CLPP=0.12). **c-d,** LocusCompare plots of -$\log_{10}$(P-value) of the *PIGC* Spleen sQTL compared to POAG cross-ancestry GWAS meta-analysis before (**c**) and after (**d**) conditional analysis on the lead GWAS variants 74315329 (see Methods). Points are color-coded based on their LD (r^2) relative to the variant with the highest CLPP (chr1_172398537_A_G_b38). **e**, Violin plot of normalized intron-excision ratio for chr1:172370311-172376554 computed with Leafcutter for *PIGC* in Spleen as a function of the genotype of the sVariant rs10911684 (chr1_172398537_A_G_b38) with the highest CLPP for this sQTL and POAG locus. The effect size of the sQTL relative to the alternative allele (*beta* = 0.76) is in opposite direction relative to the POAG cross-ancestry association (*beta* = -0.085), suggesting that increased exon 2 skipping is protective of POAG. **f**, Gene model for *PIGC* in GTEx Spleen on positive strand, showing all splicing events in the gene (open circles), including the splice event chr1:172370311-172376554 (from exon 1 to exon 3) with a significant sQTL (chr1_172398537_A_G_b38) that colocalized with POAG locus rs74315329 (Red line and closed circle).

Supplementary Figure 14. Top gene sets enriched for e/sGenes that colocalized with POAG and IOP GWAS loci.

a

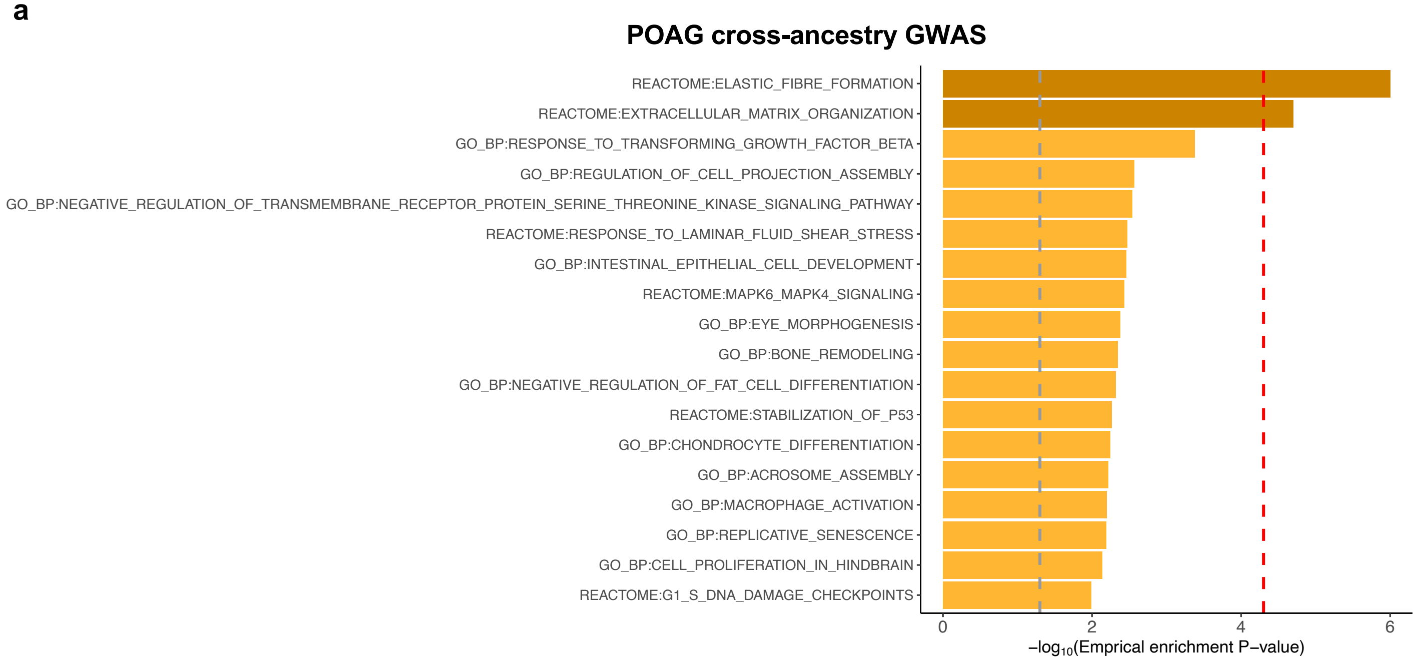

b

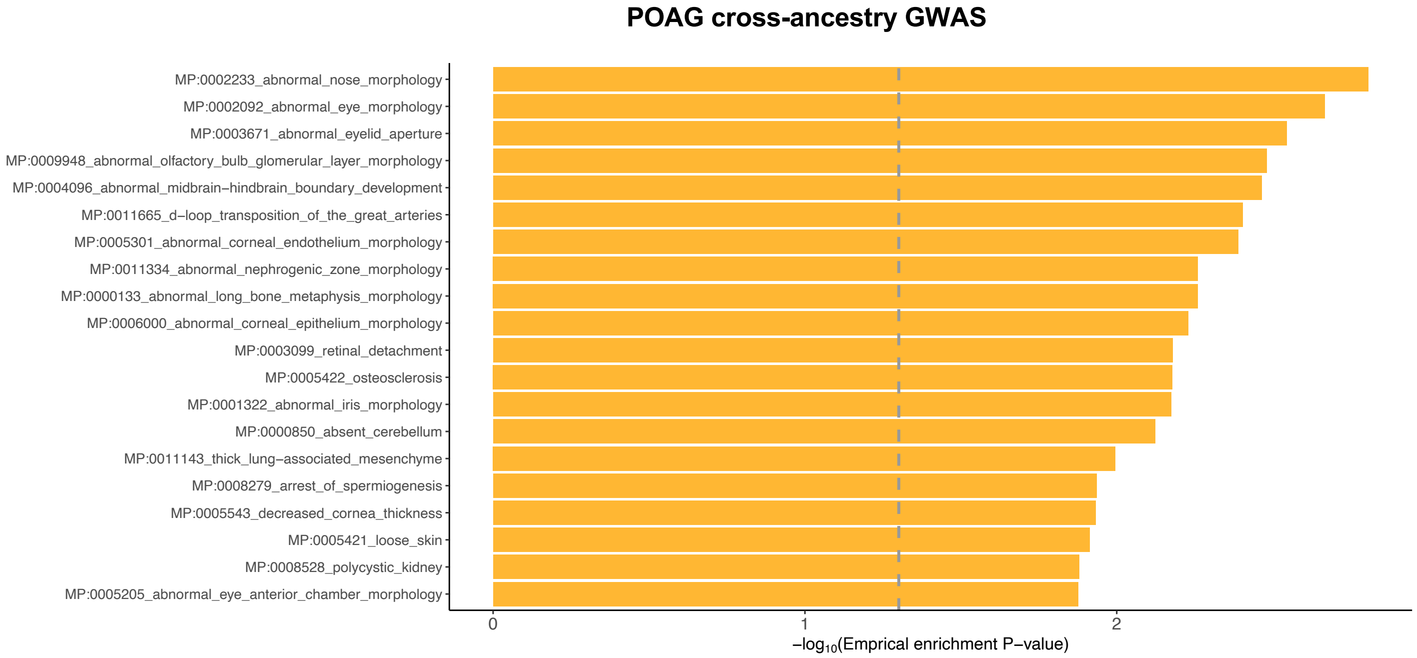

Supplementary Figure 14. Top gene sets enriched for e/sGenes that colocalized with POAG and IOP GWAS loci.

c

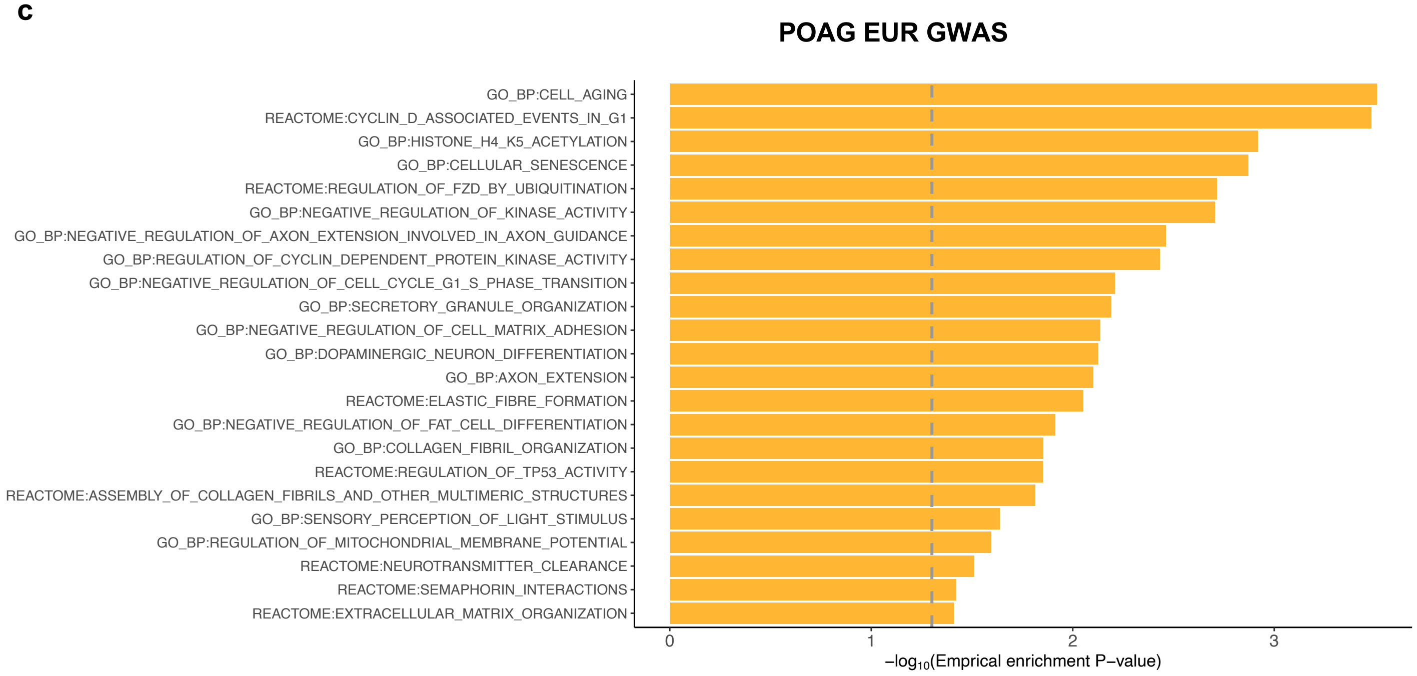

d

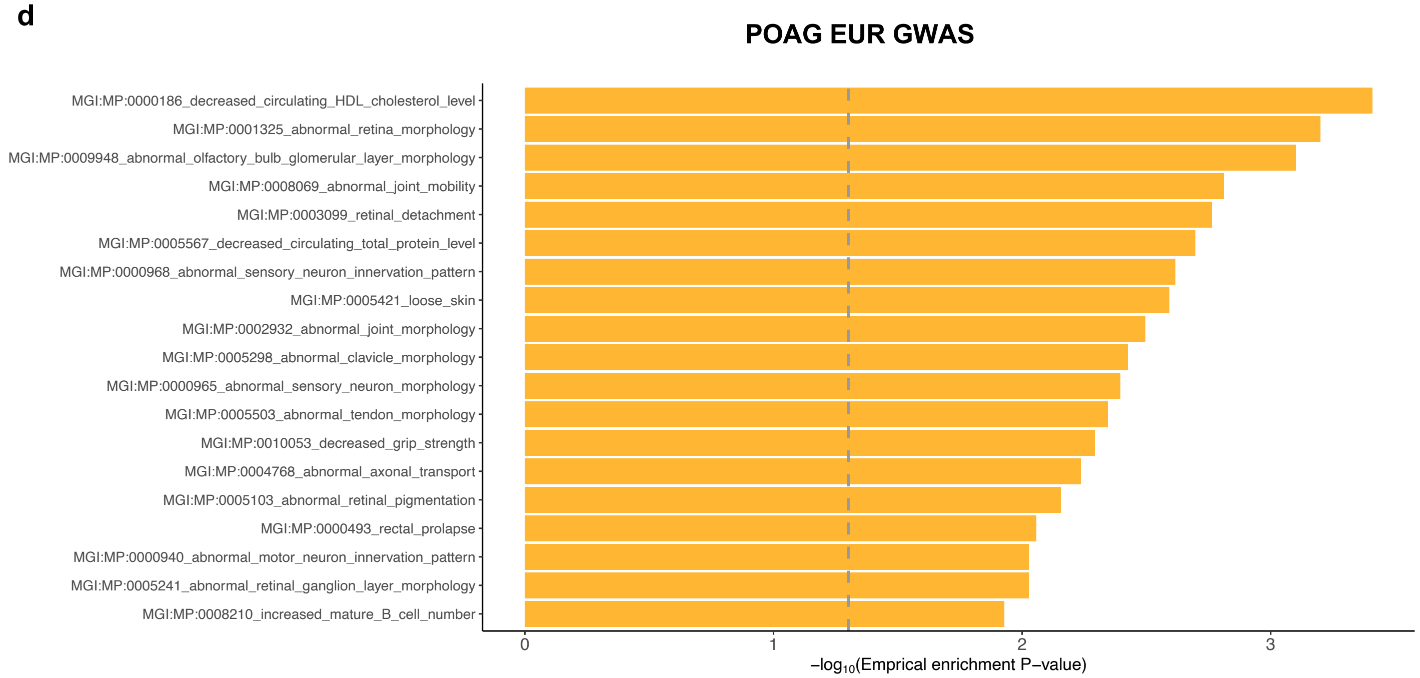

Supplementary Figure 14. Top gene sets enriched for e/sGenes that colocalized with POAG and IOP GWAS loci.

e

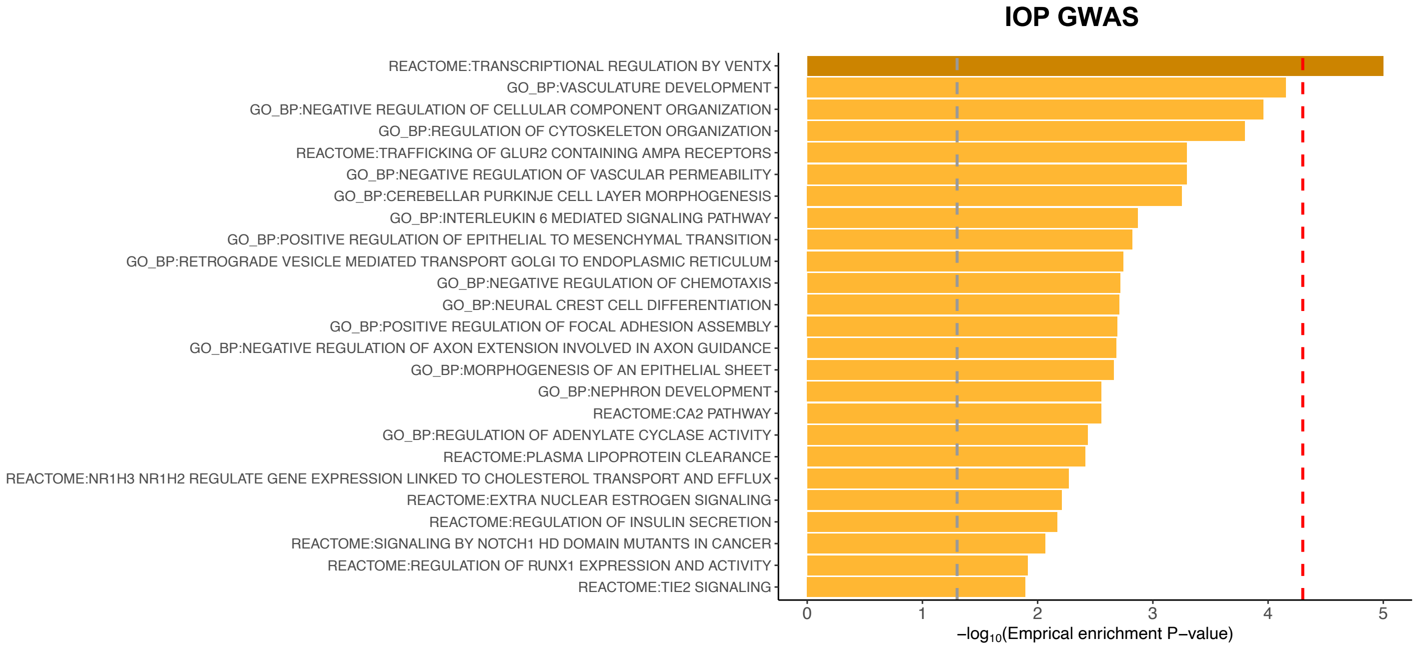

f

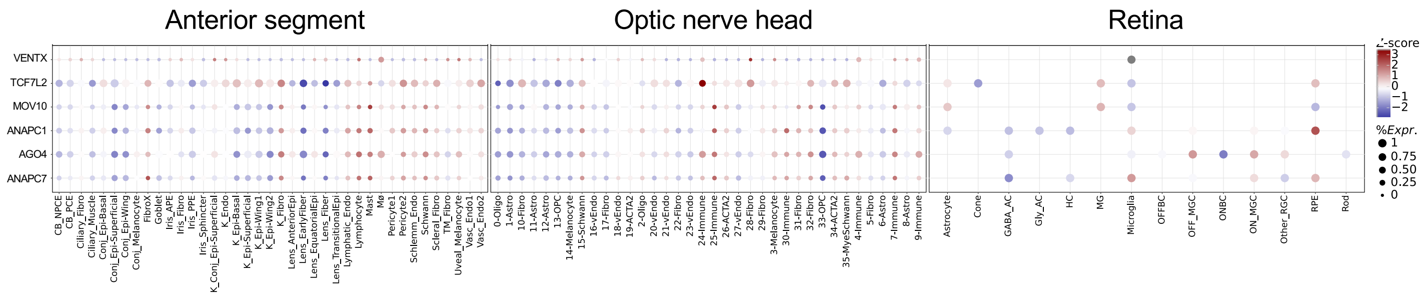

g

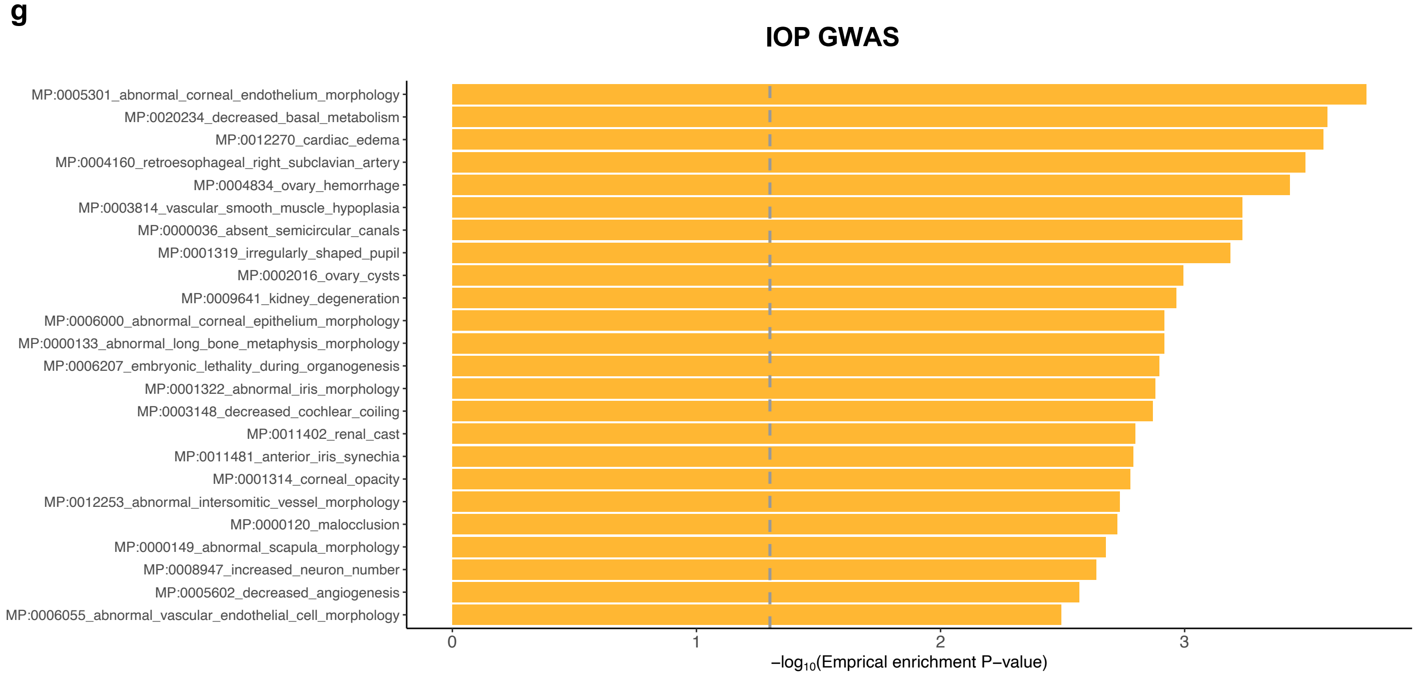

Supplementary Figure 14. Top gene sets enriched for e/sGenes that colocalized with POAG and IOP GWAS loci. Barplots of top ranked gene sets enriched for target genes of colocalizing e/sQTLs with POAG Cross-ancestry (**a**,**b**), POAG European ancestry subset (**c**,**d**), and IOP (**e**,**g**) GWAS loci are shown for biological processes (Reactome and gene ontology) (**a**,**c**,**e**) and mouse phenotype ontology (**b**,**d**,**g**) gene sets separately. An empirical gene set enrichment p-value computed with *GeneEnrich* is shown on $-\log_{10}$ scale. Dark orange bars indicate gene sets that pass Bonferroni correction (Emp. P < $2x10^{-5}$; red dashed line), and yellow bars indicate gene sets with a nominal Emp. P < 0.05 (dashed grey line). **f,** Bubble map displaying the expression of VENTX and five VENTX target genes, whose e/sQTLs colocalized with IOP and were enriched in the regulation by VENTX gene set (shown in panel **e**), across all cell types in the anterior segment, optic nerve head, and retina. The colorbar represents z-scores computed by comparing each gene's average expression in a given cell type to its per cell type average expression across all types divided by the standard deviation of all cell type expression averages. Bubble size is proportional to the percentage of cells expressing the given gene (log(TPK+1)>1). Cell type abbreviations are described in Supplementary Data 35.

Supplementary Figure 15. Cell type enrichment of e/sQTL-mapped genes in POAG and IOP GWAS loci in the anterior segment.

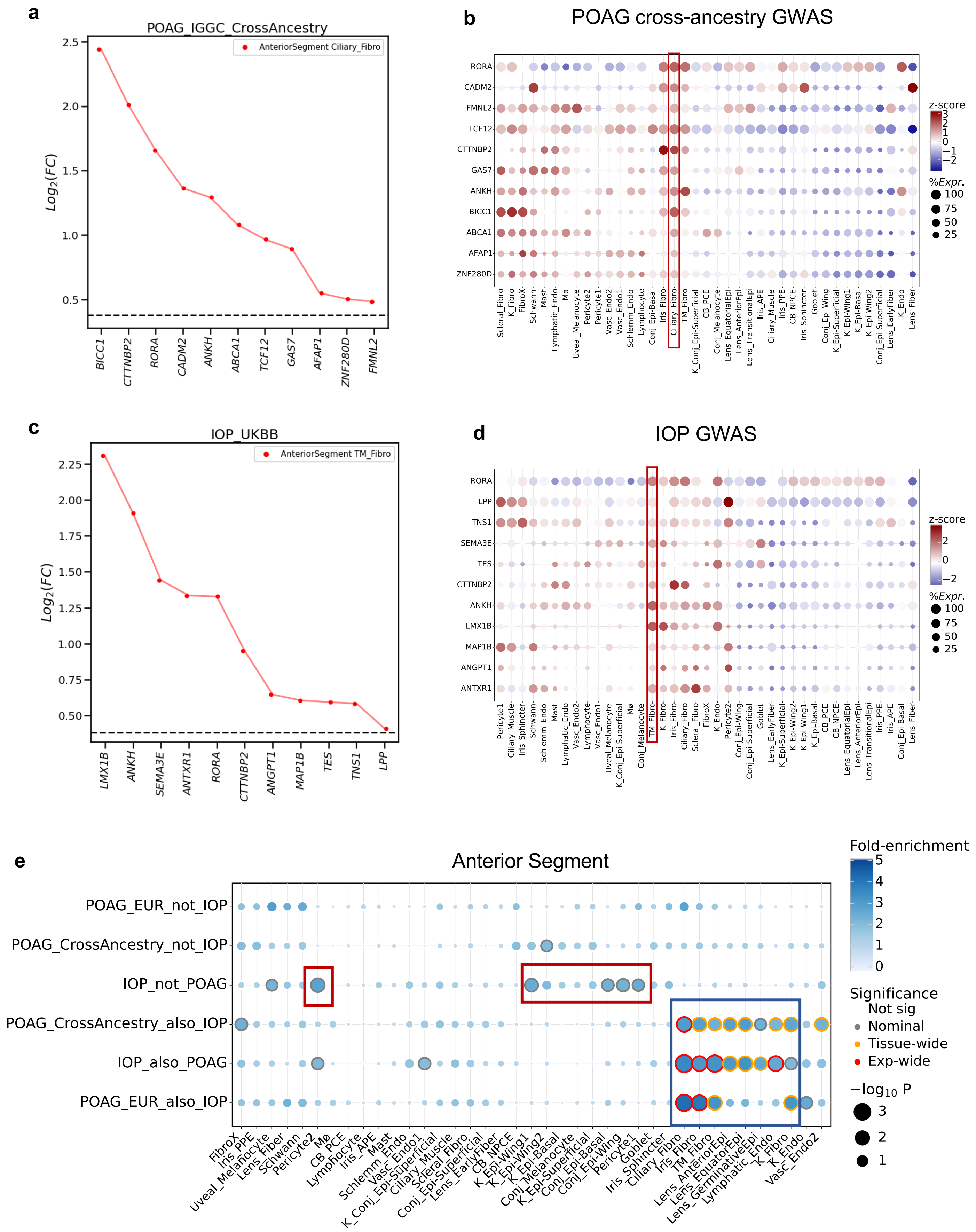

Supplementary Figure 15. Cell type enrichment of e/sQTL-mapped genes in POAG and IOP GWAS loci in the anterior segment. a, Differential expression (log_2(Fold-change), y axis) of the e/sGenes mapped to POAG cross-ancestry GWAS loci based on colocalization analysis, which are driving the enrichment signal in ciliary fibroblasts (P=0.01) in the anterior segment compared to all other cell types in the anterior segment. Horizontal dashed line represents log_2(Fold-change) of 0.375 (FC=1.3) and FDR<0.1 that was used as the cell type-specificity enrichment cutoff. **b**, Bubble map displaying the expression of the e/sGenes driving the POAG enrichment in ciliary fibroblasts across all cell types in the anterior segment. Colorbar represents gene expression z-scores computed by subtracting each gene's average across all cell types from its average expression in a given cell type, divided by the standard deviation of the gene's average expression across all cell types. Bubble size is proportional to the percentage of cells expressing the given gene (log(TPK+1)>1). Cell type abbreviations are described in Supplementary Data 35. Ciliary fibroblasts displayed the most significant enrichment of all cell types in the anterior segment for POAG cross-ancestry GWAS. **c,** Differential expression (log_2(Fold-change)) of the e/sGenes mapped to IOP GWAS loci in Trabecular meshwork (TM) fibroblasts compared to all other cell types in the anterior segment. Specifications similar to panel **a**. **d**, Bubble map displaying the expression of the e/sGenes driving the IOP enrichment in TM fibroblasts across all cell types in the anterior segment. Specifications similar to panel **b**. **e,** Significance (circle size, -log10(P-value)) and fold-enrichment (circle color) of the cell type specificity of GWAS locus sets for POAG and IOP independent and shared loci, based on ECLIPSER, for all cell types in the anterior segment. Traits (rows) and cell types (columns) were clustered based on hierarchical clustering of the Euclidean distance between GWAS locus set cell type-specificity enrichment scores. Red boxes point to cell type enrichment in IOP only loci. Blue boxes point to cell type enrichment in POAG and IOP shared loci. Cell type specific genes driving the cell type enrichment results can be found in Supplementary Data 38.

Supplementary Figure 16. Cell type enrichment of e/sQTL-colocalizing genes in POAG and IOP GWAS loci in two separate retina snRNA-seq studies.

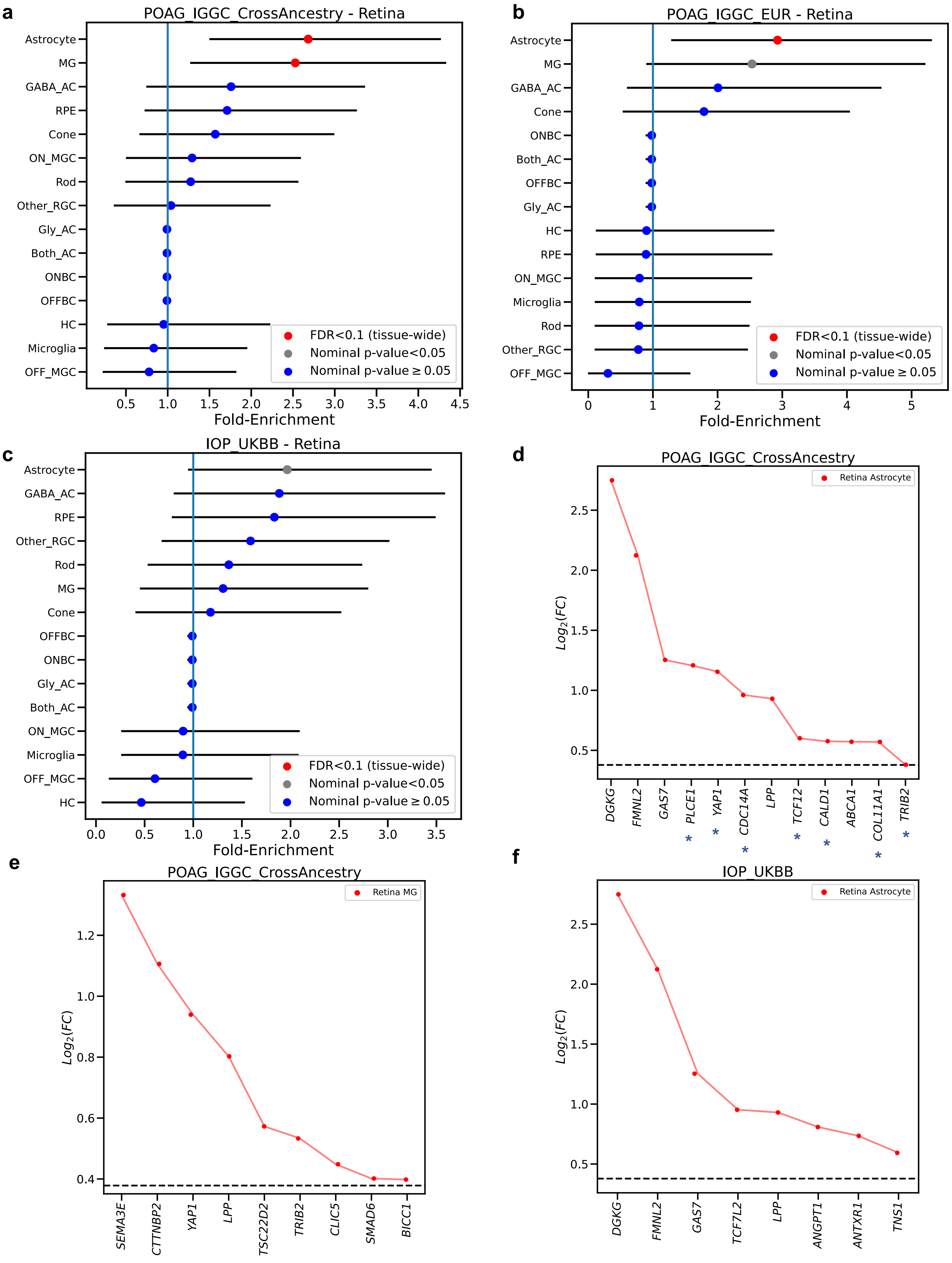

g

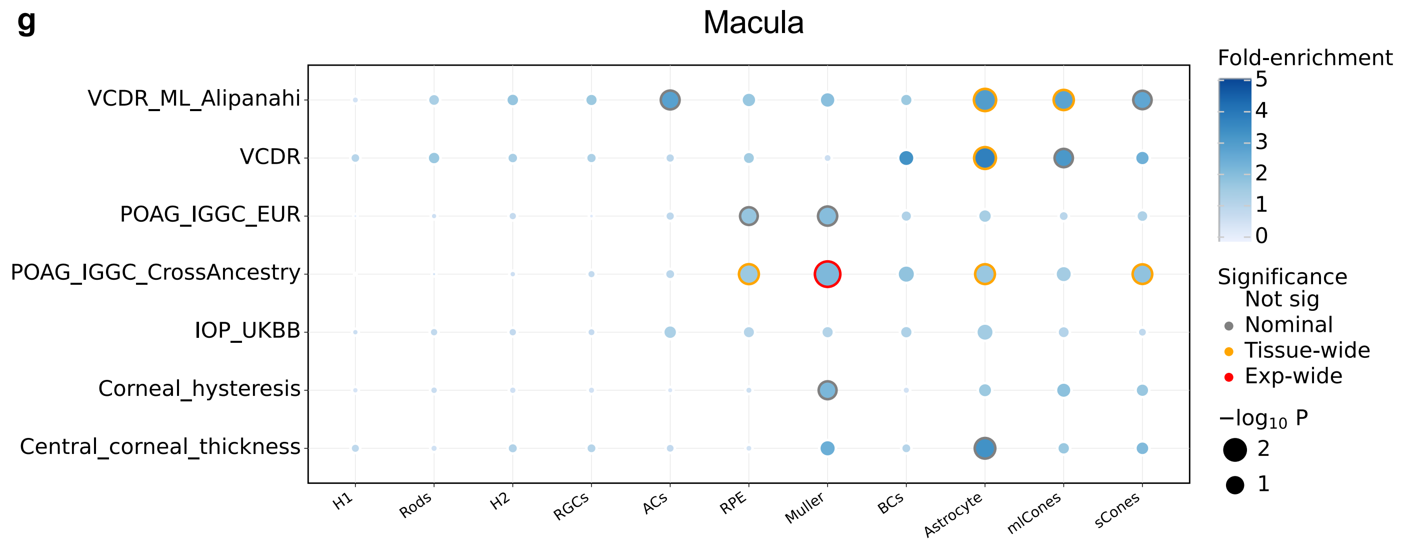

Supplementary Figure 16. Cell type enrichment of e/sQTL-colocalizing genes in POAG and IOP GWAS loci in two separate retina snRNA-seq studies. a-c, Cell type specificity fold-enrichment (x-axis) based on ECLIPSER in retina cell types ranked in descending order for the POAG cross-ancestry (**a**), POAG European subset (**b**), and IOP (**c**) GWAS locus sets. Red: tissue-wide significant (FDR<0.1); Grey: nominal significant (P<0.05); Blue: non-significant (P≥0.05). **d-f,** Differential expression (log_2(Fold-change), y axis) of the GWAS colocalizing e/sGenes driving the enrichment signal in astrocytes (**d**,**f**) and Müller Glia cells (**e**) for POAG cross-ancestry GWAS loci (**d-e**) and IOP (**f**) compared to all other cell types in the retina. Horizontal dashed line represents log_2(Fold-change) of 0.375 (FC=1.3) and FDR<0.1 that was used as the cell type-specificity enrichment cutoff. **g,** Significance (circle size, -log10(P-value)) and fold-enrichment (circle color) of the cell type specificity of GWAS locus sets for POAG cross-ancestry, POAG European subset, IOP, physician-defined Vertical-cup-to-disc ratio (VCDR), machine learning (ML)-defined ver (VCDR_ML_Alipanahi), central cornea thickness, and corneal hysteresis GWAS, based on ECLIPSER, are shown for each of the 11 cell types found in macula, a separate single nucleus RNA-seq dataset than the retina dataset in **a-f**. Traits (rows) and cell types (columns) were clustered based on hierarchical clustering of the Euclidean distance between GWAS locus set cell type-specificity enrichment scores. Red rings: experiment-wide significant (Benjamini Hochberg (BH) FDR<0.1); Yellow rings: tissue-wide significant (BH FDR<0.1); Grey rings: nominal significant (P<0.05). H1, horizontal cells cluster 1; H2, horizontal cells cluster 2; RGCs, retinal ganglion cells; ACs, amacrine cells; RPE, retinal pigment epithelium; MG, Müller Glia; BCs, bipolar cells; mlCones, M-cone and L-cone photoreceptors; sCones, S-cone photoreceptors.

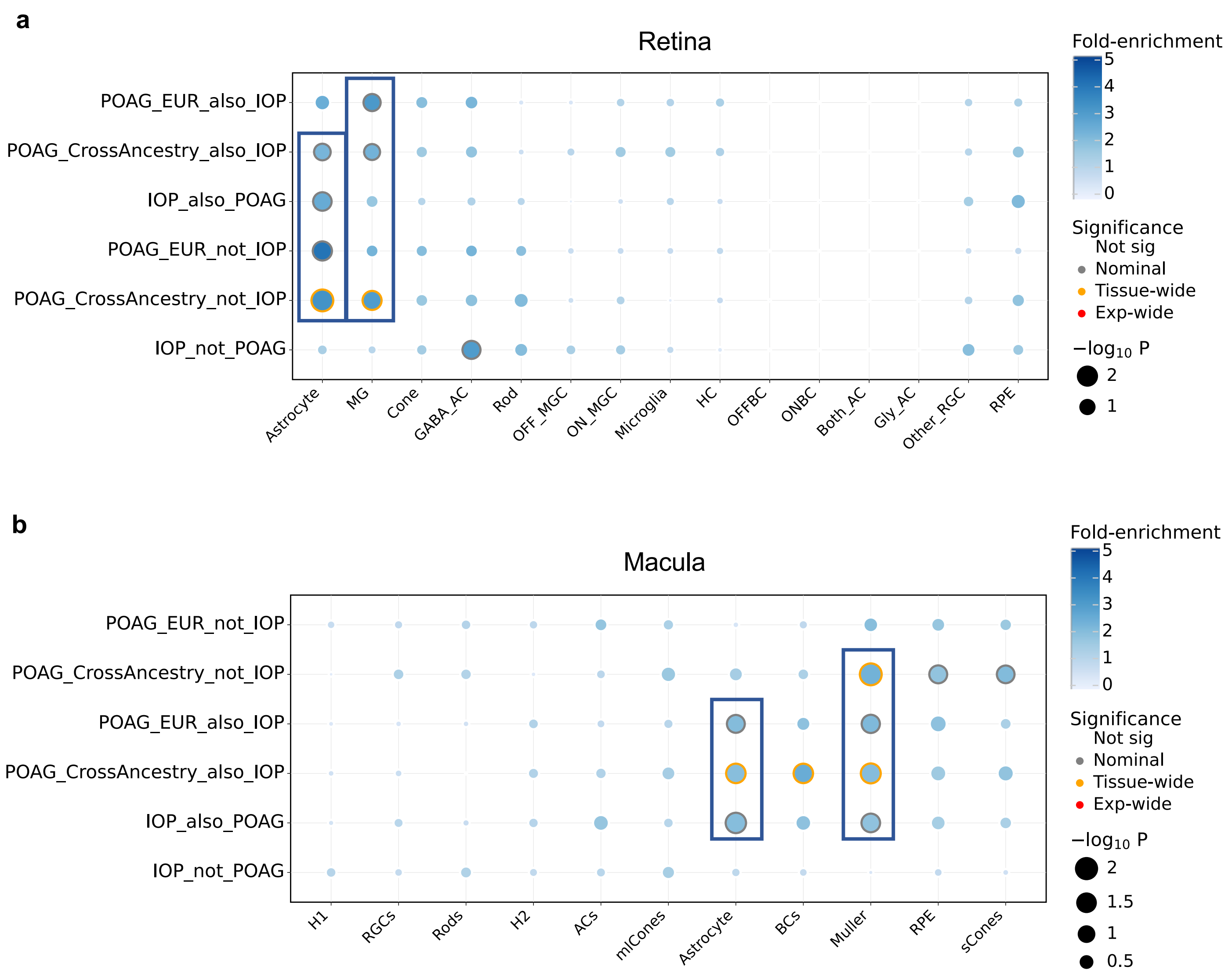

Supplementary Figure 17. Cell type enrichment of e/sQTL-colocalizing genes with POAG and IOP independent and shared GWAS loci in retina and macula. Significance (circle size, -log10(P-value)) and fold-enrichment (circle color) of the cell type specificity of GWAS locus sets for POAG and IOP independent and shared loci, based on ECLIPSER, for all cell types in peripheral and macular retina (**a**) and in separate macula (**b**) snRNA-seq studies. Traits (rows) and cell types (columns) were clustered based on hierarchical clustering of the euclidean distance between GWAS locus set cell type-specificity enrichment scores. Blue boxes point to cell type enrichment in POAG only or POAG and IOP shared loci. GABA_AC, GABAergic amacrine cells; Gly_AC, Glycinergic amacrine cells; Both_AC, amacrine cells with both glycinergic and GABAergic markers; ONBC, ON-bipolar cells; OFFBC, ON-bipolar cells; RPE, retinal pigment epithelium; ON_MGC, ON-midget ganglion cells; OFF_MGC, OFF-midget ganglion cells; Other_RGC, All the retinal ganglion cells (RGCs) other than the ON- and OFF-MGC (over 90% belong to these two groups); HC, horizontal cells; MG, Müller Glia. H1, horizontal cells cluster 1; H2, horizontal cells cluster 2; RGCs, retinal ganglion cells; ACs, amacrine cells; Muller, Müller Glia; BCs, bipolar cells; mlCones, M-cone and L-cone photoreceptors; sCones, S-cone photoreceptors.

Supplementary Figure 18. Cell type enrichment of e/sQTL-colocalizing genes with POAG and IOP GWAS loci in optic nerve head and surrounding posterior tissues.

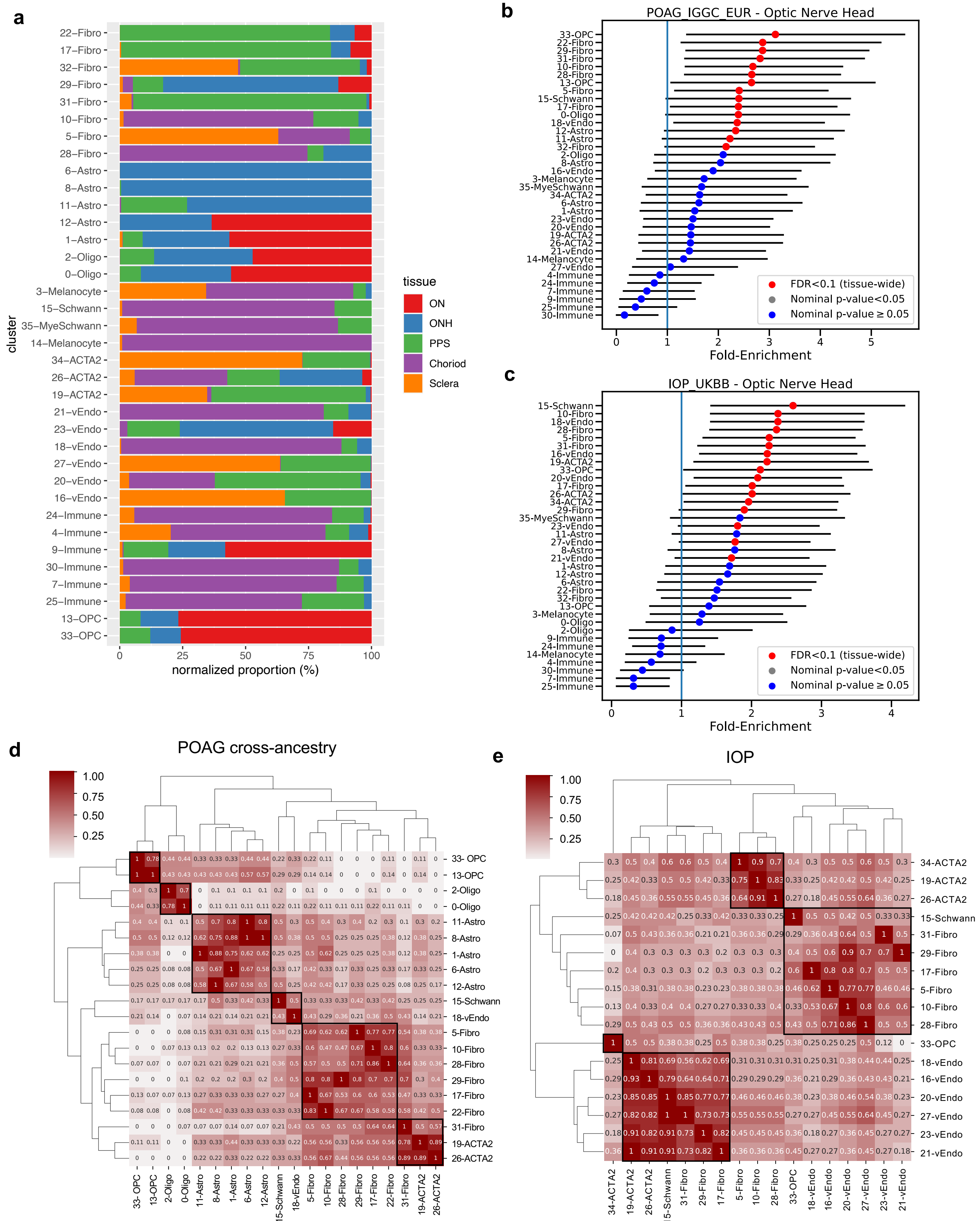

Supplementary Figure 18. Cell type enrichment of e/sQTL-colocalizing genes with POAG and IOP GWAS loci in optic nerve head and surrounding posterior tissues.

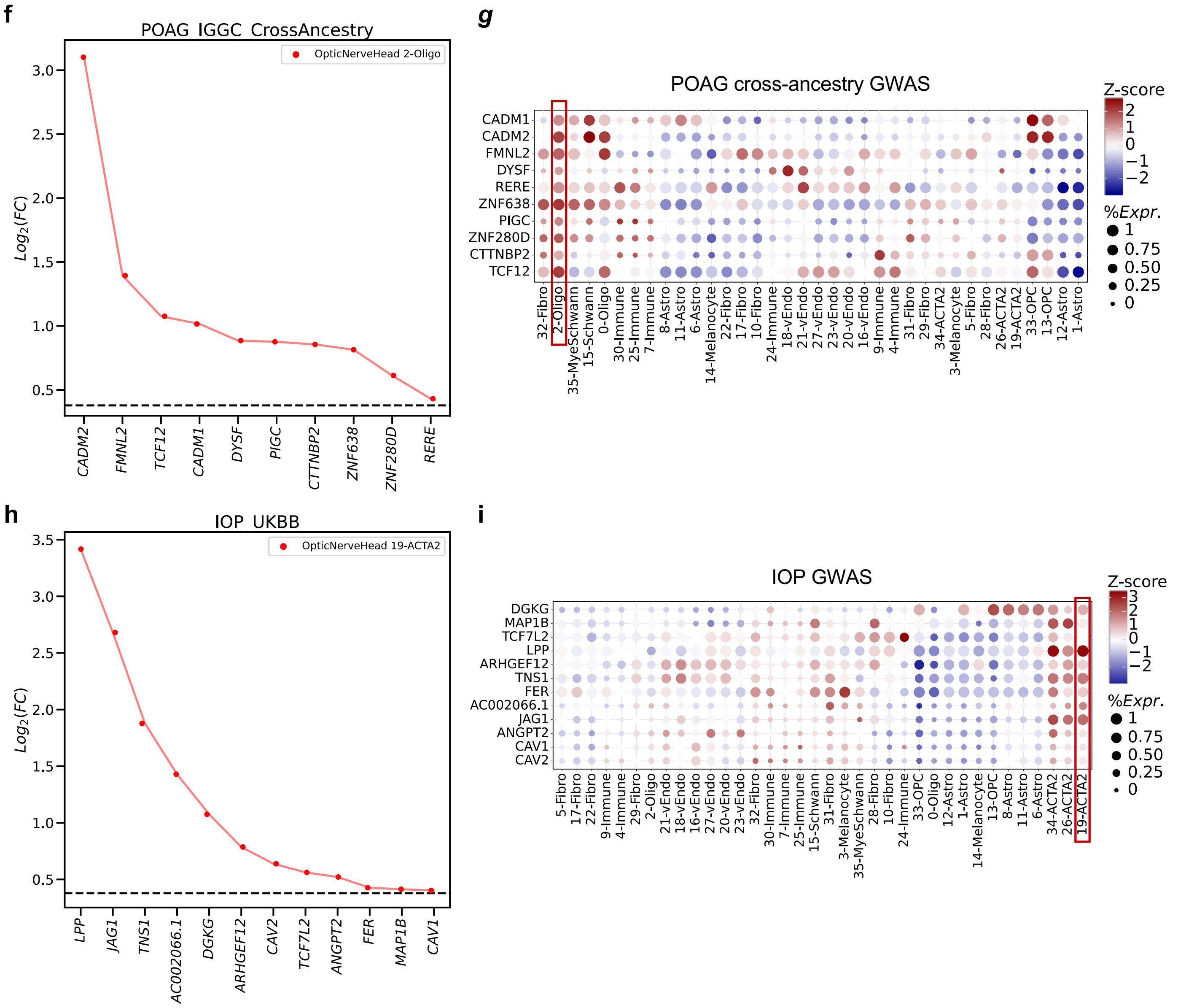

Supplementary Figure 18. Cell type enrichment of e/sQTL-colocalizing genes with POAG and IOP GWAS loci in optic nerve head and surrounding posterior tissues. a, Barplot displaying the proportion distribution of each cell type in the optic nerve head (ONH), optic nerve (ON), peripapillary sclera (PPS), peripheral sclera, and choroid adapted from Monavarfeshani*, Yan* *et al.,* bioRxiv 2023[37]. **b,c,** Cell type specificity fold-enrichment (x-axis) based on ECLIPSER in the ONH and surrounding posterior tissue cell types ranked in descending order for the POAG European (**b**) and IOP (**c**) GWAS locus sets. Red: tissue-wide significant (FDR<0.1); Grey: nominal significant (P<0.05); Blue: non-significant (P≥0.05). **d,e,** Heatmap of fraction of genes that overlap between the e/sGenes driving the enrichment signal for top ranked cell types (ECLIPSER P<0.05) in the posterior tissues for POAG cross-ancestry (**d**) and IOP (**e**) GWAS loci. Numbers refer to the fraction of e/sGenes driving the cell type enrichment on each row that overlaps with the genes driving the cell type enrichment on the corresponding column. Hierarchical clustering was performed on both rows and columns using the euclidean distance between fractions. Black boxes show the main clades. **f,h,** Differential gene expression (log_2(Fold-change), y axis) in an enriched cell type compared to all other cell types for the set of genes (x axis) driving the enrichment signal of POAG cross-ancestry GWAS loci in oligodendrocytes in ONH and ON **(f)** and IOP GWAS loci in vascular smooth muscle cells (19-ACTA2) **(h).** Horizontal dashed line represents log_2(Fold-change) of 0.375 (FC=1.3) and FDR<0.1 that was used as the cell type-specificity enrichment cutoff. **g,i,** Bubble maps displaying the expression of the e/sGenes driving the POAG gene enrichment in oligodendrocytes **(g)** or IOP gene enrichment in in vascular smooth muscle cells **(i)** across all cell types in the ONH and adjacent posterior tissues. The colorbar represents gene expression z-scores computed by comparing each gene's average expression in a given cell type to its per cell type average expression across all types divided by the standard deviation of all cell type expression averages. Bubble size is proportional to the percentage of cells expressing the given gene (log(TPK+1)>1). OPC, oligodendrocyte precursor cells; 19-ACTA2 and 34-ACTA2, vascular smooth muscle cell types in the PPS and sclera. Cell type abbreviations are described in Supplementary Data 35.

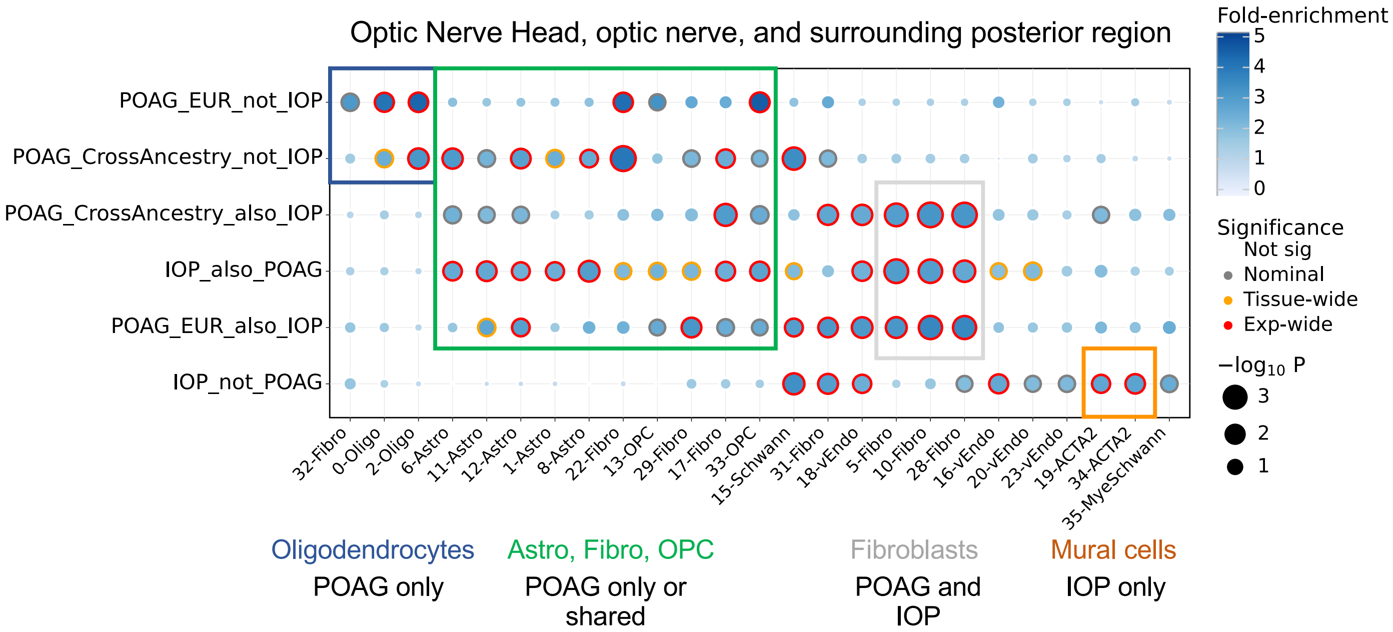

Supplementary Figure 19. Cell type enrichment of e/sQTL-colocalizing genes with POAG and IOP independent and shared GWAS loci in optic nerve head and surrounding posterior tissues. Significance (circle size, -log10(P-value)) and fold-enrichment (circle color) of the cell type specificity of GWAS locus sets for POAG and IOP independent and shared loci, based on ECLIPSER, for all cell types with at least one significant result in the optic nerve head, optic nerve, peripapillary sclera (PPS), peripheral sclera, and choroid. Traits (rows) and cell types (columns) were clustered based on hierarchical clustering of the Euclidean distance between GWAS locus set cell type-specificity enrichment scores. Astro, astrocytes; Fibro, fibroblasts; OPC, oligodendrocyte precursor cells; 19-ACTA2 and 34-ACTA2, vascular smooth muscle cell types (mural cells) in the peripapillary sclera (PPS) and sclera. Cell type-specific genes driving the cell type enrichment results and the cell type abbreviations can be found in Supplementary Data 38.

Supplementary Figure 20. Cell type specific expression of e/sQTL-colocalizing genes with POAG and IOP GWAS loci in glaucoma-relevant eye tissues.

a

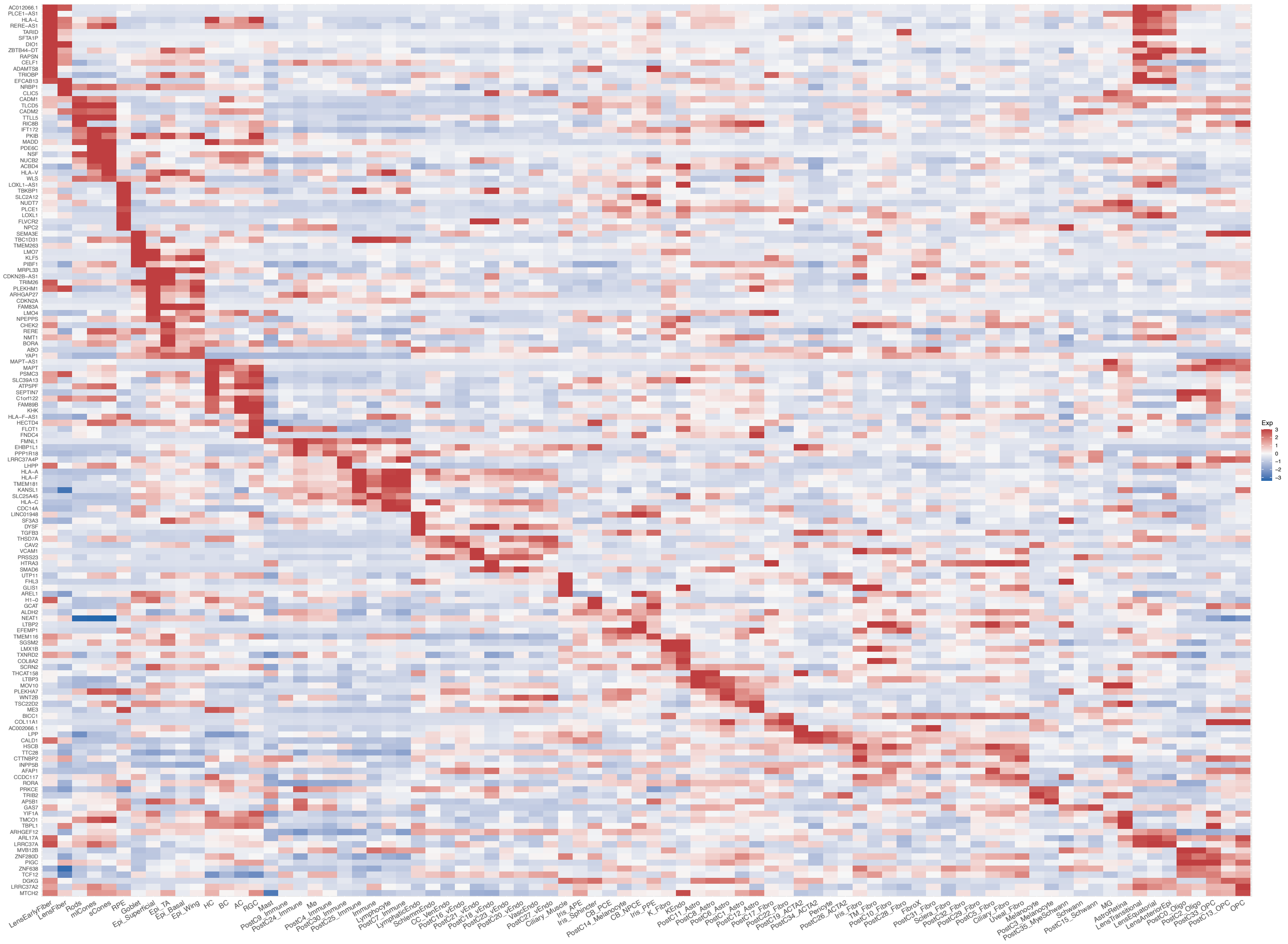

Supplementary Figure 20. Cell type specific expression of e/sQTL-colocalizing genes with POAG and IOP GWAS loci in glaucoma-relevant eye tissues.

b

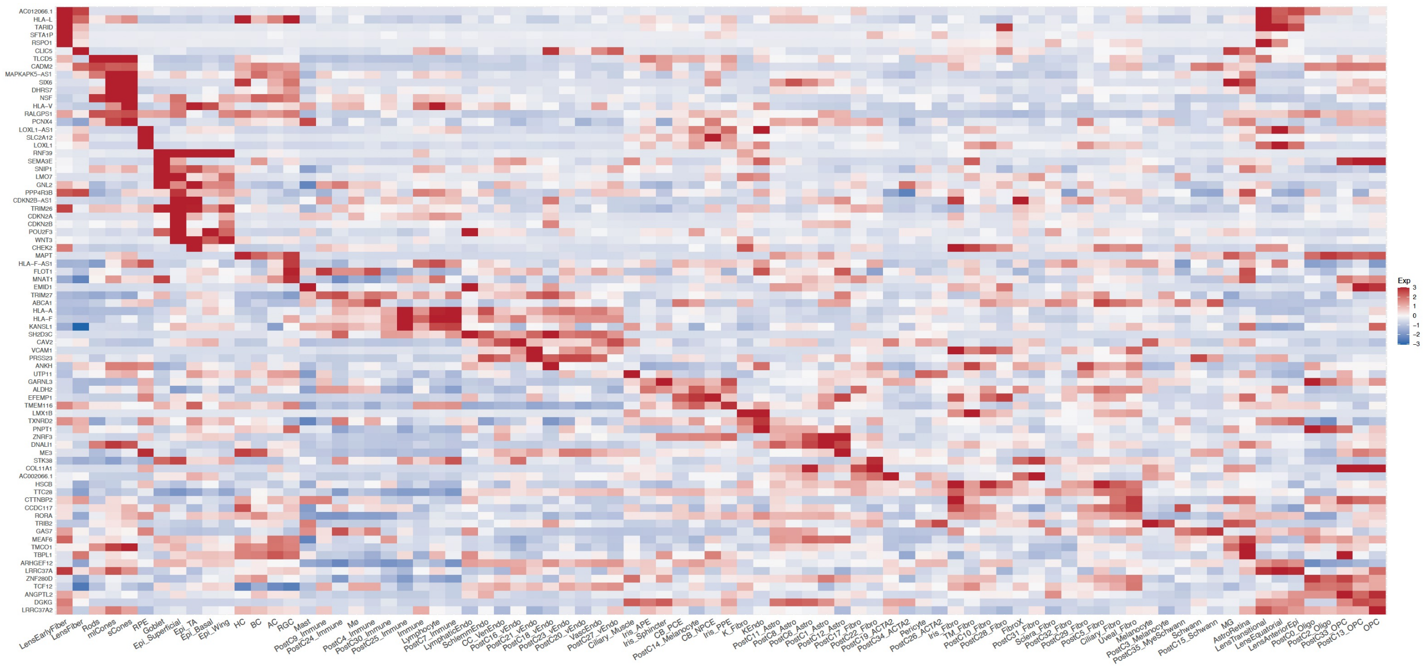

c

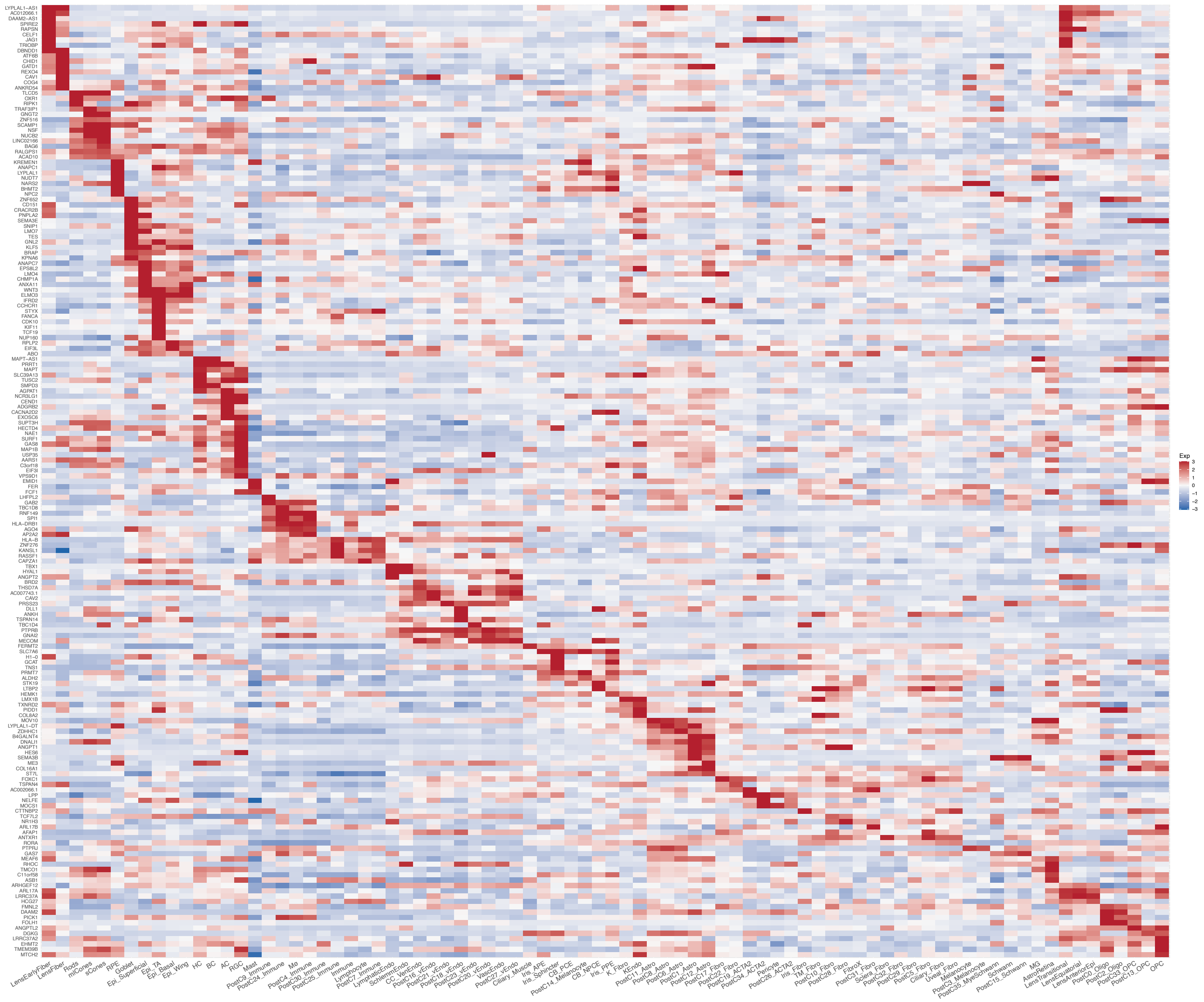

Supplementary Figure 20. Cell type specific expression of e/sQTL-colocalizing genes with POAG and IOP GWAS loci in glaucoma-relevant eye tissues. a-c, Heatmap displaying scaled mean expression (z-scores) of the genes whose e/sQTLs significantly colocalized with POAG cross-ancestry (**a**), POAG European subset (**b**) and IOP (**c**) GWAS loci across anterior segment, retina, and optic nerve head/optic nerve/peripapillary sclera/sclera/choroid (Post) cell types. Only genes expressed in more than 10% of cells in any cell type are shown (146, 77 and 179 genes for POAG cross-ancestry, POAG EUR and IOP loci, respectively). Colorbar represents gene expression z-scores computed by subtracting each gene's average across all cell types from its average expression in a given cell type, divided by the standard deviation of the gene's average expression across all cell types. Hierarchical clustering was performed on both rows (genes) and columns using the Euclidean distance between z-score vectors. Some of these data are replotted from Figure S6B in Monavarfeshani *, Yan* *et al., P*NAS 2023.

Supplementary Figure 21. Cell type specific enrichment of e/sQTL-mapped genes to negative control trait loci in anterior and posterior eye tissues.

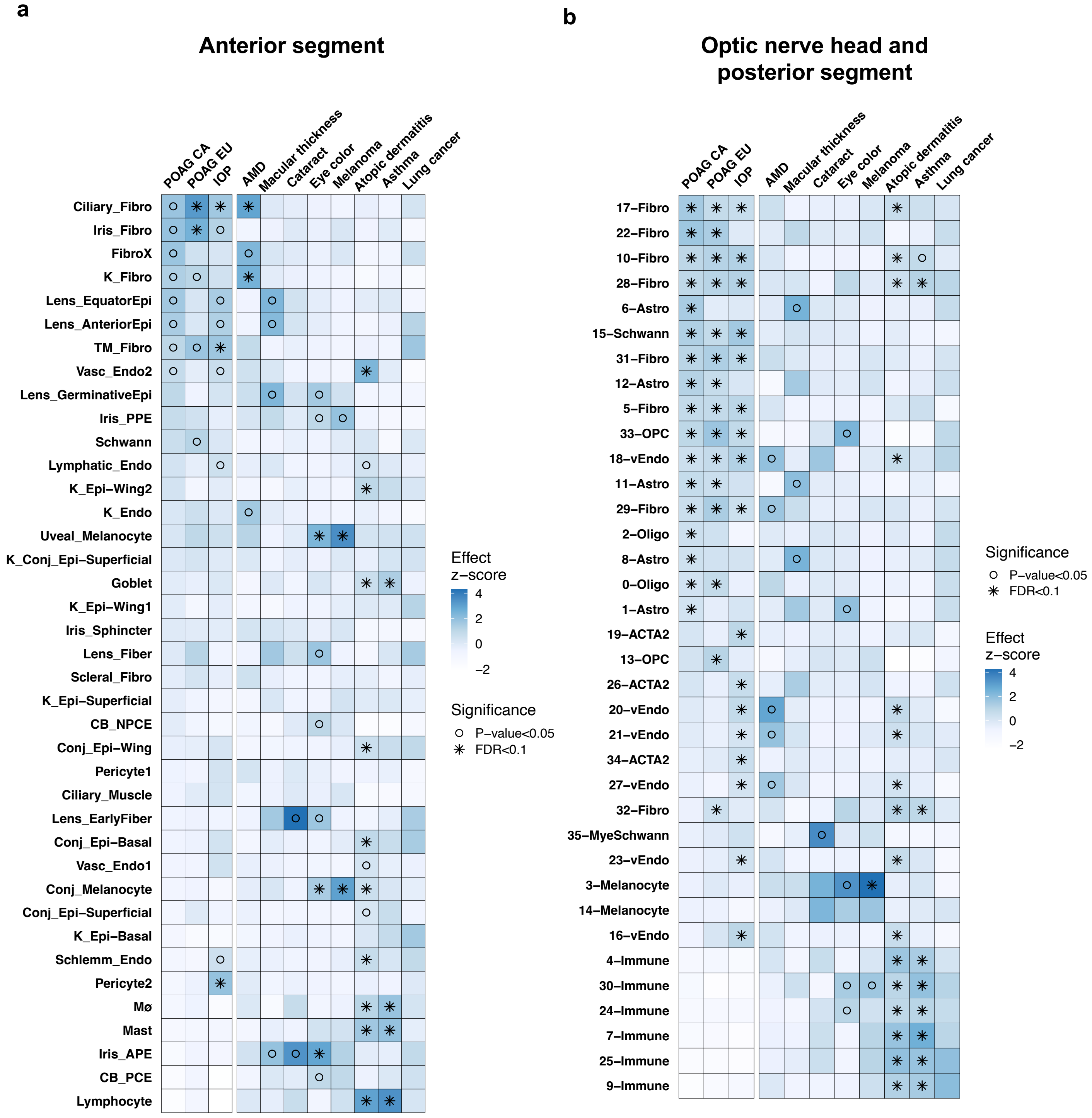

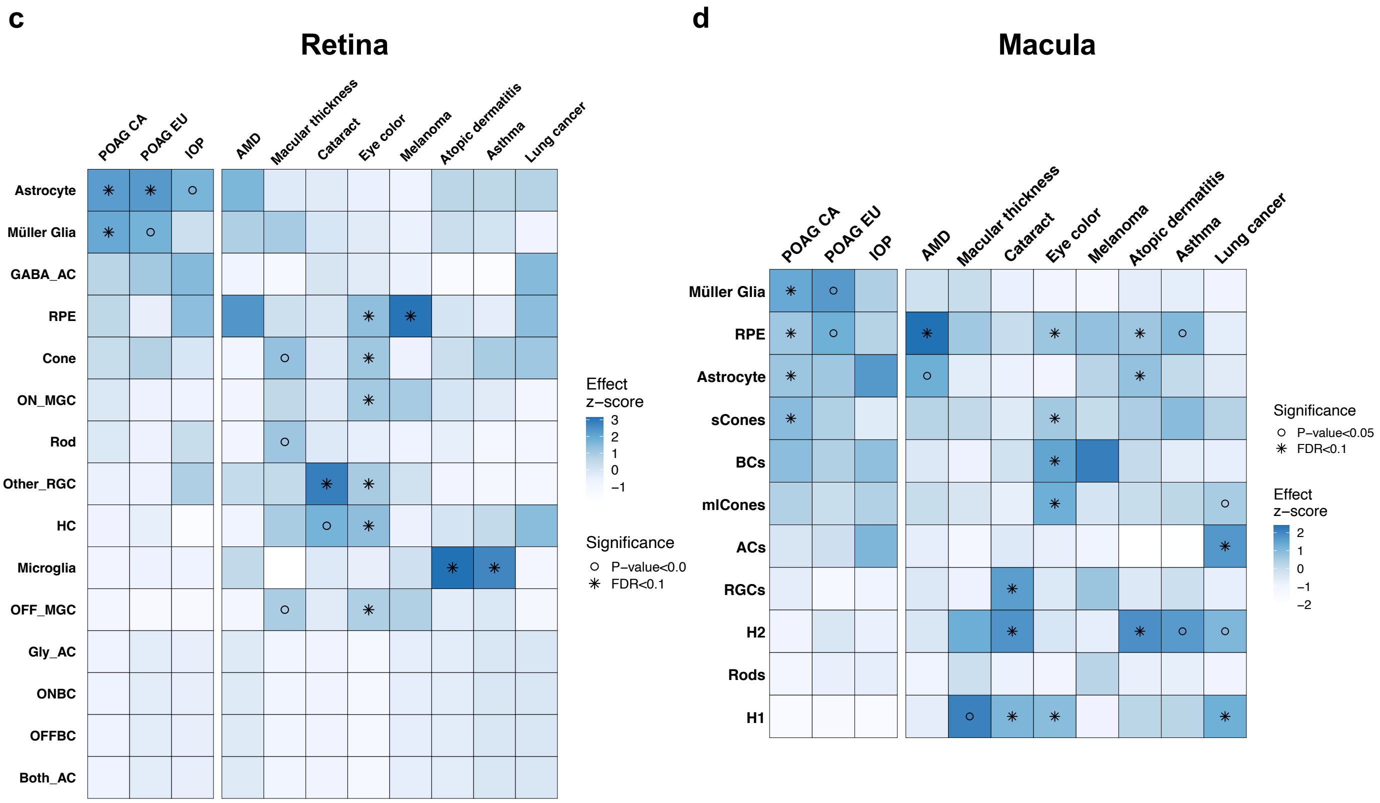

Supplementary Figure 21. Cell type specific enrichment of e/sQTL-mapped genes to negative control trait loci in eye tissues. a-d, Heatmaps displaying scaled ECLIPSER effect size (z-scores) of cell type fold-enrichment for POAG cross-ancestry (CA), POAG European subset (EU), and IOP GWAS loci, followed by eight negative control traits using four single nucleus RNA-sequencing datasets: the anterior segment **(a),** optic nerve head, optic nerve and surrounding posterior tissues **(b),** peripheral and macular retina **(c),** and macula **(d).** The blue colormap is proportional to the effect z-score. Rows are ordered by the POAG CA effect sizes per cell, sorted in descending order. Asterisks represent significant cell types at Benjamini-Hochberg FDR below 0.1, and circles represent nominal significance (P<0.05). Genes were mapped to POAG and IOP GWAS loci based on colocalization analysis, and to the negative control traits based on the target genes of GTEx and retina e/sQTLs that are in linkage disequilibrium (LD; r^2>0.8) to the lead GWAS variants. Cell type abbreviations are described in Supplementary Data 35. AMD, age-related macular degeneration.

Supplementary Figure 22. Cell type specific enrichment of POAG and IOP associations in anterior and posterior eye tissues based on three different methods.

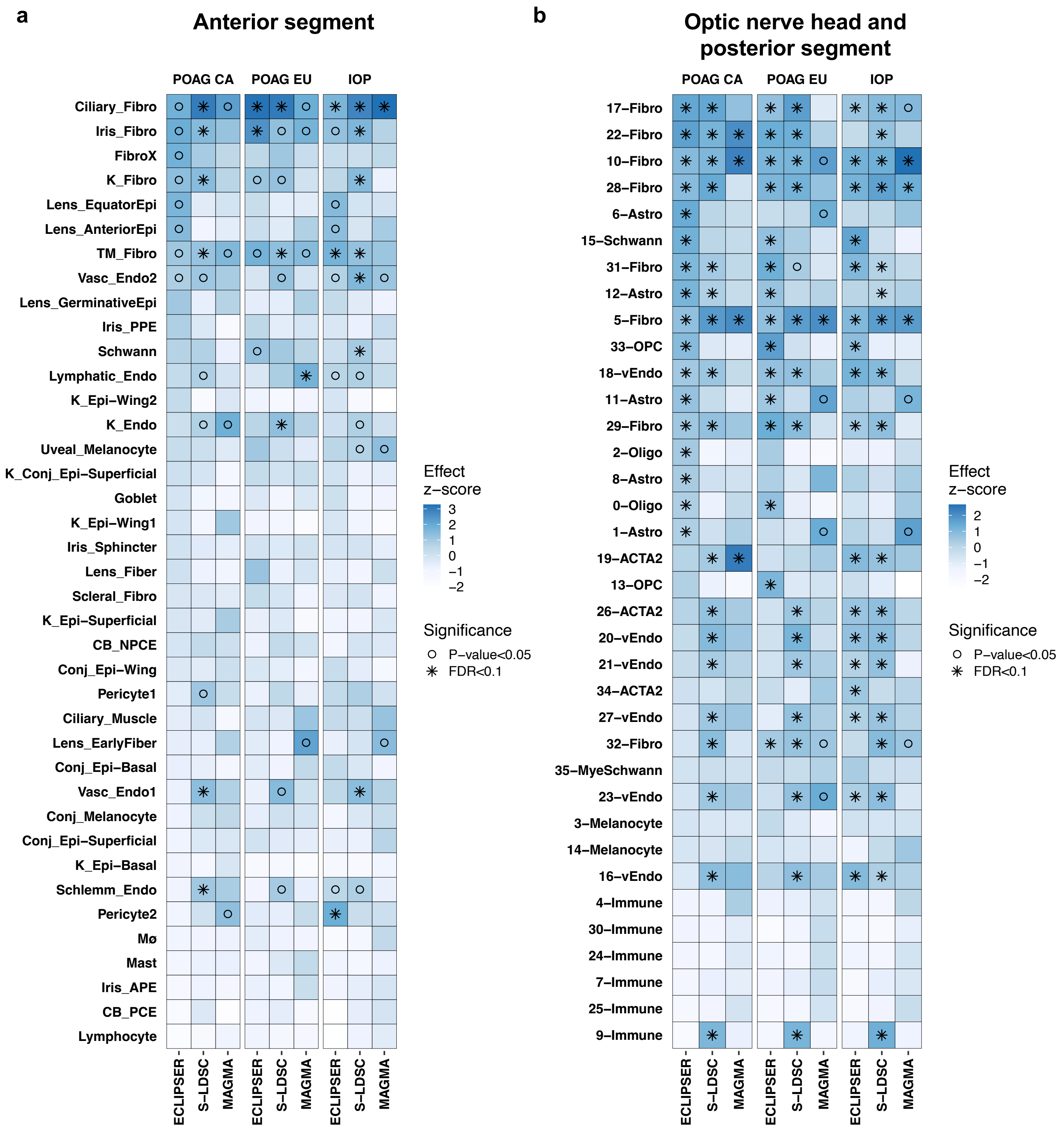

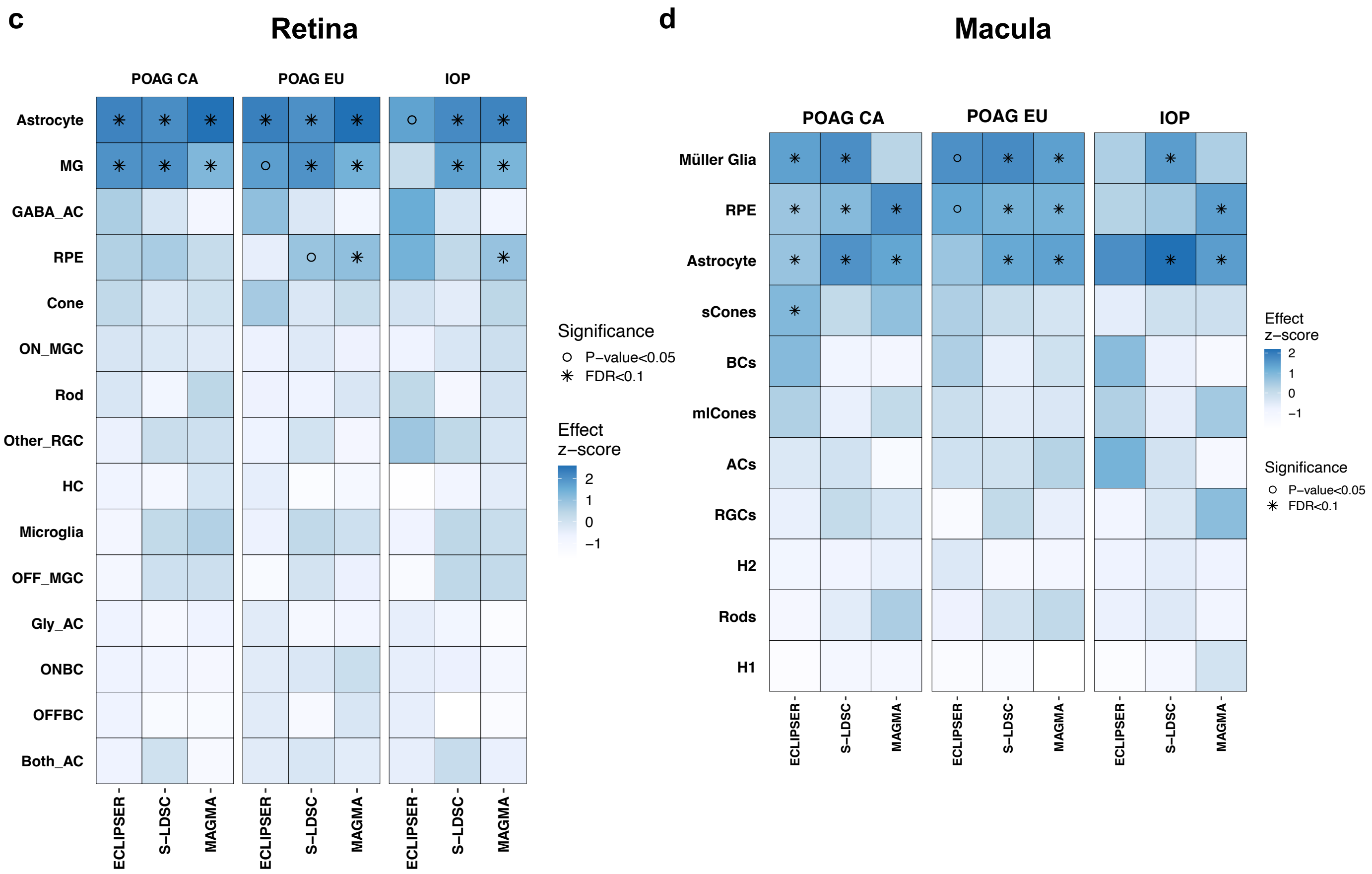

Supplementary Figure 22. Cell type specific enrichment of POAG and IOP associations in anterior and posterior eye tissues based on three different methods. Heatmaps displaying scaled cell type enrichment effect sizes (z-scores) of POAG cross-ancestry (CA), POAG European subset (EU), and IOP GWAS associations, based on ECLIPSER, stratified LD score regression (S-LDSC) and MAGMA methods for four single nucleus RNA-sequencing datasets: the anterior segment **(a),** optic nerve head, optic nerve and surrounding posterior tissues **(b),** peripheral and macular retina **(c),** and macula **(d).** The blue colormap is proportional to the effect z-score. Rows are ordered by the POAG CA effect sizes per cell with ECLIPSER, sorted in descending order. Asterisks represent significant cell types at Benjamini-Hochberg FDR below 0.1, and circles represent nominal significance (P<0.05). Genes were mapped to POAG and IOP GWAS loci based on colocalization analysis for the ECLIPSER analysis, and genome-wide GWAS associations were used in S-LDSC and MAGMA cell type enrichment analysis. Cell type abbreviations are described in Supplementary Data 35. LD, linkage disequilibrium.

Supplementary Figure 23. Cell type enrichment of genes mapped to VCDR and cornea-related trait GWAS loci in glaucoma-relevant ocular tissues.

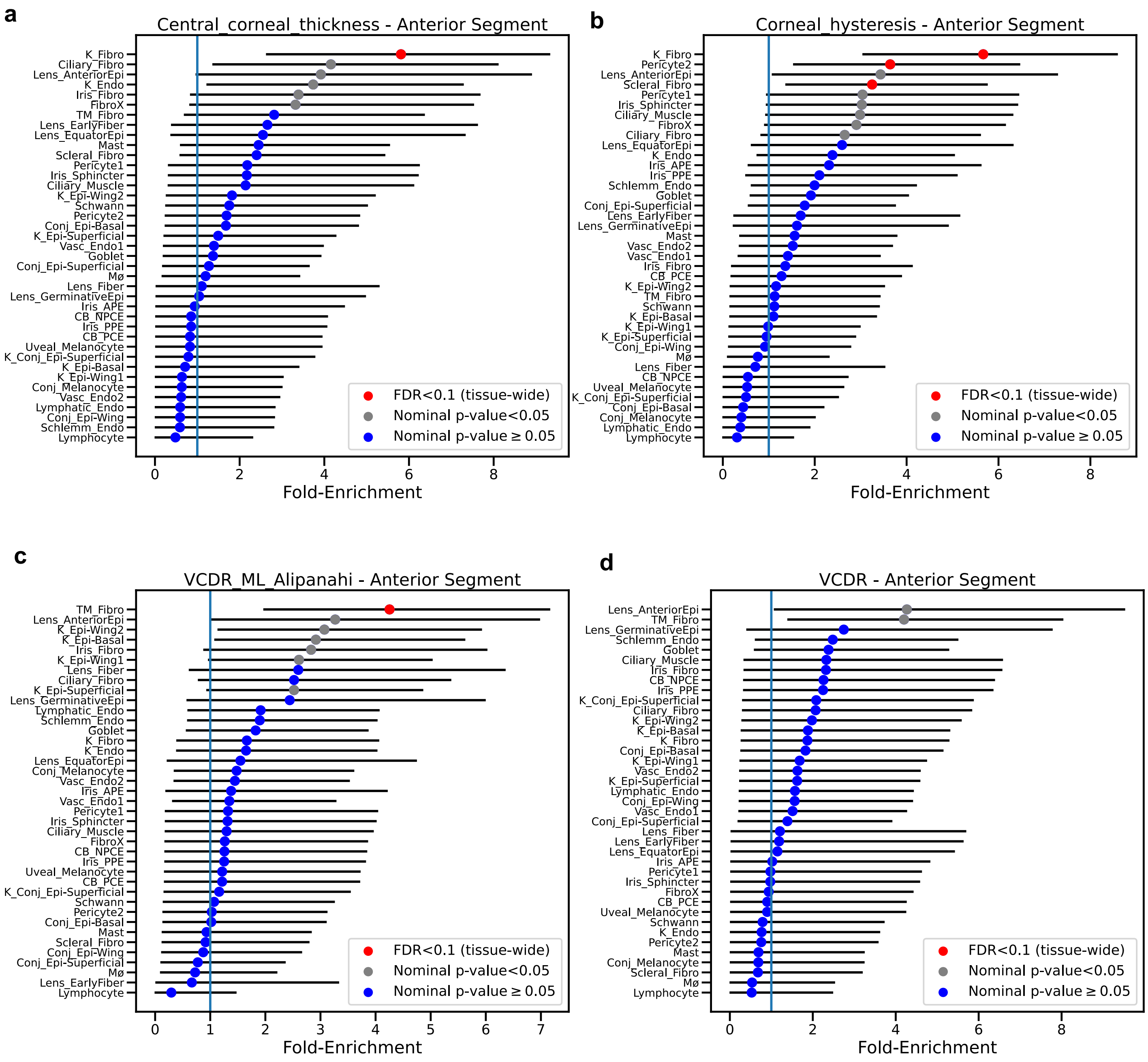

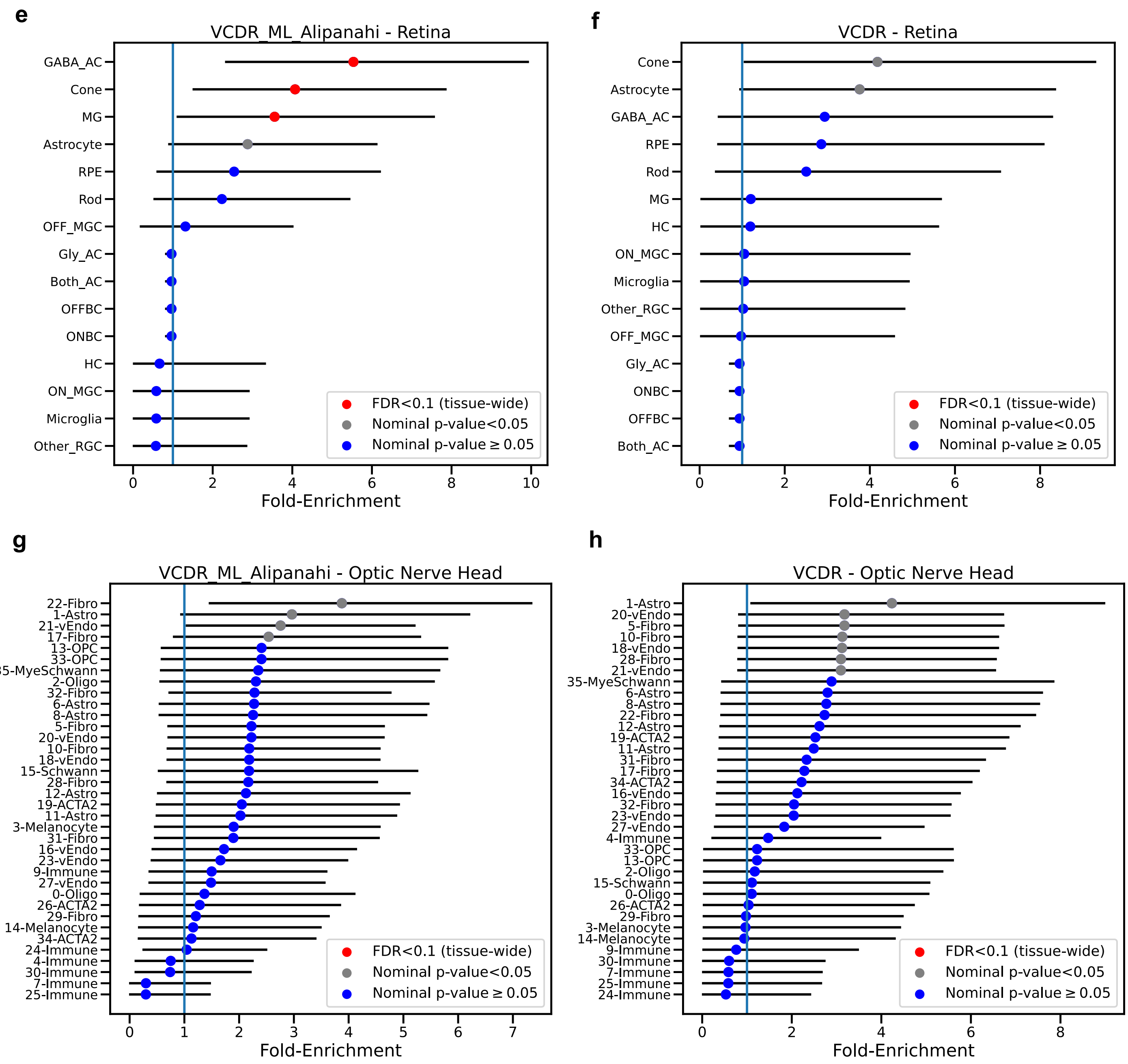

Supplementary Figure 23. Cell type enrichment of genes mapped to VCDR and cornea-related trait GWAS loci in glaucoma-relevant ocular tissues. a-i, Cell type specificity fold-enrichment (x-axis) based on ECLIPSER in the anterior segment (**a-d),** retina (**e-f**), and optic nerve head and surrounding posterior tissues (**g-h**) cell types ranked in descending order for the central corneal thickness (**a**), corneal hysteresis (**b**), machine learning (ML)-defined vertical-cup-to-disc ratio (VCDR) (**c,e,g**), and physician-defined VCDR (VCDR) (**d,f,h**) GWAS locus sets. Red: tissue-wide significant (FDR<0.1); Grey: nominal significant (P<0.05); Blue: non-significant (P≥0.05). Cell type abbreviations are described in Supplementary Data 35.

Supplementary Methods

Enrichment of POAG and IOP associations among e/sQTLs using *QTLEnrich.* To test whether genome-wide significant and nominal POAG and IOP trait associations are enriched among eQTLs and sQTLs, and to assess the contribution of e/sQTLs to these traits, we applied *QTLEnrich*[1] (https://github.com/segrelabgenomics/QTLEnrich) to the POAG and IOP GWAS meta-analyses summary statistics[2,3], and eQTLs and sQTLs from the 49 GTEx tissues[1,4] and eQTLs from peripheral retina (EyeGEx[4]) (https://gtexportal.org/home/datasets). *QTLEnrich*[5] is a rank and permutation-based method that evaluates the fold-enrichment significance of trait associations among a set of e/sQTLs in a given tissue, compared to a tissue-specific null distribution of variants matched on three confounding factors: minor allele frequency (MAF), distance to the target gene's transcription start site, and local LD. Briefly, fold-enrichment was computed for each trait by e/sQTL-tissue combination as the fraction of e/sVariants (FDR<0.05) with a GWAS P-value<0.05 compared to expectation, assuming a uniform null distribution. Fold-enrichment was also computed for $<10^5$ randomly sampled sets of *k* null variants (non-significant e/sVariants; *k* = number of significant e/sVariants in given tissue) matched on the three confounding factors relative to each significant e/sVariant. Random sampling was applied to decile bins of the confounding factors with replacement. The null background of variants was defined as all common variants (MAF>1%) tested for e/sQTL in GTEx or retina excluding the significant e/sVariants (FDR<0.05). The enrichment p-value (one-sided) was computed as the fraction of up to 10^5 null variant sets whose fold-enrichment was equal to or higher than that of the significant set of e/sVariants for a given tissue. To adjust for the potential enrichment of trait associations amongst null variants, an adjusted fold-enrichment was computed per trait-e/sQTL-tissue combination as the observed fold-enrichment divided by the median fold-enrichment of 1000 confounder-matched null variant sets. Local LD was estimated based on the number of LD proxy variants ($R^2 > 0.5$) per variant, using GTEx WGS v8 VCF as the reference panel. For the enrichment analysis only the most significant e/sVariant per e/sGene (FDR<0.05) in the given tissue was used to prevent inflation of trait association enrichment due to LD. In cases where the e/sQTL was the most significant e/sVariant for more than one e/sGene, the smallest distance to TSS was used. Only protein coding and lincRNA genes were considered in this analysis. Significant tissues were determined based on an Enrichment P-value that passed Bonferroni correction, correcting for 50 tissues and two QTL types tested ($P<5\times10^{-4}$). The adjusted fold-enrichment was used to rank the significantly enriched tissues, as this statistic is not correlated with tissue sample size or number of significant e/sQTLs per tissue[5], in contrary to that observed

with the colocalization analysis (Supplementary Fig. 4). For the significant trait-tissue pairs, the fraction and number of e/sVariants proposed to be associated with POAG or IOP were estimated using an empirically derived, true positive rate (Adj. π_1) approach implemented in the latest version of *QTLEnrich*, based on Storey's analytical π_1[6] and an empirical FDR method[7]. The adj. π_1 adjusts for enrichment of trait associations amongst non-significant e/sQTL variants matched on confounding factors. A lambda of 0.8 was used to estimate the adjusted true negative rate. The estimated number of trait associations amongst a set of e/sVariants in a given tissue was computed as the adj. π_1 times the number of e/sQTLs analyzed for that tissue. In few cases, the adj. π_1 can be zero for e/sQTLs that display significant enrichment of trait associations. This may be caused by deflation of the GWAS p-values of the e/sQTL set at the higher end of p-values that are used to estimate the true negative rate. *QTLEnrich* thus also estimates a lower bound number of trait associations among e/sQTLs with GWAS P<0.05 that is not affected by this issue (Supplementary Data 2-3, column 'Estimated_num_trait_associations_with_GWAS_p<0.05').

Colocalization analysis:

eCAVIAR analysis. eCAVIAR[8] (https://github.com/fhormoz/caviar) is a probabilistic model that estimates the posterior probability that the same variant is causal in overlapping GWAS and e/sQTL signals, accounting for the uncertainty of LD and allelic heterogeneity. It simultaneously performs statistical fine-mapping on the GWAS and e/sQTL signals to optimize integration. For the eCAVIAR analysis, we assumed at most two independent causal variants per locus[1]. Only variants present in both the GWAS and QTL studies were analyzed. LD matrices with R^2 values between all pairwise variant comparisons per locus were computed for each GWAS locus LD interval (defined above), using the GTEx whole genome sequencing (WGS) data from release v8 as the reference panel, and were used for fine-mapping of all variants in the GWAS and e/sQTL loci. The genotypes from all 838 GTEx donors in v8[1], consisting of European (84%), African (12.3%), and East Asian (1.4%) ancestries, were used for the LD calculations for the cross-ancestry POAG loci, and the European subset (715 donors) was used for the European POAG and IOP loci. Due to computational limitations, for GWAS LD intervals with more than 1000 variants, variants with e/sQTL p-values below 0.05 were filtered out. GWAS-e/sQTL-tissue combinations with a colocalization posterior probability (CLPP) above 0.01 were considered significant in this study (listed in Supplementary Data 7-9), based on previous simulations that suggested a high true positive rate and low false positive rate at CLPP > 0.01[8]. Colocalization analysis of the retina eQTLs was only performed using eCAVIAR.

***enloc* analysis.** *enloc*[9] (Enrichment estimation aided colocalization analysis) is a Bayesian hierarchical model used to determine the probability of colocalization of molecular QTLs with GWAS signals, that consists of three steps: estimation of enrichment levels of QTLs among the GWAS hits, fine-mapping using an empirical Bayes method (DAP-G)[9], and colocalization analysis that estimates a regional colocalization probability (RCP) that is the sum of SNP-level colocalization probabilities (SCPs) of all variants per GWAS locus. *enloc* does not limit the potential number of independent causal variants. To estimate the posterior probability of causality (posterior inclusion probabilities (PIPs)) of variants in the GWAS loci we applied DAP-G (https://github.com/xqwen/dap/tree/master/dap_src) to all variants in the LD intervals defined above for the POAG cross-ancestry, POAG European, and IOP loci. For several loci, we restricted the maximum model size parameter (-msize) to 5, which represents the number of independent association clusters, due to memory limitations. DAP-G outputs were post-processed, selecting all fine-mapped variants and their associated cluster number and PIP for each GWAS locus which were inputted into *enloc*. For the GTEx e/sQTLs, we used similar DAP-G fine-mapping results published for all e/sQTLs in 49 GTEx tissues that were computed using the European subset of GTEx samples[10]. LD matrices were computed per GWAS locus using all GTEx WGS samples for the POAG cross-ancestry loci and the subset of European individuals in GTEx for the POAG EUR and IOP loci, as the reference panel. We used an LD-threshold of 0.75 (-ld_control) that determines the strength of genotype correlation between variants that belong to a given fine-mapped signal cluster, as used in the GTEx European subset e/sQTL analysis[10]. Finally, we applied *fastEnloc*[11] (https://github.com/xqwen/fastenloc), a faster implementation of *enloc*, to the fine-mapped results of the GWAS loci and overlapping GTEx e/sQTLs. Only e/sVariants included in a 25% or greater credible set of causal variants and that overlapped with the GWAS variants were analyzed in the colocalization analysis. The colocalization analysis outputted RCP values above $1x10^{-4}$ for each tested GWAS locus, trait, tissue, and gene or gene-intron excision cluster combination. An RCP above 0.1 was considered significant in this study, based on the method's recommendation[9,12]. In Supplementary Data 10-12, we listed the set of variants included in the 95% credible set for each independent GWAS locus, e/sQTL, gene and tissue colocalization signal (cluster). In cases where a significantly colocalizing sQTL that regulates an intron cluster mapped to more than one gene, all target genes were reported as significant.

Quality control and summary across the two colocalization methods. In inspecting the significant colocalization results and plotting the $-\log_{10}$(P-value) of the GWAS signal versus the $-\log_{10}$(P-value) of the e/sQTL signal, we noticed multiple cases where the GWAS P-values and/or the e/sQTL P-values of the variants with significant colocalization posterior probabilities

(CLPP>0.01 and/or RCP>0.1) were not significant (e.g., association P>0.05) or did not pass multiple hypothesis correction for the GWAS or e/sQTL studies (see examples in Supplementary Fig. 3). Thus, to remove potential false positives, we filtered out variant, gene, tissue, trait combinations where the e/sVariant with a significant colocalization result had a GWAS p-value above $1x10^{-5}$ or whose e/sQTL p-values was above $1x10^{-4}$ and/or did not pass FDR<0.05 (FALSE in column 'Pass_QC_QTL_FDR05_P1E04_GWAS_P1E05' in Supplementary Data 7-12). We chose a GWAS $P<10^{-5}$ cutoff, as it has been suggested that in many cases the lead GWAS variant is not the causal variant, but instead tagging the causal variant that may have not reached genome-wide significance[13]. We defined two sets of putative causal genes and regulatory mechanisms for POAG and IOP: a "comprehensive set" based on significant colocalization with at least one of the colocalization methods (CLPP > 0.01 and/or RCP > 0.1) (Supplementary Data 13), and a "high confidence set" based on significance with both methods (CLPP > 0.01 and RCP > 0.1; Table 1 and Supplementary Data 15). Since the number of significantly colocalizing e/sGenes correlated with the tissue sample size (coefficient of determination $R^2=0.72$, $P=1x10^{-14}$, Supplementary Fig. 4), we disregarded the tissue and only considered the QTL type and e/sGene when generating the list of prioritized causal genes per GWAS locus.

Mendelian randomization (MR) of significantly colocalizing genes. We used Mendelian randomization (MR)[14] to identify a high confidence set of e/sQTLs that may be causal to POAG and/or IOP of the significant e/sQTL-GWAS colocalization results. MR comprises the use of genetic variants within an instrumental variable (IV) framework to facilitate causal inference[15]. For the variants to be valid, three assumptions must be met: (1) Relevance: the variants associate robustly with the exposure, (2) Independence: the variants are independent of confounders of the exposure and outcome, and (3) Exclusion-restriction: the genetic variants influence the outcome only via the exposure, i.e., there is no horizontal pleiotropy. To instrument putative causal genes, for each e/sQTL, we selected a set of LD-independent variants (pairwise LD threshold of $r^2 < 0.1$) that associated with gene expression at $P < 5 \times 10^{-6}$. If there were no significant e/sVariants at $P < 5 \times 10^{-6}$ for a given e/sGene and tissue, FDR < 0.05 was used. LD pruning was performed using 1000 Genomes Project Phase 3 version 5[16] as the reference panel and PLINK v1.9 (https://www.cog-genomics.org/plink)[17]. Two-sample MR was applied to the summary statistics of the e/sQTLs (exposure) and POAG or IOP GWAS (outcome) for all colocalizing e/sQTL-GWAS locus pairs. This included 1,866 eQTLs targeting 408 genes and 1,068 sQTLs targeting 147 genes in a range of tissues that colocalized with POAG and/or IOP GWAS loci based on eCAVIAR and/or *enloc* (Supplementary Data 7-13). Effect alleles were aligned between the exposure and

outcome datasets to harmonize genetic associations between the two studies. The Wald ratio, i.e., the variant-outcome association beta divided by the variant-exposure association beta[18], was used for exposures where only one variant constituted the genetic instrument. Where multiple variants constituted the instrument for the targeted e/sGene, the inverse-variance weighted (IVW) method was used as the primary method for pooling variant-specific estimates[19]. We applied four additional methods to test the robustness of the results, given that the IVW approach assumes no horizontal pleiotropy: the simple-median[20], weighted-median[20], MR-Egger[21], and MR-PRESSO[22] methods. Horizontal pleiotropy was tested on cases with 3 or more instrumental variables, using the Egger-intercept test and the MR-PRESSO global heterogeneity test; $P < 0.05$ indicated the presence of horizontal pleiotropy. If horizontal pleiotropy was found only based on the MR-PRESSO global heterogeneity test, an MR PRESSO outlier-corrected p-value < 0.05 was considered a significant result. Due to potential differences in allele frequency between populations, MR was applied only to the European ancestry subset of the POAG GWAS meta-analysis and the IOP GWAS meta-analysis that is primarily comprised of European individuals, to avoid confounding by ancestry. Due to this limitation, some of the non-significant MR results for POAG may be due to not having applied MR on the larger, better-powered POAG cross-ancestry GWAS, where in many cases the colocalization was observed. All MR results are summarized in Supplementary Data 29. All MR-related statistical tests were implemented in the following pipeline: https://github.com/segrelabgenomics/TwoSampleMR_pipeline.

Supplementary Note 1

Overview of approach and methods. We applied the following analytical steps to known GWAS loci and to genome-wide association summary statistics of POAG and its major risk factor, IOP, to identify regulatory mechanisms, genes, pathways, and cell types that may significantly contribute to POAG risk. First, to test the relevance of *cis*-eQTLs and *cis*-sQTLs in non-ocular GTEx tissues to POAG and IOP, we tested whether POAG and IOP genetic associations beyond genome-wide significance were enriched among eQTLs and sQTLs in GTEx tissues and retina, using *QTLEnrich*[5,1] (https://github.com/segrelabgenomics/QTLEnrich) that controls for confounding factors. A lower bound number of e/sQTLs proposed to contribute to POAG risk or IOP variation in significant tissues was estimated using an empirical true positive rate estimation approach implemented in *QTLEnrich*[1] (Fig. 1a and Methods). In cases where significant enrichment of GWAS associations among e/sQTLs was found, the target genes of e/sQTLs with top ranked POAG or IOP GWAS p-values (P<0.05) were tested for enrichment in biological

processes using *GeneEnrich*[5] (https://github.com/segrelabgenomics/GeneEnrich), to identify new genetic associations and associated pathways (Fig. 1c). Next, to propose putative causal genes that may underlie genome-wide significant POAG and IOP GWAS loci, we applied two colocalization methods, eCAVIAR[8] and *enloc*[9], to the summary statistics of each known GWAS locus and all overlapping *cis*-eQTLs and *cis*-sQTLs in 49 GTEx tissues (v8)[1] and *cis*-eQTLs in peripheral retina[4] (Fig. 1b and Methods). We applied Mendelian randomization (MR) to the significant colocalization results to further identify a high confidence set of e/sQTLs that may be causal to POAG and/or IOP (Fig. 1b and Methods). We also integrated retina Hi-C and epigenetic data with colocalizing e/sQTLs and POAG loci to provide supportive evidence for potential causal regulatory effects on POAG in retina (Fig. 1b and Methods). We applied gene set enrichment analysis (*GeneEnrich*) to the proposed causal genes based on colocalization analysis and known signaling and metabolic pathways, gene ontologies, and mouse phenotype ontologies (Fig. 1c and Methods). Finally, to identify key pathogenic cell types through which the colocalizing genes may be mediating their effect on POAG and IOP, we applied a method we recently developed, ECLIPSER[23,24] (https://github.com/segrelabgenomics/ECLIPSER) to the GWAS-e/sQTL colocalizing genes (Fig. 1d and Methods) and single-nucleus expression data from glaucoma-relevant ocular tissues: six tissues from the anterior segment[25], including the outflow pathways, retina[25,26], and the optic nerve head, optic nerve, and surrounding posterior tissues[27]. ECLIPSER tests whether the expression of genes mapped to GWAS loci is enriched in specific cell types compared to a null distribution of GWAS loci associated with thousands of traits unrelated to the tissue of interest (Methods). To corroborate the cell type enrichment results, we applied two regression-based methods that assess cell type-specificity for complex traits considering all genetic associations genome-wide: stratified-LD score regression (LDSC)[28] (v1.0.1; https://github.com/bulik/ldsc) and MAGMA[29] (v1.10, https://ctg.cncr.nl/software/magma) (Fig. 1e and Methods).

Supplementary Note 2

Enrichment of POAG and IOP associations among e/sQTLs. As the GWAS loci discovered to date for POAG and IOP explain only a small proportion of the traits' heritability, we tested whether eQTLs and sQTLs can help identify new trait associations with modest effects beyond genome-wide significance, not yet detected due to insufficient GWAS sample sizes (Fig. 1a). To this end, we tested whether *cis*-eQTLs and *cis*-sQTLs from 49 GTEx tissues (v8)[1] and peripheral retina eQTLs[4] were enriched for multiple POAG or IOP associations (GWAS $P<0.05$) using

QTLEnrich[1,5], which adjusts for confounding factors and tissue sample size (Methods). In cases where enrichment was found, we estimated the number of e/sQTLs that may be true associations with POAG or IOP over noise based on an empirical estimation of the true positive rate (π_1), which we implemented in *QTLEnrich*[1] (see Methods). We found significant enrichment of POAG and IOP associations among both eQTLs and sQTLs in most of the 49 GTEx tissues and retina (Bonferroni-corrected $P<5x10^{-4}$) with stronger enrichment found for IOP compared to POAG (Supplementary Data 1-3; One-sided Wilcoxon rank sum test $P<1.6x10^{-8}$). For POAG cross-ancestry, the strongest enrichment among eQTLs was found in brain, lymphocyte, cells cultured fibroblasts, esophagus, and artery (Fig. 2a and Supplementary Data 2), and among sQTLs in kidney cortex, heart atrial appendage, brain, glands, and adipose (Supplementary Figs. 1-2 and Supplementary Data 3). For IOP, the strongest enrichment among eQTLs was found in brain, adipose, skin, fibroblast, and artery (Fig. 2b and Supplementary Data 2), and among sQTLs in brain, glands, and heart atrial appendage (Supplementary Fig. 2 and Supplementary Data 3). eQTLs in retina were also enriched for POAG and IOP associations ($P<1x10^{-5}$), though was not among the top-ranking tissues. While the GTEx tissues are non-ocular tissues, many of the most strongly enriched tissues share cell types that may be pathogenic to glaucoma based on prior knowledge and our GWAS cell type enrichment analysis (Figs. 6-8 and Supplementary Data 35, 45-46). For example, macroglial cells in brain may share e/sQTLs also present in astrocytes or Müller glia cells in the retina, vasculature cells in artery may be a proxy for the lymphatic vessels in the aqueous outflow pathway, and cultured fibroblasts may share genetic programs with the fibroblast cells in the ocular outflow pathways. In all, this analysis suggests that over a thousand common variants that affect gene expression or alternative splicing are likely to contribute to POAG risk and IOP variation.

Supplementary Note 3

Pathway analysis of e/sQTL target genes of top ranked POAG and IOP associations. To identify true positive POAG and IOP associations beyond genome-wide significance, over noise, amongst the enriched e/sQTLs, and to propose potentially new causal genes and pathways, we searched for target genes of e/sQTLs with POAG or IOP GWAS P-values below 0.05 that were enriched in biological pathways, gene ontologies, or mouse phenotype ontologies. Our underlying hypothesis was that genes that contribute to complex traits will cluster in a limited set of biological processes. We applied *GeneEnrich*[5] (Methods) to a selected set of tissues per trait that displayed the highest adjusted fold-enrichment (Supplementary Data 2-3), controlling for gene expression

levels in the given tissue. We found significant enrichment of target genes of brain hippocampus sQTLs with top ranked POAG cross-ancestry associations (GWAS P<0.05) in mitochondrial respiratory complex I (FDR<0.05), including NADH dehydrogenase complex assembly and mitochondrial respiratory chain complex assembly, which included newly proposed genes for POAG (e.g., *NDUFB2, NDUFB4, NDUFAF1, NDUFAF2, NDUFAF6,* and *ATP5SL*), and the citric acid TCA cycle (FDR=0.09) (Supplementary Data 4). Target genes of gastroesophageal junction eQTLs with top ranked POAG associations were enriched (FDR<0.1) in metabolic processes, including glycosaminoglycan biosynthesis keratan sulfate and limonene and pinene degradation. Target genes of brain spinal cord eQTLs enriched for IOP associations were enriched in cellular amino acid catabolic metabolism, in particular, histidine metabolism (FDR=0.03), and skin eQTLs with top ranked IOP associations were enriched in dynactin binding that is involved in moving membrane vesicles along microtubules (FDR=0.07) (Supplementary Data 5). This analysis identified novel genes that may influence POAG risk or IOP via regulatory effects (listed in Supplementary Data 4-5), functioning primarily in metabolic processes. Examining other types of functional relationships, such as co-regulation or protein-protein interactions, might help identify additional glaucoma associations and genes.

Supplementary Note 4

Noncoding genes proposed as contributors to POAG risk. While the majority of colocalizing e/sGenes are protein coding (60-72% per trait), noncoding RNA genes make up 18-20% of the proposed causal genes for POAG and IOP loci (Fig. 2f and Supplementary Data 17), half of which are lincRNAs and half antisense genes. Noncoding genes were proposed as a sole potential causal gene for 10 POAG or IOP loci, including the lincRNA *LINC01948* in POAG cross-ancestry locus rs112142644 on chromosome 5, and *RP11-217B7.2*, a lnc-ABCA1-1:1 lincRNA, in the shared POAG and IOP locus on chromosome 10 (rs2472494 and rs2472493, respectively) (Supplementary Data 13). We further found that e/sQTLs acting on 14 pairs of protein coding genes and their corresponding antisense gene colocalized in the same GWAS locus, including *HLA-F* and *HLA-F-AS1* with POAG risk, *LPP* and *LPP-AS2* with IOP levels, and *MAPT* and *MAPT-AS1* with both POAG and IOP (Supplementary Data 18).

Supplementary Note 5

Secondary signal found in Myocilin locus for POAG. The POAG cross-ancestry GWAS association with the largest odds ratio (OR=5.47) rs74315329 (chr1:171636338:G:A), a nonsense mutation (p.Gln368Ter) in *MYOC*, is European-specific. Its minor allele frequency in non-Finnish European in gnomAD is 0.00126, in African American is 0.00039, and in East and South Asian populations is less than 0.0001 based on gnomAD (https://gnomad.broadinstitute.org/). We tested all e/sQTL gene-tissue pairs that overlapped this locus targeting 24 genes identified an sQTL (chr1:172398537:A:G) acting on *PIGC,* phosphatidylinositol glycan anchor biosynthesis class C, in spleen with eCAVIAR (CLPP=0.12) or artery tissue with *enloc* (artery tibial, RCP=0.34; heart atrial appendage RPC=0.26) that significantly colocalized with the POAG cross-ancestry locus (splicing event: chr1:172370311-172376554; Supplementary Data 13 and Supplementary Fig. 13). Since the nonsense variant in *MYOC* is likely the primary causal variant in the locus, we tested whether a secondary haplotype may exist in the locus and, if so, if it colocalizes with the *PIGC* sQTL. We performed association testing of all variants in the GWAS locus LD interval conditioning on the lead POAG variant rs74315329 using COJO (https://yanglab.westlake.edu.cn/software/gcta/; Methods), and found two low frequency, intronic variants that passed genome-wide significance (chr1:171531082:A:G EUR MAF=0.0036 and chr1:170716994:A:C EUR MAF=0.002) and another LD-independent signal (chr1:172398537:A:G, POAG GWAS conditional $P=1.08 \times 10^{-6}$, EUR MAF=0.075 in gnomAD) that passed Bonferroni correction for the number of variants tested in the locus ($P<5.87 \times 10^{-6}$) (Supplementary Data 24). We next tested whether the residuals of this secondary signal, which are independent of the locus' lead variant rs74315329, colocalized with any of the e/sQTL-tissue pairs in the locus using eCAVIAR and *enloc*. We found suggestive significance (CLPP=0.009) for the POAG LD-independent signal chr1:172398537:A:G and the same *PIGC* sQTL in spleen as above (chr1:172370311-172376554), as well as for a *PRRX1* eQTL (CLPP=0.01) with eCAVIAR (Supplementary Data 25-26), and significant colocalization for the same *PIGC* sQTL in several tissues with *enloc* (maximum RCP=0.13) (Supplementary Data 27-28). *Enloc* also found significant POAG colocalization for another sQTL acting on *PIGC* (max RCP=0.22, chr1:172376636-17237813) and for *PIGC* eQTLs (RCP=0.14) in several tissues (Supplementary Data 27-28). These results suggest that decreased exon 2 skipping in *PIGC* (Supplementary Fig. 13) or increased *PIGC* expression may lead to increased POAG risk.

Supplementary Note 6

Enrichment of POAG genes in retinal macroglial cells is IOP-independent. While the expression of POAG cross-ancestry colocalizing e/sGenes were significantly enriched in astrocytes and Müller glia cells in retina (FDR<0.04; Supplementary Data 35, Fig. 8a and Supplementary Fig. 16a), IOP genes were only nominally enriched in astrocytes (P=0.032; Supplementary Fig. 16c). We assessed the extent to which the enrichment of POAG genes in retinal astrocytes was driven by IOP-independent associations. Only one third of the 12 POAG genes driving astrocyte enrichment in retina were common with IOP (*DGKG, FMNL2, GAS7, LPP*) (Supplementary Fig. 16d,f). Furthermore, when applying ECLIPSER to POAG-only or IOP-only loci or shared loci, significant enrichment (FDR<0.08) was found in astrocytes and Müller glia cells in retina and macula solely for POAG-only and shared loci, but not for IOP-only loci (Supplementary Data 38 and Supplementary Fig. 17), suggesting an IOP-independent effect of astrocytes and Müller glia cells on glaucoma. The POAG genes driving the enrichment in astrocytes were nominally enriched in diacylglycerol metabolic process ($P=1.8x10^{-4}$), negative regulation of fat cell differentiation ($P=7.7x10^{-4}$) and regulation of hematopoietic stem cell differentiation ($P=1.6x10^{-3}$) (Methods and Supplementary Data 37).

Supplementary Note 7

Cell type enrichment analysis of negative control traits for glaucoma. To rule out the possibility that some of the cell type enrichment signals are due to unaccounted confounding factors, we applied ECLIPSER to eight ocular and non-ocular diseases and traits unrelated to glaucoma (listed in Supplementary Data 42). The negative control traits showed enrichment in relevant cell types, such as pigmented cells for eye color, lens in the anterior segment for cataract, retinal pigment epithelium (RPE) in the macula and vascular cells in the ONH for AMD, immune cells for asthma and atopic dermatitis, and melanocytes for melanoma (Supplementary Data 43, Fig. 6a and Supplementary Fig. 21). Since macroglial and microglial cells have been suggested to be transcriptionally reactive to single cell dissociation protocols, more so than to single nuclei dissociation protocols[24,30,31], we evaluated the enrichment in these cell types among the negative control traits. No significant enrichment was found in retinal astrocytes or Müller Glia cells in any of the eight traits. AMD and atopic dermatitis showed nominal or FDR<0.1 enrichment, respectively, in astrocytes only in the macula single-nucleus dataset, but not in the retina dataset. Furthermore, we tested whether cell abundance may have an effect on cell type enrichment

results. The ECLIPSER cell type enrichment significance (p-value) for the POAG cross-ancestry, POAG European, IOP, VCDR, machine learning-based VCDR, central corneal thickness, and corneal hysteresis GWAS did not correlate with cell count per cell type in any of the four single-nucleus datasets (Pearson $R^2<0.2$, $P>0.12$; Supplementary Data 44).

Supplementary Discussion

Limitations to Bayesian colocalization methodologies. There are several limitations to Bayesian-based colocalization analysis[12,32]. Under-estimation of enrichment of e/sQTLs in a given tissue in GWAS hits, used as a prior in the colocalization analysis, or GWAS loci with modest effect sizes may lead to loss of power[12]. Furthermore, the choice of genotype reference panel used to compute LD for fine-mapping of the GWAS and QTL loci may have an effect on colocalization sensitivity. We used GTEx as our reference panel, whose European to African sample ratio is comparable to that of the POAG cross-ancestry GWAS, but has substantially fewer East Asian samples[1]. We do not expect this to have a significant impact on our colocalization results as most of the POAG loci were found to be shared across populations[2], and the fraction of loci with significant colocalization results was similar between the POAG cross-ancestry and European subset GWAS meta-analyses. While colocalization analysis identifies GWAS and e/sQTL signals tagging the same causal variant/haplotype, which suggests that change in expression or splicing of the QTL target gene may be causal to the GWAS trait (vertical pleiotropy), we cannot rule out the possibility that the causal effect on POAG or IOP may be through another mechanism other than change in gene expression or alternative splicing (horizontal pleiotropy). We thus applied Mendelian randomization[12,33,14,34] to all significantly colocalizing e/sQTLs and GWAS loci to test for horizontal pleiotropy and provide additional support for a causal relationship between the colocalizing e/sQTLs and corresponding GWAS loci. Of note, some of the non-significant MR results for POAG may be due to a limitation of two sample MR that requires the e/sQTL and GWAS studies to be from similar population backgrounds, and hence could not be run on the larger and better-powered POAG cross-ancestry GWAS. Also, horizontal pleiotropy could not be tested for e/sQTL-GWAS loci (41.7% of e/sGenes) with less than three LD-independent e/sVariants (instrumental variables) that were selected for the two sample MR analysis. Finally, future inspection of e/sQTLs computed in additional ocular tissues relevant to POAG pathogenicity, such as the outflow pathway and optic nerve head, will help hone in on the genes and regulatory mechanisms contributing to POAG risk.

Supplementary References

1. GTEx Consortium. The GTEx Consortium atlas of genetic regulatory effects across human tissues. *Science* **369**, 1318–1330 (2020).
2. Gharahkhani, P. *et al.* Genome-wide meta-analysis identifies 127 open-angle glaucoma loci with consistent effect across ancestries. *Nat. Commun.* **12**, 1258 (2021).
3. Khawaja, A. P. *et al.* Genome-wide analyses identify 68 new loci associated with intraocular pressure and improve risk prediction for primary open-angle glaucoma. *Nature Genetics* vol. 50 778–782 Preprint at https://doi.org/10.1038/s41588-018-0126-8 (2018).
4. Ratnapriya, R. *et al.* Retinal transcriptome and eQTL analyses identify genes associated with age-related macular degeneration. *Nat. Genet.* **51**, 606–610 (2019).
5. Gamazon, E. R. *et al.* Using an atlas of gene regulation across 44 human tissues to inform complex disease- and trait-associated variation. *Nat. Genet.* **50**, 956–967 (2018).
6. Storey, J. D. & Tibshirani, R. Statistical significance for genomewide studies. *Proc. Natl. Acad. Sci. U. S. A.* **100**, 9440–9445 (2003).
7. Gamazon, E. R., Huang, R. S., Dolan, M. E., Cox, N. J. & Im, H. K. Integrative genomics: quantifying significance of phenotype-genotype relationships from multiple sources of high-throughput data. *Front. Genet.* **3**, 202 (2012).
8. Hormozdiari, F. *et al.* Colocalization of GWAS and eQTL Signals Detects Target Genes. *Am. J. Hum. Genet.* **99**, 1245–1260 (2016).
9. Wen, X., Pique-Regi, R. & Luca, F. Integrating molecular QTL data into genome-wide genetic association analysis: Probabilistic assessment of enrichment and colocalization. *PLoS Genet.* **13**, e1006646 (2017).
10. Barbeira, A. N. *et al.* Exploiting the GTEx resources to decipher the mechanisms at GWAS loci. *Genome Biol.* **22**, 49 (2021).
11. Pividori, M. *et al.* PhenomeXcan: Mapping the genome to the phenome through the transcriptome. *Sci. Adv.* **6**, eaba2083 (2020).
12. Hukku, A. *et al.* Probabilistic colocalization of genetic variants from complex and molecular traits: promise and limitations. *Am. J. Hum. Genet.* **108**, 25–35 (2021).
13. Brown, A. A. *et al.* Predicting causal variants affecting expression by using whole-genome sequencing and RNA-seq from multiple human tissues. *Nat. Genet.* **49**, 1747–1751 (2017).
14. Zuber, V. *et al.* Combining evidence from Mendelian randomization and colocalization: Review and comparison of approaches. *Am. J. Hum. Genet.* **109**, 767–782 (2022).

15. Smith, G. D. & Ebrahim, S. “Mendelian randomization”: can genetic epidemiology contribute to understanding environmental determinants of disease? *Int. J. Epidemiol.* **32**, 1–22 (2003).
16. 1000 Genomes Project Consortium *et al.* A global reference for human genetic variation. *Nature* **526**, 68–74 (2015).
17. Purcell, S. *et al.* PLINK: a tool set for whole-genome association and population-based linkage analyses. *Am. J. Hum. Genet.* **81**, 559–575 (2007).
18. Burgess, S., Small, D. S. & Thompson, S. G. A review of instrumental variable estimators for Mendelian randomization. *Stat. Methods Med. Res.* **26**, 2333–2355 (2017).
19. Burgess, S., Butterworth, A. & Thompson, S. G. Mendelian randomization analysis with multiple genetic variants using summarized data. *Genet. Epidemiol.* **37**, 658–665 (2013).
20. Bowden, J., Davey Smith, G., Haycock, P. C. & Burgess, S. Consistent Estimation in Mendelian Randomization with Some Invalid Instruments Using a Weighted Median Estimator. *Genet. Epidemiol.* **40**, 304–314 (2016).
21. Bowden, J., Davey Smith, G. & Burgess, S. Mendelian randomization with invalid instruments: effect estimation and bias detection through Egger regression. *Int. J. Epidemiol.* **44**, 512–525 (2015).
22. Verbanck, M., Chen, C.-Y., Neale, B. & Do, R. Detection of widespread horizontal pleiotropy in causal relationships inferred from Mendelian randomization between complex traits and diseases. *Nat. Genet.* **50**, 693–698 (2018).
23. Rouhana, J., J. Wang, G. Eraslan, S. Anand, A. Hamel, B. Cole, A. Regev, F. Aguet, K. Ardlie, and A. V. Segrè. ECLIPSER: identifying causal cell types and genes for complex traits through single cell enrichment of e/sQTL-mapped genes in GWAS loci. *BioRxiv* (2021) doi:10.1101/2021.11.24.469720.
24. Eraslan, G. *et al.* Single-nucleus cross-tissue molecular reference maps toward understanding disease gene function. *Science* **376**, eabl4290 (2022).
25. van Zyl, T. *et al.* Cell atlas of the human ocular anterior segment: Tissue-specific and shared cell types. *Proc. Natl. Acad. Sci. U. S. A.* **119**, e2200914119 (2022).
26. Liang, Q. *et al.* A multi-omics atlas of the human retina at single-cell resolution. *Cell Genom.* **3**, 100298 (2023).
27. Monavarfeshani, A. *et al.* Transcriptomic analysis of the ocular posterior segment completes a cell atlas of the human eye. *Proc. Natl. Acad. Sci. U. S. A.* **120**, e2306153120 (2023).
28. Finucane, H. K. *et al.* Heritability enrichment of specifically expressed genes identifies disease-relevant tissues and cell types. *Nat. Genet.* **50**, 621–629 (2018).

29. Watanabe, K., Umićević Mirkov, M., de Leeuw, C. A., van den Heuvel, M. P. & Posthuma, D. Genetic mapping of cell type specificity for complex traits. *Nat. Commun.* **10**, 3222 (2019).
30. Todd, L. *et al.* Reactive microglia and IL1β/IL-1R1-signaling mediate neuroprotection in excitotoxin-damaged mouse retina. *J. Neuroinflammation* **16**, 118 (2019).
31. Denisenko, E. *et al.* Systematic assessment of tissue dissociation and storage biases in single-cell and single-nucleus RNA-seq workflows. *Genome Biol.* **21**, 130 (2020).
32. Wallace, C. Eliciting priors and relaxing the single causal variant assumption in colocalisation analyses. *PLoS Genet.* **16**, e1008720 (2020).
33. Zhu, Z. *et al.* Integration of summary data from GWAS and eQTL studies predicts complex trait gene targets. *Nat. Genet.* **48**, 481–487 (2016).
34. Hemani, G., Bowden, J. & Davey Smith, G. Evaluating the potential role of pleiotropy in Mendelian randomization studies. *Hum. Mol. Genet.* **27**, R195–R208 (2018).
