# Supplementary material for: Integrating genetic regulation and single-cell expression with GWAS prioritizes causal genes and cell types for glaucoma
Source: Nat Commun. 2024 Jan 9;15:396. doi: 10.1038/s41467-023-44380-y (PMC10776627; doi:10.1038/s41467-023-44380-y)
Supplement: Supplementary file 3 — Description of Additional Supplementary Files [file 41467_2023_44380_MOESM3_ESM.pdf]

Description of Additional Supplementary Files

Supplementary Data legends

File Name: Supplementary Data 1. Summary statistics for enrichment analysis of POAG and IOP associations among GTEx and retina e/sQTLs using *QTLEnrich*.
Description: Summary statistics of *QTLEnrich* analysis (Methods) of POAG cross-ancestry GWAS meta-analysis, the POAG European (EUR) subset meta-analysis, and the IOP EUR GWAS meta-analysis, and expression and splicing quantitative trait loci (eQTLs and sQTLs) from 49 GTEx tissues and eQTLs from peripheral retina. Enrichment of genetic associations with GWAS P-value below 0.05 among the e/sQTL sets at FDR<0.05 are assessed with an adjusted fold-enrichment, empirically estimated true positive rate, Pi1, of trait associations amongst significant best e/sQTLs per e/sGene (FDR<0.05), and estimated number of trait associations among e/sQTLs. Bonferroni corrected p-value ($P<5x10^{-4}$), adjusting for number of tissues and e/sQTL type tested per GWAS, was used to determine significant tissues whose e/sQTLs are enriched for GWAS associations.

File Name: Supplementary Data 2. *QTLEnrich* results for POAG and IOP GWAS meta-analyses and eQTLs from 49 GTEx tissues and retina.
Description: *QTLEnrich* results for POAG cross-ancestry GWAS meta-analysis, POAG European (EUR) subset meta-analysis, and IOP GWAS meta-analysis, testing whether the best expression quantitative trait loci (eQTL) per eGene (FDR<0.05) set from each of the 49 GTEx tissues and retina is enriched for multiple associations with the given trait. *QTLEnrich* is a one-sided test (Methods). Significant tissues were determined, correcting for 50 tissues and two QTL types tested, using Bonferroni correction (*QTLEnrich* Enrichment P-value < $5x10^{-4}$).

File Name: Supplementary Data 3. *QTLEnrich* results for POAG and IOP GWAS meta-analyses and sQTLs from 49 GTEx tissues.
Description: *QTLEnrich* results for POAG cross-ancestry GWAS meta-analysis, POAG European (EUR) subset meta-analysis, and IOP GWAS meta-analysis and splicing quantitative trait loci (sQTLs), testing whether the best sQTL per sGene (FDR<0.05) set from each of the 49 GTEx tissues is enriched for multiple associations with the given trait. *QTLEnrich* is a one-sided test (Methods). Significant tissues were determined, correcting for 50 tissues and two QTL types tested, using Bonferroni correction (*QTLEnrich* Enrichment P-value < $5x10^{-4}$).

File Name: Supplementary Data 4. Gene set enrichment analysis of target genes of e/sQTLs with top ranked POAG GWAS P-values using *GeneEnrich*.
Description: The target genes of eQTLs or sQTLs with GWAS P-value below 0.05 in the POAG cross-ancestry meta-analysis or POAG European (EUR) subset were tested for enrichment in biological pathways and gene ontologies using *GeneEnrich*, a hypergeometric-based test (one-sided; Methods). Gene sets from the following resources were tested: Gene Ontology (GO) biological processes, molecular function, and cellular components, Reactome, Kyoto Encyclopedia of Genes and Genomes (KEGG), and mouse phenotype ontology from the Mouse Genome Informatics (MGI). *GeneEnrich* was applied to a selected set of tissues per trait that displayed the highest adjusted fold-enrichment, controlling for gene expression levels in the given tissue. Results are shown for gene sets with empirical gene-set enrichment p-values below 0.05. Multiple hypothesis correction was determined at Benjamini-Hochberg false discovery rate (FDR) < 0.1, computed per resource.

File Name: Supplementary Data 5. Gene set enrichment analysis of target genes of e/sQTLs with top ranked IOP GWAS P-values using *GeneEnrich*.

Description: The target genes of eQTLs or sQTLs with GWAS P-value below 0.05 in the largest to date IOP meta-analysis were tested for enrichment in biological pathways and gene ontologies using *GeneEnrich*, a hypergeometric-based test (one-sided; Methods). Gene sets from the following resources were tested: Gene Ontology (GO) biological processes, molecular function, and cellular components, Reactome, Kyoto Encyclopedia of Genes and Genomes (KEGG), and mouse phenotype ontology from the Mouse Genome Informatics (MGI). *GeneEnrich* was applied to a selected set of tissues per trait that displayed the highest adjusted fold-enrichment, controlling for gene expression levels in the given tissue. Results are shown for gene sets with empirical gene-set enrichment p-values below 0.05. Multiple hypothesis correction was determined at Benjamini-Hochberg false discovery rate (FDR) < 0.1, computed per resource.

File Name: Supplementary Data 6. List of genome-wide significant POAG and IOP GWAS loci.
Description: This table contains the list of genome-wide significant (P<5E-08) POAG and IOP GWAS lead variants whose loci were subjected to colocalization, fine-mapping, and Mendelian randomization analysis. This list included 127 GWAS loci from the cross-ancestry POAG GWAS meta-analysis of 34,179 cases and 349,321 controls (Gharahkhani, P. *et al.* Nat. Comm. 2021), 68 GWAS loci from the European subset GWAS meta-analysis of 16,677 POAG cases and 199,580 controls (Gharahkhani, P. *et al.* Nat. Comm. 2021), and 133 LD-independent GWAS variants in 112 loci from the IOP GWAS meta-analysis of 139,555 primarily UK Biobank (European) samples (Khawaja, A *et al.*, Nature Genetics 2018).

File Name: Supplementary Data 7. eCAVIAR colocalization results for POAG cross-ancestry GWAS loci.
Description: This table contains the significant eCAVIAR colocalization results for all POAG cross-ancestry GWAS variants by QTL type, target gene and tissue combinations tested across 49 GTEx tissues and peripheral retina. Significance was determined at a colocalization posterior probability (CLPP) > 0.01. To filter out potential false positives, we flagged cases where the GWAS p-value of the colocalization e/sVariant was above 10^{-5} or whose e/sQTL p-values was above 10^{-4} and/or did not pass FDR below 0.05 (FALSE in Column: 'Pass_QC_QTL_FDR05_P1E04_GWAS_P1E05'). All e/sVariants within an LD interval (r^2>0.1 plus 50kb on either side) around each lead GWAS variant were tested in the colocalization analysis.

File Name: Supplementary Data 8. eCAVIAR colocalization results for POAG European GWAS loci.
Description: This table contains the significant eCAVIAR colocalization results for all POAG European GWAS variants by QTL type, target gene and tissue combinations tested across 49 GTEx tissues and peripheral retina. Significance was determined at a colocalization posterior probability (CLPP) > 0.01. To filter out potential false positives, we flagged cases where the GWAS p-value of the colocalization e/sVariant was above 10^{-5} or whose e/sQTL p-values was above 10^{-4} and/or did not pass FDR below 0.05 (FALSE in Column: 'Pass_QC_QTL_FDR05_P1E04_GWAS_P1E05'). All e/sVariants within an LD interval (r^2>0.1 plus 50kb on either side) around each lead GWAS variant were tested in the colocalization analysis.

File Name: Supplementary Data 9. eCAVIAR colocalization results for IOP GWAS loci.
Description: This table contains the significant eCAVIAR colocalization results for all IOP GWAS variants by QTL type, target gene and tissue combinations tested across 49 GTEx tissues and peripheral retina. Significance was determined at a colocalization posterior probability (CLPP) > 0.01. To filter out potential false positives, we flagged cases where the GWAS p-value of the colocalization e/sVariant was above 10^{-5} or whose e/sQTL p-values was above 10^{-4} and/or did

not pass FDR below 0.05 (FALSE in Column: 'Pass_QC_QTL_FDR05_P1E04_GWAS_P1E05'). All e/sVariants within an LD interval (r^2>0.1 plus 50kb on either side) around each lead GWAS variant were tested in the colocalization analysis.

File Name: Supplementary Data 10. *enloc* colocalization results for POAG cross-ancestry GWAS loci.
Description: This table contains the significant *enloc* colocalization results from all POAG cross-ancestry GWAS variants by QTL type, target gene and tissue combinations tested across 49 GTEx tissues. Significance was determined at a regional colocalization probability (RCP) > 0.1. The probability that a variant may be causal to the GWAS trait, posterior inclusion probability (PIP), was computed with DAP-G and provided for each significantly colocalizing e/sVariant (QTL PIP). To filter out potential false positives, we flagged cases where the GWAS p-value of the colocalization e/sVariant was above 10^{-5} or whose e/sQTL p-values was above 10^{-4} and/or did not pass FDR below 0.05 (FALSE in Column: 'Pass_QC_QTL_FDR05_P1E04_GWAS_P1E05'). All e/sVariants within an LD interval (r^2>0.1 plus 50kb on either side) around each lead GWAS variant were tested in the colocalization analysis.

File Name: Supplementary Data 11. *enloc* colocalization results for POAG European GWAS loci.
Description: This table contains the significant *enloc* colocalization results from all POAG European GWAS variants by QTL type, target gene and tissue combinations tested across 49 GTEx tissues. Significance was determined at a regional colocalization probability (RCP) > 0.1. The probability that a variant may be causal to the GWAS trait, posterior inclusion probability (PIP), was computed with DAP-G and provided for each significantly colocalizing e/sVariant (QTL PIP). To filter out potential false positives, we flagged cases where the GWAS p-value of the colocalization e/sVariant was above 10^{-5} or whose e/sQTL p-values was above 10^{-4} and/or did not pass FDR below 0.05 (FALSE in Column: 'Pass_QC_QTL_FDR05_P1E04_GWAS_P1E05'). All e/sVariants within an LD interval (r^2>0.1 plus 50kb on either side) around each lead GWAS variant were tested in the colocalization analysis.

File Name: Supplementary Data 12. *enloc* colocalization results for IOP GWAS loci.
Description: This table contains the significant *enloc* colocalization results from all IOP GWAS variants by QTL type, target gene and tissue combinations tested across 49 GTEx tissues. Significance was determined at a regional colocalization probability (RCP) > 0.1. The probability that a variant may be causal to the GWAS trait, posterior inclusion probability (PIP), was computed with DAP-G and provided for each significantly colocalizing e/sVariant (QTL PIP). To filter out potential false positives, we flagged cases where the GWAS p-value of the colocalization e/sVariant was above 10^{-5} or whose e/sQTL p-values was above 10^{-4} and/or did not pass FDR below 0.05 (FALSE in Column: 'Pass_QC_QTL_FDR05_P1E04_GWAS_P1E05'). All e/sVariants within an LD interval (r^2>0.1 plus 50kb on either side) around each lead GWAS variant were tested in the colocalization analysis.

File Name: Supplementary Data 13. Summary of eCAVIAR and *enloc* colocalization results for POAG and IOP GWAS loci.
Description: This table contains a summary of the significant eCAVIAR and *enloc* colocalization results for each of the 127 cross-ancestry POAG, 68 European (EUR) POAG, and 133 IOP GWAS loci listed in Supplementary Data 1. Results are shown based on eCAVIAR and *enloc* separately, the unique union of the colocalizing e/sGenes across both methods, and the intersection of colocalizing e/sGenes between both methods. The direction of effect of each colocalizing e/sQTL relative to POAG risk or IOP levels is given as '+' or '-' in square brackets. The number preceding the +/- signs specifies the number of tissues in which the eQTL (e) or sQTL (s) has the given

direction of effect on the trait. Significance was determined at colocalization posterior probability (CLPP) > 0.01 for eCAVIAR and regional colocalization probability (RCP) > 0.1 for *enloc*.

File Name: Supplementary Data 14. Number of POAG and IOP GWAS loci with colocalizing eQTLs and/or sQTLs.
Description: This table summarizes the number and percentage of POAG cross-ancestry, POAG European (EUR), and IOP GWAS loci with at least one colocalizing eQTLs, at least one colocalizing sQTLs or at least one colocalizing eQTLs and/sQTLs based on the eCAVIAR or enloc methods. The summary statistics are also shown for the unique union of colocalizing e/sGenes across both methods, eCAVIAR and enloc, and the intersection of colocalizing e/sGenes between the methods. Significance was determined at colocalization posterior probability (CLPP) > 0.01 for eCAVIAR and regional colocalization probability (RCP) > 0.1 for *enloc*.

File Name: Supplementary Data 15. Significant e/sQTL colocalization with both eCAVIAR and *enloc* for POAG and IOP GWAS loci.
Description: This table contains a list of all eGenes and/or sGenes whose e/sQTLs significantly colocalized with POAG cross-ancestry GWAS loci, POAG European subset loci, and IOP loci with both eCAVIAR and *enloc*. Significance was determined at colocalization posterior probability (CLPP) > 0.01 for eCAVIAR and regional colocalization probability (RCP) > 0.1 for *enloc*. This is a subset of the rows and columns listed in Supplementary Data 13. All the listed e/sGenes had significant results based on two-sample Mendelian randomization (MR) analysis of the e/sQTLs and POAG and/or IOP GWAS loci (results in Supplementary Data 29). The last three columns specify if the colocalizing e/sGenes for the given GWAS also colocalized with the two other GWAS.

File Name: Supplementary Data 16. Number of e/sGenes that colocalized per POAG or IOP GWAS locus.
Description: This table contains the average number of eGenes or sGenes whose eQTLs or sQTLs, respectively, colocalized with any of the 127 cross-ancestry POAG loci, 68 European (EUR) POAG loci, and 133 IOP loci (listed in Supplementary Data 1) and their standard error (s.e.), based on eCAVIAR or *enloc*. These values are also shown for the unique union of colocalizing e/sGenes across both methods, eCAVIAR and enloc, and the intersection of colocalizing e/sGenes between the methods. Significance was determined at colocalization posterior probability (CLPP) > 0.01 for eCAVIAR and regional colocalization probability (RCP) > 0.1 for *enloc*.

File Name: Supplementary Data 17. Biotype count of e/sGenes that colocalized with POAG and IOP GWAS loci.
Description: Distribution of protein-coding and non-coding gene biotypes of the eGenes and sGenes whose e/sQTLs significantly colocalized with 76 POAG cross-ancestry GWAS loci, 36 POAG European loci, and 79 IOP loci. Significance was determined at colocalization posterior probability (CLPP) > 0.01 for eCAVIAR and regional colocalization probability (RCP) > 0.1 for *enloc*.

File Name: Supplementary Data 18. Protein-coding and antisense pairs whose e/sQTLs colocalize with POAG or IOP GWAS loci.
Description: List of pairs of protein coding genes and their antisense whose eQTLs and/or sQTLs colocalized with POAG or IOP GWAS associations in the same locus. The direction of effect of each colocalizing e/sQTL relative to POAG risk or IOP levels is given as '+' or '-' in the square brackets in columns M and N. The number preceding the +/- signs specifies the number of tissues in which the eQTL (e) or sQTL (s) has the given direction of effect on the trait. The maximum

CLPP or RCP is also given per eQTL or sQTL across all tissues where significant colocalization was found. Significance was determined at colocalization posterior probability (CLPP) > 0.01 for eCAVIAR and regional colocalization probability (RCP) > 0.1 for *enloc*.

File Name: Supplementary Data 19. Number of POAG and IOP GWAS loci with single colocalizing e/sGene.
Description: This table contains the number and percentage of POAG cross-ancestry GWAS loci, POAG European (EUR) loci, and IOP loci in which only a single eGene or a single sGene colocalized with the GWAS associations, based on eCAVIAR or *enloc*. These numbers are also shown for the unique union of colocalizing e/sGenes across both methods, eCAVIAR and enloc, or considering the intersection of colocalizing e/sGenes between the methods. Significance was determined at colocalization posterior probability (CLPP) > 0.01 for eCAVIAR and regional colocalization probability (RCP) > 0.1 for *enloc*.

File Name: Supplementary Data 20. List of single colocalizing e/sGenes in POAG and IOP GWAS loci.
Description: This table lists the POAG (cross-ancestry and European) and IOP GWAS loci (N=80) with a single eQTL and/or sQTL target gene that colocalized with the locus. The direction of effect of each colocalizing e/sQTL relative to POAG risk or IOP levels is given as '+' or '-' in square brackets. The number preceding the +/- signs specifies the number of tissues in which the eQTL (e) or sQTL (s) has the given direction of effect on the trait. Significance was determined at colocalization posterior probability (CLPP) > 0.01 for eCAVIAR and regional colocalization probability (RCP) > 0.1 for *enloc*.

File Name: Supplementary Data 21. Total number of e/sGenes that colocalized with POAG and IOP GWAS loci.
Description: Number of eGenes and sGenes tested for colocalization with 127 POAG cross-ancestry GWAS loci, 68 POAG European (EUR) loci, and 133 IOP loci and number of e/sGenes whose e/sQTLs significantly colocalized with the POAG and IOP loci. Significance was determined at colocalization posterior probability (CLPP) > 0.01 for eCAVIAR and regional colocalization probability (RCP) > 0.1 for *enloc*.

File Name: Supplementary Data 22. Human retina Hi-C loops, CREs and SEs that intersect with colocalizing e/sQTLs and POAG or IOP GWAS loci.
Description: This table lists and annotates the POAG cross-ancestry GWAS loci whose lead GWAS variants and/or linkage disequilibrium (LD) proxy variants (r^2>0.7) intersect with 3D chromatin conformation capture (Hi-C) loops from non-diseased human retina, and that contain in the GWAS LD region (r^2>0.7) a gene or gene/s whose gene body or transcription start site (TSS) overlaps with a Hi-C foot loop. A target gene for the GWAS locus is identified by Hi-C loops if one foot of the loop overlaps e/sQTL variant/s (FDR<0.05) and the second foot overlaps the gene body or TSS of the e/sQTL target gene. This table also identified POAG cross-ancestry GWAS loci that overlap *cis* regulatory elements (CREs) or super-enhancers (SEs) from epigenetic data from non-diseased human retina. CRE and SE target genes were defined if the e/sVariant and gene body or TSS of the gene overlapped the same CRE or SE. Columns A through L were taken from Supplemental Data 4 in Marchal *et al.,* Nature Communications 2022 (PMID: 36207300). The significantly colocalizing e/sGenes based on eCAVIAR and/or *enloc* are listed per GWAS locus, and we specified if the Hi-C, CRE or SE proposed target gene/s matches the colocalizing e/sGene/s.

File Name: Supplementary Data 23. Colocalization results for European-specific POAG GWAS loci.

Description: This table contains 12 European (EUR)-specific POAG GWAS loci annotated with colocalizing e/sGenes for 49 GTEx tissues and retina with the POAG EUR or IOP GWAS. Significant colocalization was determined at colocalization posterior probability (CLPP) > 0.01 for eCAVIAR and regional colocalization probability (RCP) > 0.1 for *enloc*. EUR-specific POAG GWAS loci were determined based on either not be significant in the cross-ancestry POAG GWAS meta-analysis or displaying allelic heterogeneity in the POAG cross-ancestry meta-analysis.

File Name: Supplementary Data 24. Variant association testing of *MYOC* POAG locus after conditioning on lead GWAS variant.
Description: This is the output from applying conditional analysis of the POAG cross-ancestry GWAS meta-analysis summary statistics on chromosome 1, conditioning on the *MYOC* locus lead variant, rs74315329. The --cojo-cond command in the GCTA tool suite (https://yanglab.westlake.edu.cn/software/gcta/) was used. bC, bC_se, and pC are the effect size, standard error and p-value from the conditional analysis of all variants in the LD interval around the GWAS lead variant. b, se, and p are the effect size, standard error, and p-value from the original POAG GWAS, respectively. Variants that passed genome-wide significance (P<5E-08) were considered significant.

File Name: Supplementary Data 25. eCAVIAR colocalization analysis of *MYOC* locus in POAG cross-ancestry GWAS after conditional analysis on lead GWAS variant.
Description: This table lists the significant eCAVIAR colocalization results for the POAG GWAS *MYOC* locus using the cross-ancestry GWAS residual summary statistics conditioned on the lead GWAS variant rs74315329 (chr1:171636338:G:A). The colocalization results are from testing all QTL type, target gene, and tissue combinations across 49 GTEx tissues and peripheral retina. Conditional analysis was performed using --cojo-cond in GCTA (https://yanglab.westlake.edu.cn/software/gcta/). Significance was determined at a colocalization posterior probability (CLPP) > 0.01. To filter out potential false positives, we flagged cases where the GWAS p-value of the colocalization e/sVariant was above $2x10^{-5}$ or whose e/sQTL p-values was above 10^{-4} and/or did not pass FDR below 0.05 (FALSE in Column: 'Pass_QC_QTL_FDR05_P1E04_GWAS_P2E05'). All e/sVariants within an LD interval (r^2>0.1 plus 50kb on either side) around the lead GWAS variant were tested in the colocalization analysis.

File Name: Supplementary Data 26. Significant eCAVIAR results for *MYOC* locus after conditional analysis on lead GWAS variant.
Description: Summary of the significant eCAVIAR colocalization results at colocalization posterior probability (CLPP) > 0.01 for the POAG GWAS *MYOC* locus after conditioning on the lead GWAS variant rs74315329 (chr1:171636338:G:A). The direction of effect of each colocalizing e/sQTL relative to POAG risk is given as '+' or '-' in square brackets. The number preceding the +/- signs specifies the number of tissues in which the eQTL (e) or sQTL (s) has the given direction of effect on the trait.

File Name: Supplementary Table 27. *enloc* colocalization analysis of *MYOC* locus in POAG cross-ancestry GWAS after conditional analysis on lead GWAS variant.
Description: This table lists the significant *enloc* colocalization results for the POAG GWAS *MYOC* locus using the cross-ancestry GWAS residual summary statistics conditioning on the lead GWAS variant rs74315329 (chr1:171636338:G:A). The colocalization results are from testing all QTL type, target gene and tissue combinations across 49 GTEx tissues and peripheral retina. Conditional analysis was performed using --cojo-cond in GCTA (https://yanglab.westlake.edu.cn/software/gcta/). Significance was determined at a regional colocalization probability (RCP) > 0.1. To filter out potential false positives, we flagged cases

where the GWAS p-value of the colocalization e/sVariant was above $2x10^{-5}$ or whose e/sQTL p-values was above 10^{-4} and/or did not pass FDR below 0.05 (FALSE in Column: 'Pass_QC_QTL_FDR05_P1E04_GWAS_P2E05'). The probability that a variant may be causal to the GWAS trait, posterior inclusion probability (PIP), was computed with DAP-G and provided for each significantly colocalizing e/sVariant (QTL PIP). All e/sVariants within an LD interval (r^2>0.1 plus 50kb on either side) around the lead GWAS variant were tested in the colocalization analysis.

File Name: Supplementary Table 28. Significant *enloc* results for *MYOC* locus after conditional analysis on lead GWAS variant that pass QC.
Description: Summary of the significant *enloc* colocalization results at a regional colocalization probability (RCP) above 0.01 for the POAG GWAS *MYOC* locus after conditioning on the lead GWAS variant rs74315329 (chr1:171636338:G:A). The direction of effect of each colocalizing e/sQTL relative to POAG risk is given as '+' or '-' in square brackets. The number preceding the +/- signs specifies the number of tissues in which the eQTL (e) or sQTL (s) has the given direction of effect on the trait.

File Name: Supplementary Data 29. Two sample mendelian randomization of colocalizing e/sQTLs with POAG and IOP European GWAS loci.
Description: Two-sample Mendelian Randomization (MR) was applied to all e/sQTLs (target gene-tissue combinations) that significantly colocalized with genome-wide significant POAG cross-ancestry and European (EUR) GWAS loci or IOP loci to provide additional genetic support for a causal relationship between the colocalizing e/sQTLs and POAG risk and/or IOP levels. For each of these e/sQTLs, MR was applied to the summary statistics only of the European subset POAG GWAS and the IOP primarily European GWAS meta-analysis to avoid confounding by ancestry, since the e/sQTLs are from a majority of European samples. Significant (FDR < 0.05) e/sVariants with P-value < 5×10^{-6} were considered in the MR analysis. If no significant e/sVariants were found at $P < 5 \times 10^{-6}$ for a given e/sGene and tissue, all variants with FDR < 0.05 were selected. The e/sVariants were intersected with the GWAS variants and an LD-independent set of variants ($r^2 < 0.1$) was selected choosing variants with lowest GWAS p-values (LD clumping). If no variants passed this filtering, all variant-gene pair with p-value < 5*10E-6 were selected for the MR analysis. The number of instrumental variable (IV) variants used in the MR analysis is provided. The primary MR test is the Wald ratio test. If multiple variants constitute the instrument for the e/sQTL target gene, the inverse-variance weighted (IVW) method was used to pool variant-specific estimates. For multiple hypothesis correction, Benjamini-Hochberg (BH) FDR calculation was applied to the Wald ratio or IVW p-values of all 6,165 tests and an FDR < 0.05 was considered statistically significant. For sensitivity analysis, the simple-median, weighted-median, MR-Egger, and MR-PRESSO methods were applied. Horizontal pleiotropy was tested using the Egger-intercept test and MR-PRESSO global heterogeneity test (one-sided test); P < 0.05 indicated presence of horizontal pleiotropy.

File Name: Supplementary Data 30. Gene-set enrichment analysis of e/sGenes colocalizing with POAG cross-ancestry GWAS loci.
Description: Genes that significantly colocalized with POAG cross-ancestry GWAS loci (CLPP>0.01 and/or RCP>0.1) were tested for enrichment in over 11,000 gene sets from four databases downloaded from MSigDB, using *GeneEnrich*, a hypergeometric-based test (one-sided; Methods). The databases include Gene Ontology (GO) with three domains: biological processes (BP), molecular function (MF), and cellular components (CC); Reactome; Kyoto Encyclopedia of Genes and Genomes (KEGG); and mouse phenotype ontology gene sets from the Mouse Genome Informatics (MGI). Only gene sets with 10 to 1000 genes were tested, excluding the HLA region. Gene sets with an empirical gene set enrichment p-value (corrected

for null distribution of all genes expressed in the given tissue using permutation analysis) below 0.05 are shown (see Methods). The colocalizing e/sGenes driving the gene set enrichment signal per gene set are listed in the last column. Multiple hypothesis correction was determined at Benjamini-Hochberg false discovery rate (FDR) < 0.1, computed per resource.

File Name: Supplementary Data 31. Gene set enrichment analysis of e/sGenes colocalizing with POAG European GWAS loci.
Description: Genes that significantly colocalized with POAG European GWAS loci (CLPP>0.01 and/or RCP>0.1) were tested for enrichment in over 11,000 gene sets from four databases downloaded from MSigDB, using *GeneEnrich*, a hypergeometric-based test (one-sided; Methods). The databases include Gene Ontology (GO) with three domains: biological processes (BP), molecular function (MF), and cellular components (CC); Reactome; Kyoto Encyclopedia of Genes and Genomes (KEGG); and mouse phenotype ontology gene sets from the Mouse Genome Informatics (MGI). Only gene sets with 10 to 1000 genes were tested, excluding the HLA region. Gene sets with an empirical gene set enrichment p-value (corrected for null distribution of all genes expressed in the given tissue using permutation analysis) below 0.05 are shown (see Methods). The colocalizing e/sGenes driving the gene set enrichment signal per gene set are listed in the last column. Multiple hypothesis correction was determined at Benjamini-Hochberg false discovery rate (FDR) < 0.1, computed per resource.

File Name: Supplementary Data 32. Gene set enrichment analysis of e/sGenes colocalizing with IOP GWAS loci.
Description: Genes that significantly colocalized with IOP GWAS loci (CLPP>0.01 and/or RCP>0.1) were tested for enrichment in over 11,000 gene sets from four databases downloaded from MSigDB, using *GeneEnrich*, a hypergeometric-based test (one-sided; Methods). The databases include Gene Ontology (GO) with three domains: biological processes (BP), molecular function (MF), and cellular components (CC); Reactome; Kyoto Encyclopedia of Genes and Genomes (KEGG); and mouse phenotype ontology gene sets from the Mouse Genome Informatics (MGI). Only gene sets with 10 to 1000 genes were tested, excluding the HLA region. Gene sets with an empirical gene set enrichment p-value (corrected for null distribution of all genes expressed in the given tissue using permutation analysis) below 0.05 are shown (see Methods). The colocalizing e/sGenes driving the gene set enrichment signal per gene set are listed in the last column. Multiple hypothesis correction was determined at Benjamini-Hochberg false discovery rate (FDR) < 0.1, computed per resource.

File Name: Supplementary Data 33. Gene set enrichment analysis of colocalizing POAG and IOP e/sGenes including HLA region.
Description: Genes that significantly colocalized with POAG cross-ancestry, POAG European (EUR) and IOP GWAS loci (CLPP>0.01 and/or RCP>0.1) were tested for enrichment in over 11,000 gene sets from four databases downloaded from MSigDB, using *GeneEnrich*, a hypergeometric-based test (one-sided; Methods), and including the HLA region. The databases include Gene Ontology (GO) with three domains: biological processes (BP), molecular function (MF), and cellular components (CC); Reactome; Kyoto Encyclopedia of Genes and Genomes (KEGG); and mouse phenotype ontology gene sets from the Mouse Genome Informatics (MGI). Only gene sets with 10 to 1000 genes were tested. Gene sets with an empirical gene set enrichment p-value (corrected for null distribution of all genes expressed in the given tissue using permutation analysis) below 0.05 are shown (see Methods). The colocalizing e/sGenes driving the gene set enrichment signal per gene set are listed in the last column. Multiple hypothesis correction was determined at Benjamini-Hochberg false discovery rate (FDR) < 0.1, computed per resource.

File Name: Supplementary Data 34. List of POAG, IOP and related trait GWAS and their number of loci used in cell type enrichment analysis.
Description: List of GWAS related to POAG and the number of GWAS variants at genome-wide significance reported in the corresponding references that were analyzed with ECLIPSER to test for enrichment of genes mapped to the GWAS locus sets based on e/sQTLs in ocular cell type single cell expression. The number of GWAS loci is given after linkage-disequilibrium (LD) clumping (r^2>0.8) and sharing of e/sQTL target genes between lead GWAS variants per trait, and the number of GWAS loci with e/sQTL-mapped genes. The gene mapping for the POAG and IOP traits was based on colocalization analysis and for the other traits based on LD (r^2>0.8) between the lead GWAS variants and the fine-mapped e/sQTLs. VCDR, vertical cup-to-disc ratio; ML, machine learning.

File Name: Supplementary Data 35. Cell type enrichment of e/sQTL-mapped genes for POAG, IOP and related trait GWAS locus sets in eye tissues using ECLIPSER.
Description: Cell type enrichment results of e/sQTL-mapped genes to loci of POAG, IOP and related trait GWAS listed in Supplementary Data 34, using ECLIPSER (one-sided test). Genes were mapped to the POAG and IOP GWAS loci based on colocalization analysis (union of colocalizing e/sGenes in Supplementary Data 13) and to the other trait loci based on LD (r^2>0.8) between the lead GWAS variants and fine-mapped e/sQTLs. Cell type enrichment was applied to differential gene expression analysis of single nucleus RNA-seq data of the anterior segment, macula, peripheral retina (retina), and the optic nerve head, optic nerve, and surrounding posterior tissues, including peripapillary sclera, peripheral sclera, and choroid (OpticNerveHead). Leading edge genes are cell type-specific genes mapped to the GWAS loci whose cell type specificity score was equal to or above the 95th percentile enrichment cutoff. These genes are proposed to influence the given complex trait in the significantly enriched cell type. Cell types with a tissue-wide Benjamini-Hochberg false discovery rate (FDR) below 0.1, correcting for multiple hypothesis testing within a tissue, were considered significantly enriched for genes contributing to the given trait. VCDR, vertical cup-to-disc ratio; ML, machine learning; e, eQTL; s, sQTL.

File Name: Supplementary Data 36. GTEx tissues used as background expression for gene set enrichment analysis of cell type specific e/sGenes colocalizing with POAG or IOP GWAS loci.
Description: List of GTEx tissues used as background expression for the gene set enrichment analysis (in Supplementary Data 37) of cell type-specific e/sGenes that colocalized with POAG or IOP GWAS loci. GTEx (non-ocular) tissues were matched to a selected set of cell types from ocular tissues based on cellular similarity.

File Name: Supplementary Data 37. Gene set enrichment analysis of cell type-specific e/sGenes colocalizing with POAG or IOP GWAS loci.
Description: Gene sets enriched for cell type-specific e/sGenes that colocalized with POAG cross-ancestry (CA), POAG European (EUR) or IOP GWAS loci (from Supplementary Data 13), using *GeneEnrich* (one-sided, hypergeometric-based test), for a select set of eye tissues and cell types based on ECLIPSER analysis (tissue-wide FDR ≤ 0.1). Gene sets with an empirical gene set enrichment p-value (corrected for null distribution of all genes expressed in the given tissue using permutation analysis) below 0.05 are shown (see Methods). For the background sets of genes, we used all genes expressed in a tissue that was most relevant for the enriched cell type from 49 GTEx tissues and retina, excluding the HLA region. Over 11,000 gene sets were tested (between 10-1000 genes per gene set) from four databases downloaded from MSigDB: Gene Ontology (GO): biological processes (BP), molecular function (MF), and cellular components (CC); Reactome; Kyoto Encyclopedia of Genes and Genomes (KEGG); and mouse phenotype ontology gene sets from the Mouse Genome Informatics (MGI). The colocalizing e/sGenes driving the gene set enrichment signal per gene set are listed in the last column. Multiple hypothesis

correction was determined at Benjamini-Hochberg false discovery rate (FDR) < 0.1, computed per gene set resource.

File Name: Supplementary Data 38. Cell type enrichment of e/sQTL-mapped genes for POAG only, IOP only, and shared GWAS loci in eye tissues using ECLIPSER.
Description: Cell type enrichment results of e/sQTL-mapped genes to common and independent POAG and IOP loci, using ECLIPSER (one-sided test). Genes were mapped to POAG and IOP GWAS loci based on colocalization analysis (union of e/sGenes in Supplementary Data 13). Cell type enrichment was applied to differential gene expression analysis of single nucleus RNA-seq data of the anterior segment, macula, peripheral retina (retina), and the optic nerve head, optic nerve, and surrounding posterior tissues, including peripapillary sclera, peripheral sclera, and choroid (OpticNerveHead). Leading edge genes are cell type-specific genes mapped to the GWAS loci whose cell type specificity score was equal to or above the 95th percentile enrichment cutoff. These genes are proposed to influence the complex trait in the significantly enriched cell type. Cell types with a tissue-wide Benjamini-Hochberg false discovery rate (FDR) below 0.1, correcting for multiple hypothesis testing within a tissue, were considered significantly enriched for genes contributing to the given trait. CA, cross-ancestry; EUR, European; e, eQTL; s, sQTL.

File Name: Supplementary Data 39. Summary table of colocalizing e/sGenes and enriched pathways and cell types for POAG cross-ancestry GWAS loci.
Description: Table that summarizes the pathways and cell types in which the eGenes or sGenes that colocalized with the POAG cross-ancestry GWAS loci belong to or are enriched in. The direction of effect of each colocalizing e/sQTL relative to POAG risk is given as '+' or '-' in the square brackets in columns I and J. The number preceding the +/- signs specifies the number of tissues in which the eQTL (e) or sQTL (s) has the given direction of effect on the trait. The maximum CLPP or RCP is also given per e/sQTL across all tissues where significant colocalization was found. Significant colocalization was determined at colocalization posterior probability (CLPP) > 0.01 for eCAVIAR and regional colocalization probability (RCP) > 0.1 for *enloc*. 'most_significant_gene_set_membership' contains the gene set/s from Gene Ontology (GO), Reactome, or KEGG with the most significant *GeneEnrich* empirical gene set enrichment p-value (from Supplementary Data 30) that contain the colocalizing gene. 'pass_nominal_significance_gene_sets' refers to gene set/s that are nominally enriched (*GeneEnrich* Empirical P<0.05) for colocalizing genes with POAG cross-ancestry GWAS loci and that contain the given colocalizing gene. 'pass_bh_significance_gene_sets' refers to gene set/s that are significant enriched (Benjamini-Hochberg (BH) FDR < 0.1) for colocalizing genes with POAG cross-ancestry GWAS loci and that contain the given colocalizing gene. Similar columns with the prefix 'MGI' refer to Mouse phenotype ontology gene sets taken from the Mouse Genome Informatics (MGI). The cell types in which the given colocalizing gene is nominally enriched (Enrichment P<0.05) based on ECLIPSER analysis of single-nucleus RNA-seq data ('celltypes_Pval05') are given for the anterior segment (AntSeg), retina, macula, and the optic nerve head, optic nerve, and surrounding posterior tissues (ONH) (from Supplementary Data 35). The final columns contain gene sets enriched for cell type-specific colocalizing genes (ECLIPSER P<0.05) in the four ocular tissues, that contain the given colocalizing gene, based on *GeneEnrich* analysis (from Supplementary Data 37). The column definitions of the cell type-specific gene sets are similar to those defined above for all colocalizing e/sGenes. *GeneEnrich* is a hypergenometric based test (one-sided; Methods).

File Name: Supplementary Data 40. Summary table of colocalizing e/sGenes and enriched pathways and cell types for POAG European GWAS loci.
Description: Table that summarizes the pathways and cell types in which the eGenes or sGenes that colocalize with the POAG European GWAS loci belong to or are enriched in. The direction of

effect of each colocalizing e/sQTL relative to POAG risk is given as '+' or '-' in the square brackets in columns I and J. The number preceding the +/- signs specifies the number of tissues in which the eQTL (e) or sQTL (s) has the given direction of effect on the trait. The maximum CLPP or RCP is also given per e/sQTL across all tissues where significant colocalization was found. Significant colocalization was determined at colocalization posterior probability (CLPP) > 0.01 for eCAVIAR and regional colocalization probability (RCP) > 0.1 for *enloc*. 'most_significant_gene_set_membership' contains the gene set/s from Gene Ontology (GO), Reactome, or KEGG with the most significant Empirical gene set enrichment p-value from *GeneEnrich* (from Supplementary Data 31) that contain the colocalizing gene. 'pass_nominal_significance_gene_sets' refers to gene set/s that are nominally enriched (*GeneEnrich* Empirical P<0.05) for colocalizing genes with POAG cross-ancestry GWAS loci and that contain the given colocalizing gene. 'pass_bh_significance_gene_sets' refers to gene set/s that are significant enriched (Benjamini-Hochberg (BH) FDR < 0.1) for colocalizing genes with POAG cross-ancestry GWAS loci and that contain the given colocalizing gene. Similar columns with the prefix 'MGI' refer to Mouse phenotype ontology gene sets taken from the Mouse Genome Informatics (MGI). The cell types in which the given colocalizing gene is nominally enriched (Enrichment P<0.05) based on ECLIPSER analysis of single-nucleus RNA-seq data ('celltypes_Pval05') are given for the anterior segment (AntSeg), retina, macula, and the optic nerve head, optic nerve, and surrounding posterior tissues (ONH) (from Supplementary Data 35). The final columns contain gene sets enriched for cell type-specific colocalizing genes (ECLIPSER P<0.05) in the four ocular tissues, that contain the given colocalizing gene, based on *GeneEnrich* analysis (from Supplementary Data 37). The column definitions of the cell type-specific gene sets are similar to those defined above for all colocalizing e/sGenes. *GeneEnrich* is a hypergenometric based test (one-sided; Methods).

File Name: Supplementary Data 41. Summary table of colocalizing e/sGenes and enriched pathways and cell types for IOP GWAS loci.
Description: Table that summarizes the pathways and cell types in which the eGenes or sGenes that colocalize with the intraocular (IOP) GWAS loci belong to or are enriched in. The direction of effect of each colocalizing e/sQTL relative to POAG risk is given as '+' or '-' in the square brackets in columns I and J. The number preceding the +/- signs specifies the number of tissues in which the eQTL (e) or sQTL (s) has the given direction of effect on the trait. The maximum CLPP or RCP is also given per e/sQTL across all tissues where significant colocalization was found. Significant colocalization was determined at colocalization posterior probability (CLPP) > 0.01 for eCAVIAR and regional colocalization probability (RCP) > 0.1 for *enloc*. 'most_significant_gene_set_membership' contains the gene set/s from Gene Ontology (GO), Reactome, or KEGG with the most significant Empirical gene set enrichment p-value from *GeneEnrich* (from Supplementary Data 32) that contain the colocalizing gene. 'pass_nominal_significance_gene_sets' refers to gene set/s that are nominally enriched (*GeneEnrich* Empirical P<0.05) for colocalizing genes with POAG cross-ancestry GWAS loci and that contain the given colocalizing gene. 'pass_bh_significance_gene_sets' refers to gene set/s that are significant enriched (Benjamini-Hochberg (BH) FDR < 0.1) for colocalizing genes with POAG cross-ancestry GWAS loci and that contain the given colocalizing gene. Similar columns with the prefix 'MGI' refer to Mouse phenotype ontology gene sets taken from the Mouse Genome Informatics (MGI). The cell types in which the given colocalizing gene is nominally enriched (Enrichment P<0.05) based on ECLIPSER analysis of single-nucleus RNA-seq data ('celltypes_Pval05') are given for the anterior segment (AntSeg), retina, macula, and the optic nerve head, optic nerve, and surrounding posterior tissues (ONH) (from Supplementary Data 35). The final columns contain gene sets enriched for cell type-specific colocalizing genes (ECLIPSER P<0.05) in the four ocular tissues, that contain the given colocalizing gene, based on *GeneEnrich* analysis (from Supplementary Data 37). The column definitions of the cell type-specific gene sets

are similar to those defined above for all colocalizing e/sGenes. *GeneEnrich* is a hypergenometric based test (one-sided; Methods).

File Name: Supplementary Data 42. List of negative control traits used to assess specificity of ECLIPSER results.
Description: List of ocular and non-ocular negative control traits with respect to glaucoma used to assess the specificity of the ECLIPSER cell type enrichment results for POAG and IOP in single nucleus eye tissue expression data. The number of genome-wide significant GWAS variants (loci) are reported per trait along with the corresponding references of the GWAS analyzed with ECLIPSER. The GWAS loci were taken from Open Target Genetics or the GWAS meta-analysis consortium, such as the age-related macular degeneration (AMD) genomics consortium (IAMDGC). Number of linkage-disequilibrium (LD)-independent GWAS loci per trait is given based on LD clumping (r^2>0.8) and linking of variants into a single locus based on sharing of e/sQTL target genes mapped to GWAS variants for a given trait, considering all GWASs analyzed for that trait. The number of GWAS loci with genes mapped to them based on LD (r^2>0.8) to fine-mapped e/sVariants (with DAP-G) from 49 GTEx tissues or retina are also provided.

File Name: Supplementary Data 43. Significant ECLIPSER results in eye tissues for negative control traits.
Description: This table contains cell type enrichment results from ECLIPSER for negative control trait GWAS relative to glaucoma (listed in Supplementary Data 42), analyzing single-nucleus RNA-seq data from four ocular tissues: anterior segment, macula, peripheral retina (retina), and the optic nerve head, optic nerve, and surrounding posterior tissues, including peripapillary sclera, peripheral sclera, and choroid (OpticNerveHead). Only cell types with nominal enrichment (Enrichment P<0.05) are shown. Cell types with tissue-wide Benjamini-Hochberg false discovery rate (FDR) below 0.1, are considered significant, correcting for multiple hypothesis testing. Target genes of fine-mapped e/sQTL variants from 49 GTEx tissues or retina that are in LD (r^2>0.8) with the GWAS variants were mapped to the corresponding GWAS loci. Leading edge genes are cell type-specific genes mapped to GWAS loci whose cell type specificity score is equal to or above the 95th percentile enrichment cutoff. These genes are proposed to influence the given trait in the significant enriched cell type/s. e, eQTL; s, sQTL. AMD, age-related macular degeneration.

File Name: Supplementary Data 44. Correlation between number of cells per cell type and ECLIPSER enrichment statistics.
Description: This table contains the Pearson correlation coefficient (r), Pearson correlation of determination (r^2), and Pearson correlation coefficient p-value (two-sided) from comparisons between the number of cells per cell type for four eye tissues, and the ECLIPSER cell type fold-enrichment and p-value. This was computed to evaluate the potential confounding effect of number of cells per cell type on the ECLIPSER cell type enrichment results. POAG, primary open angle glaucoma; IOP, intraocular pressure; VCDR, vertical cup-to-disc ratio; ML, machine learning; EUR, European.

File Name: Supplementary Data 45. Cell type enrichment of POAG and IOP associations in eye tissues using stratified LD score regression (S-LDSC).
Description: Results from cell type enrichment of primary open angle glaucoma (POAG) and intraocular pressure (IOP) associations in four eye tissues using stratified LD score regression (S-LDSC). S-LDSC was used to assess the contribution of genetic variation in cell type-specific genes to trait heritability by only considering common variants within 100kb around genes specifically expressed in the different ocular cell types (fold-change > 1.1 and FDR<0.1) in each of the four single-nucleus eye tissue datasets: anterior segment, macula, retina, and the optic nerve head and surrounding posterior tissues. Computed Z-scores (scaled coefficient per trait

and tissue) were used for heatmap plotting in Supplementary Figure 22. Benjamini-Hochberg false discovery rate (FDR) < 0.1 was considered for multiple hypothesis correction. POAG, primary open angle glaucoma; IOP, intraocular pressure; EUR, European. Cell type abbreviations are described in Supplementary Table 35.

File Name: Supplementary Data 46. Cell type enrichment of POAG and IOP associations in eye tissues using MAGMA.
Description: Results from cell type enrichment of primary open angle glaucoma (POAG) and intraocular pressure (IOP) associations in four eye tissues using MAGMA. MAGMA tests for association between gene association z-scores and average gene expression per cell type, controlling for average gene expression across all cell types per tissue. The four single-nucleus eye tissue datasets analyzed: anterior segment, macula, retina, and the optic nerve head and surrounding posterior tissues. Computed Z-scores (scaled coefficient per trait and tissue) were used for heatmap plotting in Supplementary Figure 22. Benjamini-Hochberg false discovery rate (FDR) < 0.1 was considered for multiple hypothesis correction. EUR, European. Cell type abbreviations are described in Supplementary Table 35.

File Name: Supplementary Data 47. Correlation of cell type enrichment significance between ECLIPSER, S-LDSC and MAGMA.
Description: To evaluate the robustness of the ECLIPSER cell type enrichment results, we compared the cell type enrichment significance between ECLIPSER, S-LDSC and MAGMA, using Pearson correlation coefficient (r), Pearson correlation of determination (r^2), and Pearson correlation coefficient p-values (two-sided).

File Name: Supplementary Data 48. Conditional analysis of cell type enrichment of POAG and IOP associations within each eye tissue using MAGMA.
Description: The table contains the conditional analysis results of cell type enrichment of primary open angle glaucoma (POAG) and intraocular (IOP) associations within four eye tissues using MAGMA. The four single-nucleus RNA-seq ocular datasets analyzed were anterior segment, macula, retina, and the optic nerve head and surrounding posterior tissues. All pairwise combinations of nominally significant (P<0.05) cell types within a given tissue were tested to identify cell types whose trait association signals are independent of the other significant cell type. A proportional significance (PS) of the conditional P-value of a cell type relative to its marginal P-value was computed for each cell type in each cell type pair. Two cell types in a cell type pair with PS≥0.8 were considered independently associated cell types (indep in the Annotation column), and a pair of cell types with PS≥0.5 were considered partial-joint associations. In the case where one cell type had PS≥0.5 and the second cell type a conditional P-value ≥0.05, the first cell type was retained and the second cell type was considered completely dependent on the association of the first cell type (main/drop). For more details see: https://fuma.ctglab.nl/tutorial#celltype.
