# Supplementary material for: Integrating genetic regulation and single-cell expression with GWAS prioritizes causal genes and cell types for glaucoma
Source: Nat Commun. 2024 Jan 9;15:396. doi: 10.1038/s41467-023-44380-y (PMC10776627; doi:10.1038/s41467-023-44380-y)
Supplement: Supplementary file 5 — Reporting Summary [file 41467_2023_44380_MOESM5_ESM.pdf]

nature portfolio

Corresponding author(s): Ayellet V. Segrè

Last updated by author(s): November 20 2023

Reporting Summary

Nature Portfolio wishes to improve the reproducibility of the work that we publish. This form provides structure for consistency and transparency in reporting. For further information on Nature Portfolio policies, see our Editorial Policies and the Editorial Policy Checklist.

Statistics

For all statistical analyses, confirm that the following items are present in the figure legend, table legend, main text, or Methods section.

n/a	Confirmed	
☐	☒	The exact sample size (*n*) for each experimental group/condition, given as a discrete number and unit of measurement
☐	☒	A statement on whether measurements were taken from distinct samples or whether the same sample was measured repeatedly
☐	☒	The statistical test(s) used AND whether they are one- or two-sided<br>*Only common tests should be described solely by name; describe more complex techniques in the Methods section.*
☐	☒	A description of all covariates tested
☐	☒	A description of any assumptions or corrections, such as tests of normality and adjustment for multiple comparisons
☐	☒	A full description of the statistical parameters including central tendency (e.g. means) or other basic estimates (e.g. regression coefficient) AND variation (e.g. standard deviation) or associated estimates of uncertainty (e.g. confidence intervals)
☐	☒	For null hypothesis testing, the test statistic (e.g. *F*, *t*, *r*) with confidence intervals, effect sizes, degrees of freedom and *P* value noted<br>*Give P values as exact values whenever suitable.*
☐	☒	For Bayesian analysis, information on the choice of priors and Markov chain Monte Carlo settings
☒	☐	For hierarchical and complex designs, identification of the appropriate level for tests and full reporting of outcomes
☐	☒	Estimates of effect sizes (e.g. Cohen's *d*, Pearson's *r*), indicating how they were calculated

Our web collection on statistics for biologists contains articles on many of the points above.

Software and code

Policy information about availability of computer code

Data collection	No software was used to collect the data.
Data analysis	We used the following open source code to perform analyses: Colocalization analysis between GWAS and e/sQTL signals were performed with eCAVIAR (eCAVIAR: https://github.com/fhormoz/caviar) and fastENLOC (https://github.com/xqwen/fastenloc). Fine-mapping for the ENLOC analysis was performed using DAP-G (https://github.com/xqwen/dap/tree/master/dap_src). Conditional analysis of the MYOC primary open angle glaucoma GWAS locus was performed using COJO (https://yanglab.westlake.edu.cn/software/gcta/). Two-sample Mendelian Randomization analysis was performed using the TwoSampleMR and MendelianRandomization packages in R (version 4.1.2). We used Plink 1.90 beta (https://www.cog-genomics.org/plink/) to compute linkage disequilibrium between DNA variant pairs. Cell type-specific heritability enrichment of disease associations was performed using stratified LD score regression (S-LDSC v1.0.1; https://github.com/bulik/ldsc). Gene association score correlation with cell type specific gene expression was performed using the regression-based method MAGMA v1.10 (https://ctg.cncr.nl/software/magma). The following custom codes from our lab were used to analyze data in this study: ECLIPSER deposited in GitHub (https://github.com/segrelabgenomics/ECLIPSER) was used to test for enrichment of expression of genes mapped to GWAS loci based on e/sQTLs in specific cell types in single nucleus RNA-seq data from eye tissues. QTLEnrich (https://github.com/segrelabgenomics/QTLEnrich) was used to assess enrichment of genetic associations with POAG and intraocular pressure among eQTLs and sQTLs in GTEx tissues and retina. GeneEnrich used to perform gene set enrichment analysis in the study was deposited on GitHub (https://github.com/segrelabgenomics/GeneEnrich). All MR-related statistical tests were implemented in the following pipeline: https://github.com/segrelabgenomics/TwoSampleMR_pipeline.

For manuscripts utilizing custom algorithms or software that are central to the research but not yet described in published literature, software must be made available to editors and reviewers. We strongly encourage code deposition in a community repository (e.g. GitHub). See the Nature Portfolio guidelines for submitting code & software for further information.

Data

Policy information about availability of data

All manuscripts must include a data availability statement. This statement should provide the following information, where applicable:

- Accession codes, unique identifiers, or web links for publicly available datasets
- A description of any restrictions on data availability
- For clinical datasets or third party data, please ensure that the statement adheres to our policy

All data supporting the findings in this manuscript are available from the links below or in Supplementary Data. The GTEx protected data are available through the database of Genotypes and Phenotypes (dbGaP) (accession no. phs000424.v8). The processed GTEx eQTL and sQTL and EyeGEx retina eQTL summary statistics are available on the GTEx portal (https://gtexportal.org/home/datasets). The snRNA-seq data for the anterior segment and macula are available in Gene Expression Omnibus (GEO) accession number GSE199013, for the optic nerve head and posterior tissues in GSE236566, and for the retina in GSE226108. The processed data of the anterior and posterior segments can be visualized in the Broad Institute's Single Cell Portal at https://singlecell.broadinstitute.org/single_cell/study/SCP1841 and https://singlecell.broadinstitute.org/single_cell/study/SCP2298. The retina Hi-C data is accessible in GEO accession number GSE202471. The GWAS summary statistics for the POAG cross-ancestry GWAS meta-analysis and European subset meta-analysis are accessible in GEO under accession numbers GCST90011770 and GCST90011766, respectively, and for IOP are available from the corresponding publication (Khawaja et al., Nature Genetics 2018). The GWAS loci for complex traits used in the ECLIPSER analysis were downloaded from Open Targets Genetics (https://genetics.opentargets.org/). The gene sets taken from MSigDB, including Gene Ontology, Reactome and KEGG, were downloaded from: http://www.gsea-msigdb.org/gsea/msigdb/collections.jsp, and the mouse phenotype ontology gene sets were downloaded from the Mouse Genome Informatics (MGI) website (http://www.informatics.jax.org/). All the results from our analyses (e.g., colocalization, mendelian randomization, cell type enrichment) can be found in the Supplementary Data. The colocalization and Mendelian randomization results can be viewed on our https://VisionGenomics.org portal.

Research involving human participants, their data, or biological material

Policy information about studies with human participants or human data. See also policy information about sex, gender (identity/presentation), and sexual orientation and race, ethnicity and racism.

Reporting on sex and gender	We analyzed publicly available GWAS and e/sQTL studies (GTEx v8 and EyeGEx), all of which contained a mix of female and male samples. The eye tissue samples used for single-nucleus RNA-sequencing and Hi-C data were taken from postmortem donors of both sexes, as described in the corresponding publications referenced in our manuscript, and as listed in our Methods section. We did not perform separate analyses of males and females for the single cell analyses as the sample sizes are not large enough for sex-specific analyses.
Reporting on race, ethnicity, or other socially relevant groupings	The genome-wide association study (GWAS) meta-analysis of primary open angle glaucoma (POAG) analyzed in this study consisted of three ancestral backgrounds: European, African American, and East Asian. The participants in the intraocular pressure (IOP) GWAS meta-analysis analyzed in this study were primarily from the UK Biobank and of European descent. The ocular tissue donors for the single-nucleus RNA-seq datasets analyzed in this paper were primarily of European descendent.
Population characteristics	The covariates used in the GWAS meta-analyses of primary open angle glaucoma (POAG) and intraocular pressure (IOP), include sex, age, and top genotype principal components, and are described in the corresponding publications. The GWAS was performed on genotype array data that was imputed to the 1000 Genome Project Phase 3 reference panel. The distributions of age and sex of the participants in these studies are provided in the original publications.
Recruitment	No participants were recruited in this study. The collection of ocular tissues from de-identified postmortem donor eyes is described in the relevant publications referenced in our manuscript.
Ethics oversight	This work used publicly available summary statistics data of GWAS of complex human diseases and traits. The eye tissue samples that underwent single-nucleus RNA-sequencing analyzed in this study were collected from de-identified, deceased donors under HIPAA Privacy Rules. Institutional review board (IRB) approvals for post-mortem tissue acquisition at Mass General Brigham (Rapid Autopsy Program) and University of Utah (partnership with Lions International) gave ethical approval for research with the ocular tissue samples. No IRB approval is required for transcriptomic analysis of post-mortem tissues.

Note that full information on the approval of the study protocol must also be provided in the manuscript.

Field-specific reporting

Please select the one below that is the best fit for your research. If you are not sure, read the appropriate sections before making your selection.

[x] Life sciences [ ] Behavioural & social sciences [ ] Ecological, evolutionary & environmental sciences

For a reference copy of the document with all sections, see nature.com/documents/nr-reporting-summary-flat.pdf

Life sciences study design

All studies must disclose on these points even when the disclosure is negative.

Sample size	We analyzed available GWAS data and available eQTL and sQTL datasets from GTEx and EyeGEx. The POAG GWAS meta-analysis sample size was ~34,000 cases and ~349,000 controls, and the IOP GWAS meta-analysis consisted of ~140,000 individuals. The GTEx tissue sample sizes ranged from 73-706, and the EyeGEx retina sample size was 406. The single-nucleus RNA-sequencing (snRNA-seq) datasets of ocular tissues

analyzed in this study were taken from 5-13 donors per tissue. It has been previously shown that snRNA-seq data from at least 10,000 cells that can be obtained from 3-4 donors per tissue is sufficient to characterize the main cell types and cell type-specific gene expression in a range of human tissues (Eraslan et al., Science 376, eabl4290, 2022).

Data exclusions

We excluded the HLA region on chromosome 6 from the primary gene set enrichment analyses (GSEA) to avoid inflating the gene set enrichment results due to a single locus, as multiple eQTLs and sQTLs in this region colocalized with two GWAS loci associated with POAG and IOP (29 and 35 e/sGenes, respectively), likely due to high linkage disequilibrium (LD) in the region. Removal of the HLA region has been commonly done in GSEA analyses of GWAS data. We also provide the GSEA results retaining the HLA region.

Replication

We replicated our single-nucleus RNA-seq retina enrichment results of genes that mapped to glaucoma and IOP GWAS loci in a separate snRNA-seq study of macula samples from 5 post-mortem donors. This replication was performed once and was successful. We did not have a separate snRNA-seq study for the anterior segment for replication. We supported our cell type enrichment results of genes mapped to POAG and IOP GWAS loci found with ECLIPSER using two other GWAS-cell type enrichment methods (S-LDSC and MAGMA). No other experiments were replicated or performed independently in this study.

Randomization

The order of sample processing for library preparation and sequencing in the GTEx project was randomized to avoid batch effects as described in GTEx Consortium, Nature 2017 and GTEx Consortium, Science 2020. Batch effects were controlled for in the e/sQTL regression models by adding inferred hidden covariates on gene expression into the regression model, along with sex and top genotype principal components. The genome-wide association studies (GWAS) analyzed in this paper were designed and genotyped by other consortia (references provided in the paper), but these were not randomized studies by design. For the POAG GWAS, samples were grouped into cases (patients with POAG) and controls (individuals without POAG). For the IOP GWAS, it was a population-based cohort. In the GWAS association analyses, age, sex and top genotype principal components were adjusted for to control for covariates and population stratification. For the experimental design of the single-nucleus RNA-sequencing (snRNA-seq) of ocular tissues, see the associated references in our paper. Differential gene expression analysis between cell types in snRNA-seq data for a given tissue from multiple donors accounted for donor as a covariate.

Blinding

We did not collect the samples for the genome-wide association studies (GWAS) and GTEx data analyzed in this paper. For the POAG GWAS, the investigators were not blinded to group allocation, as they needed to identify cases (patients with POAG) and controls (individual without POAG) within the collected cohorts for study design. Investigators were blinded though to phenotype during genotyping quality control and genetic analyses. Blinding was not necessary for the ocular tissue collection for single nucleus RNA-sequencing as this is not expected to affect the gene expression analysis and all samples were analyzed together in one group.

Reporting for specific materials, systems and methods

We require information from authors about some types of materials, experimental systems and methods used in many studies. Here, indicate whether each material, system or method listed is relevant to your study. If you are not sure if a list item applies to your research, read the appropriate section before selecting a response.

Materials & experimental systems

n/a	Involved in the study
☐	☒ Antibodies
☒	☐ Eukaryotic cell lines
☒	☐ Palaeontology and archaeology
☒	☐ Animals and other organisms
☒	☐ Clinical data
☒	☐ Dual use research of concern
☒	☐ Plants

Methods

n/a	Involved in the study
☒	☐ ChIP-seq
☒	☐ Flow cytometry
☒	☐ MRI-based neuroimaging

Antibodies

Antibodies used

The mouse anti-NeuN monoclonal antibody was added to the dissociated retina and macula samples during fluorescent cytometry cell sorting to enriched for retinal ganglion cells prior to single-nucleus RNA-sequencing. This is because retinal ganglion cells are important for the study of POAG pathophysiology, but are of low abundance in the retina.

Validation

This is described in the original papers that performed the experiments with the anti-NeuN monoclonal antibody (Van Zyl et al., PNAS 2022; Liang et al., Cell Genomics 2023). References are provided in our manuscript.
